# Systematic review of differentially abundant proteins in people with Lewy body dementia

**DOI:** 10.1017/neu.2025.15

**Published:** 2025-03-27

**Authors:** Laura M. Farr, Naomi Thorpe, Ethel M. Brinda, Naser Albalushi, Mohammad Sohail, Anto P. Rajkumar

**Affiliations:** 1 Institute of Mental Health, Mental Health and Clinical Neurosciences Academic Unit, University of Nottingham, Nottingham, UK; 2 Nottinghamshire Healthcare NHS Foundation Trust, Nottingham, UK

**Keywords:** Systematic review, meta-analysis, dementia, Lewy body disease, protein

## Abstract

**Objectives:**

Dementia with Lewy bodies (DLB) and Parkinson’s disease dementia (PDD) are collectively called as Lewy body dementia (LBD). Despite the urgent clinical need, there is no reliable protein biomarker for LBD. Hence, we conducted the first comprehensive systematic review of all Differentially Abundant Proteins (DAP) in all tissues from people with LBD for advancing our understanding of LBD molecular pathology that is essential for facilitating discovery of novel diagnostic biomarkers and therapeutic targets for LBD.

**Methods:**

We identified eligible studies by comprehensively searching five databases and grey literature (PROSPERO protocol:CRD42020218889). We completed quality assessment and extracted relevant data. We completed narrative synthesis and appropriate meta-analyses. We analysed functional implications of all reported DAP using *DAVID* tools.

**Results:**

We screened 11,006 articles and identified 193 eligible studies. 305 DAP were reported and 16 were replicated in DLB. 37 DAP were reported and three were replicated in PDD. Our meta-analyses confirmed six DAP (TAU, SYUA, NFL, CHI3L1, GFAP, CLAT) in DLB, and three DAP (TAU, SYUA, NFL) in PDD. There was no replicated blood-based DAP in DLB or PDD. The reported DAP may contribute to LBD pathology by impacting misfolded protein clearance, dopamine neurotransmission, apoptosis, neuroinflammation, synaptic plasticity and extracellular vesicles.

**Conclusion:**

Our meta-analyses confirmed significantly lower CSF TAU levels in DLB and CSF SYUA levels in PDD, when compared to Alzheimer’s disease. Our findings indicate promising diagnostic biomarkers for LBD and may help prioritising molecular pathways for therapeutic target discovery. We highlight ten future research priorities based on our findings.

## Summations

- This comprehensive systematic review has found 305 differentially abundant proteins (DAP) in people with Dementia with Lewy bodies (DLB). 16 of them were replicated by an independent study, and six were confirmed by our meta-analyses.
- 37 DAP have been reported in people with Parkinson’s disease dementia (PDD). Three of them were replicated and were confirmed by our meta-analyses.
- The reported DAP may contribute to LBD pathology by impacting misfolded protein clearance, dopamine neurotransmission, apoptosis, neuroinflammation, synaptic plasticity and extracellular vesicles.

## Considerations

- We, have included only studies that were published in English. We, did not include studies that investigated animal models or cell lines.
- Most, of the included studies had small sample sizes, and there was substantial heterogeneity among the included studies.
- The majority of the included studies have investigated only cerebrospinal fluid. Studies investigating blood-based DAP in people with LBD are relatively sparse.

## Introduction

Lewy body dementias (LBD) are α-synucleinopathies that account for 15–25% of all dementia. LBD is an umbrella term that includes the following two related dementia: (i) Dementia with Lewy bodies (DLB), and (ii) Parkinson’s disease dementia (PDD). DLB is the second most common neurodegenerative dementia only behind dementia in Alzheimer’s disease (AD) (McKeith *et al*., 2017). DLB is often underdiagnosed and misdiagnosed in clinical settings. A recent survey estimated that nearly 50% of DLB diagnoses had been missed in the United Kingdom (Freer, 2017). Almost two thirds of LBD are misdiagnosed as AD or another illness (Galvin *et al*., 2010; Thomas *et al*., 2017). Failing to diagnose LBD accurately can lead to clinically detrimental consequences because antipsychotic medications, which are often prescribed for managing challenging behaviours associated with AD, can lead to life threatening adverse effects in people with LBD (Spears *et al*., 2019). Moreover, this may delay planning appropriate multidisciplinary clinical care, as people with LBD have faster rate of progression, and require additional care for managing their mobility and autonomic nervous system impairments. Despite the urgent clinical need, we do not have any reliable blood-based or cerebrospinal fluid (CSF) based protein biomarker that can aid distinguishing people with LBD from people with other dementia. Molecular mechanisms underlying neurodegeneration in LBD are relatively underresearched, when compared to those of AD and Parkinson’s disease (PD). Improving our current limited understanding of molecular pathology of LBD is essential for identifying reliable diagnostic biomarkers and novel therapeutic targets for LBD (Walker *et al*., 2015; Fink *et al*., 2020). Hence, we feel the impetus for systematically reviewing all reported Differentially Abundant Proteins (DAP) in people with LBD.

We have already published a systematic review of all LBD genetic association studies (Sanghvi *et al*., 2020) and another systematic review of all gene expression studies in LBD (Chowdhury & Rajkumar, 2020). Genetic associations of LBD with the variants in *APOE*, *GBA*, and *SNCA* encoding α-synuclein (SYUA) have been replicated. Other reported LBD genetic associations have highlighted the contributions of microtubule-associated protein tau (TAU) pathology, ubiquitin proteasome system (UPS), autophagy lysosomal pathway (ALP), and mitochondrial dysfunction towards LBD pathophysiology (Sanghvi *et al*., 2020). Moreover, gene expression studies have reported statistically significant downregulation of several mitochondrial RNA, RNA-mediated gene silencing, and of RNA encoding proteins involved in neuroinflammation, UPS, ALP, and neuregulin signalling (Chowdhury & Rajkumar, 2020). Furthermore, statistically significant differences in alternative splicing of *SNCA, SNCB, PRKN, APP, RELA*, and *ATXN2* transcripts have been reported in people with LBD (Chowdhury & Rajkumar, 2020). Current knowledge on the functional implications of prior reported differentially expressed non-coding RNA in LBD is limited. Hence, reviewing the currently reported DAP in people with LBD is essential for advancing our knowledge on the functional implications of reported genetic associations and of differentially expressed protein-coding RNA in LBD.

Differentially Abundant Proteins usually have more translational potential leading to novel diagnostic biomarkers and therapeutic targets than associated genetic variants and differentially expressed RNA in any disease. However, there has not been a comprehensive systematic review that summarises all reported DAP in all tissues from people with LBD so far. There are reviews focusing on the levels of one or two selected proteins in people with LBD, and they mostly included only studies investigating CSF. Four of them reviewed protein levels in people with DLB (Kasuga *et al*., 2012; Lim *et al*., 2013; Mavroudis *et al*., 2020; Zhang *et al*., 2022) and another reviewed protein abundance in the combined LBD group (Chin *et al*., 2020). Nine more reviews, which investigated protein abundance in other dementia, included people with LBD in their comparison groups (van Harten *et al*., 2011; Wang *et al*., 2015; Bridel *et al*., 2019; Wilczyńska & Waszkiewicz, 2020; Hao *et al*., 2022; Virgilio *et al*., 2022). Most prior reviews focused only on TAU (van Harten *et al*., 2011; Irwin *et al*., 2013; Chin *et al*., 2020; Wilczyńska & Waszkiewicz, 2020; Zhang *et al*., 2022; Zhang *et al*., 2023) and/or SYUA (Irwin *et al*., 2013; Lim *et al*., 2013; Wang *et al*., 2015; Mavroudis *et al*., 2020; Zhang *et al*., 2022) protein levels. Exclusive reviews on CSF and serum Chitinase-3-like-1 (CHI3L1) (Wilczyńska & Waszkiewicz, 2020; Hao *et al*., 2022) and on CSF and plasma neurofilament light polypeptide (NFL) (Bridel *et al*., 2019; Zhao *et al*., 2019) levels have been published. Hence, prior reviews have focused only on four selected proteins (TAU, SYUA, NFL and CHI3L1) so far, and none of them reviewed available evidence of regarding other DAP. Because of such narrow focus, prior reviews have excluded large-scale proteomics data, and they could not do functional enrichment analysis including all reported DAP. Moreover, apart from three narrative reviews (Kasuga *et al*., 2012; Irwin *et al*., 2013; Virgilio *et al*., 2022), prior reviews have focused on evidence from only one selected tissue, mostly CSF. As routine CSF examination is not feasible in mental health settings in many countries including the United Kingdom, there is need for a comprehensive systematic review including blood-based DAP in people with LBD. Therefore, we aimed to complete the first comprehensive systematic review of all reported DAP in all tissues from people with LBD, and to complete functional enrichment analysis including all reported DAP.

## Methods

### Study design

Our systematic review protocol has been registered with the International prospective register of systematic reviews (PROSPERO protocol: CRD42020218889; Available at https://www.crd.york.ac.uk/prospero/display_record.php?RecordID=218889). We have documented all protocol amendments in the PROSPERO database. Supplementary information-1 presents the Preferred Reporting Items for Systematic Review and Meta-Analysis (PRISMA-2020) checklist.

### Search strategy

The following five databases were searched from inception to February 2023 by an information specialist (NT): MEDLINE ALL, Embase, PsycINFO (all via Ovid), Scopus, and Web of Science Core Collection. Grey literature searches from inception to 23rd of Feburary 2023 were completed using the web-based server Turning Research into Practice and the National Grey Literature Collection. Our searches were restricted to papers available in English and human studies. Animal studies were excluded. The search strategy used a combination of free text terms and relevant controlled vocabulary headings customised for each database, as well as advanced search syntax (truncation, Boolean logic AND/OR, and proximity searching) to ensure all relevant studies were identified. The search terms included the following themes, with synonyms to describe each: Dementia; Lewy body; Protein. Supplementary information-2 presents further details of search strategy.

### Eligibility criteria

All original research papers that met the following eligibility criteria were deemed eligible to be included in this systematic review: (i) investigated level of at least one protein in any human tissue and/or biological fluid; (ii) participants in at least one study group were clinically diagnosed to have DLB, PDD or LBD; (iii) participants in the control group were clinically confirmed not to have DLB, PDD or LBD; and (iv) presented differential abundance results comparing protein expression levels between LBD and non-LBD groups. We excluded in vitro studies and studies involving only animal models. We excluded studies that included people with LBD but did not report their results separately. We excluded studies that did not include people with LBD but included only participants with prodromal DLB (Fujishiro *et al*., 2015) and/or people with PD and mild cognitive impairment. We did not exclude any study because of its employed experimental method for measuring protein levels. Hence, we included large-scale proteomic studies and studies reporting targeted protein assays such as Enzyme-linked immunosorbent assays (ELISA).

### Article selection

All identified abstracts were screened by a two-member review team (LF and EMB) using the Rayyan systematic review platform (Ouzzani *et al*., 2016). An independent reviewer (NA) reviewed randomly selected 20% of the abstracts again and confirmed the accuracy of the article selection process. Interrater agreement within the review team was strong (Multiple rater Kappa = 0.82; *Z* = 12.01; *p* < 0.001). We retrieved full texts of the potentially eligible abstracts, and their eligibility was assessed by the three-member review team (LF, EMB and NA). When full text of a potentially eligible abstract was not retrievable, we requested the full text from the corresponding author by email. If the corresponding author did not respond within 14 days, then the abstract was excluded. Whenever there was disagreement regarding the eligibility of a study, the senior author (AR) independently reviewed it and resolved the disagreement through discussion with the review team. After we identified all eligible studies from our database searches, we employed backward citation analysis for identifying additional studies that met our eligibility criteria.

### Quality assessment

We assessed the quality of eligible studies using a tool, adapted from the Quality of genetic association studies tool (Q-Genie) (Sohani *et al*., 2015; Sohani *et al*., 2016). The Q-Genie tool was originally made for assessing the quality of genetic association studies. We adapted the tool (Supplementary information-3) for assessing the quality of studies that investigated differential protein abundance. We assessed the following eleven dimensions of each eligible study using the adapted Q-Genie tool: (i) the rationale for study; (ii) selection and definition of people with LBD; (iii) selection and comparability of comparison groups; (iv) technical assessment of protein expression; (v) non-technical aspects of measurement of differential protein abundance; (vi) other sources of bias; (vii) sample size and power; (viii) a priori planning of statistical analyses; (ix) statistical methods and control for confounding; (x) testing of assumptions and inferences for differential protein abundance analyses; and (xi) appropriate interpretation of the study results. Each dimension was scored on a scale from one (poor) to seven (excellent). Hence, the total scores ranged from 11 to 77. We did not exclude any eligible study because of its quality assessment score.

### Data extraction

The first author (LF) extracted the following data from all included studies: (i) population characteristics including their mean age, country, ethnicity, and indicators of severity of illness such as the mini mental state examination scores (ii) sample size in each group; (iii) method of LBD diagnosis; (iv) investigated protein(s); (v) investigated tissue; (vi) method(s) for analysing differential protein abundance; (vii) differential fold changes between study groups with their p-values or measures of central tendencies and of dispersion in each study group; (viii) statistical correction for multiple testing; and (ix) statistical analyses addressing the effects of potential confounders. When a study did not mention correction for multiple testing, we assumed that the reported results had not undergone statistical correction(s) for multiple testing.

### Data synthesis

We initially conducted narrative synthesis using the extracted data. We first synthesised the data by the type of LBD: DLB, PDD, and LBD. We used the LBD category when a study did not specify whether its participants were diagnosed with PDD or DLB, or when a study did not report DLB and PDD results separately. We then synthesised the reported DAP by the investigated tissue: CSF, brain tissue, blood, serum, and plasma. We deemed the study findings as statistically significant, when reported p-values were less than 0.05. We defined a DAP as a protein that showed statistically significant (*p* < 0.05) differential abundance in an LBD group, when compared to a non-LBD comparison group, in any tissue. We deemed a protein as a replicated DAP, when two or more studies reported statistically significant differential abundance of that protein with the same direction of regulation (consistently increased or reduced levels) in people with LBD in a specific tissue in comparison with similar comparison groups. Where two studies reported contradictory results for a DAP in a specific tissue in relation to similar comparison groups, we considered that as a DAP pending replication. When three or more studies reported a DAP in a specific tissue in relation to similar comparison groups, we conducted appropriate meta-analysis for clarifying the differential abundance of that protein in people with LBD. Then, we graded all reported DAP by their certainty of evidence as; (i) meta-analysis confirmed DAP; (ii) replicated DAP; and (iii) DAP pending replication. Within these three categories, the DAP with higher number of studies were given precedence while interpreting the results. Later, we synthesised the information into summary tables according to the types of LBD, investigated tissues, and the certainty of evidence supporting reported DAP.

### Data analysis

We initially used descriptive statistics to summarise the extracted data. We assessed multiple rater interrater reliability using STATA version 17.1 and its ‘kap’ command. We conducted meta-analyses using STATA version 17.1 and its ‘meta’ command. We assessed the degree of heterogeneity using Higgin’s I^2^ and evaluated publication bias using Funnel plots. Because of high heterogeneity among the included studies, we conducted all meta-analyses as random effects meta-analysis. Studies which reported measures of central tendency and of dispersion of the investigated protein levels in their LBD and non-LBD comparison groups were included in the meta-analyses. Where studies reported only median values and ranges or interquartile ranges (IQR), we assumed the median to represent the mean and calculated standard deviations by either dividing IQR values by 1.35 (Wan *et al*., 2014) or diving range values by four (Hozo *et al*., 2005). Studies that did not report protein expression levels in their LBD and non-LBD comparison groups separately were excluded from the meta-analyses.

### Functional enrichment analysis

We investigated the functional implications of all reported DAP in people with DLB and in people with PDD using the Database for Annotation, Visualization and Integrated Discovery (*DAVID)* (Huang da *et al*., 2009; Sherman *et al*., 2022). *DAVID* groups input terms into biological modules, and identifies enriched biological processes, molecular functions and Kyoto Encyclopaedia of Genes and Genomes (KEGG) pathways. We analysed the list of UniProt Accession numbers of all reported DAP using *DAVID* and identified statistically significant enriched Gene Ontology (GO) terms and functional pathways after Benjamini–Hochberg false discovery rate (FDR) correction at 5%.

### Results

**Figure 1.**
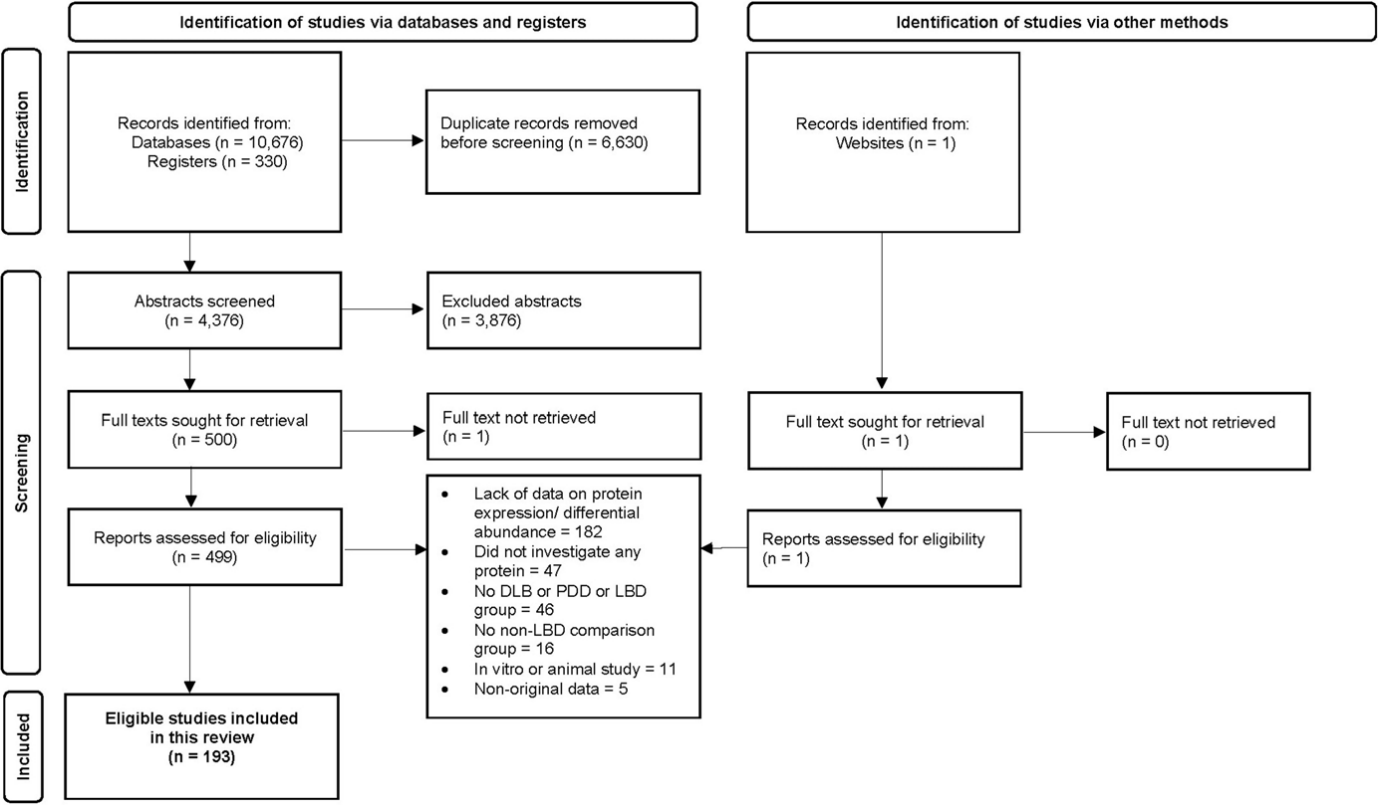
The Preferred Reporting Items for Systematic Reviews and Meta-Analyses flow chart.

We retrieved 10,676 studies by searching online databases and found additional 330 studies from grey literature. We screened 11,006 studies and identified 193 original research studies that met our eligibility criteria. Figure 1 shows the Preferred Reporting Items for Systematic Review and Meta-Analyses (PRISMA) flowchart (Moher *et al*., 2010) presenting the details of our article selection process.

### Study characteristics

Supplementary information-4 lists all 193 included studies. More than half of the included studies (110/193; 56.99%) investigated only people with DLB. 39 studies (20.21%) investigated people with DLB and people with PDD. 24 studies (12.44%) investigated the combined LBD group, and only 20 studies (10.36%) investigated people with PDD exclusively. Nearly two thirds of included studies (127/193; 65.80%) investigated CSF. 37 studies (19.17%) investigated post-mortem brain tissue. Plasma, serum, and whole blood were examined by 17 (8.81%), 12 (6.22%), and 8 (4.15%) studies, respectively. Most of the included studies had small sample sizes, and the average sample size of people with LBD in the included studies was 26. Moreover, the 193 eligible studies included six proteomic studies (Abdi *et al*., 2006; Lehnert *et al*., 2012; Dieks *et al*., 2013; Heywood *et al*., 2015; Bereczki *et al*., 2018; van Steenoven *et al*., 2020).

Supplementary information-5 presents our quality assessment findings. Modified Q-Genie quality assessment total scores ranged from 21 to 65 (Mean 40.64; SD = 7.54). Nearly half of the included studies (98/193; 50.77%) had moderate quality, defined by Modified Q-Genie total scores from 36 to 45. There were 52 (26.94%) low quality (total scores ≤ 35) and 43 (22.28%) good quality (total scores >45) studies. Overall, most of the included studies scored low on study power and on their discussion of sources of bias.

## Differentially abundant proteins in people with DLB

A total of 305 DAP have been reported in all tissues of people with DLB so far (Supplementary information-6). Among them, six (TAU (P10636), SYUA, NFL (P07196), CHI3L1 (P36222), GFAP (P14136), and CLAT (P28329)) were confirmed by our meta-analyses. Table 1 and supplementary information-7 present further details of our meta-analyses including studies that investigated people with DLB. There were nine more replicated DAP in CSF of people with DLB (Table 2), and one more replicated DAP (SNAP25, P60880) in post-mortem DLB brains. There was no replicated blood-based DAP in people with DLB. Among the 289 remaining DAP that were pending replication, 253 were identified in CSF of people with DLB. 26, 13, 11, and eight DAP pending replication have been reported in post-mortem brain tissue, plasma, serum, and whole blood of people with DLB, respectively.

**Table 1.** Differentially abundant proteins, confirmed by meta-analyses, in people with dementia with Lewy bodies

CG	Protein (UniProt ID)	Tissue	Studies	Effect size	95%CI	*p*-value
HC	TAU (P10636)	CSF	70	0.47	0.36–0.58	<0.01
HC	SYUA (P37840)	CSF	23	−0.39	−0.70–−0.07	0.02
HC	NFL (P07196)	CSF	14	1.19	0.55–1.83	<0.01
HC	CHI3L1 (P36222)	CSF	3	0.53	0.09–0.97	0.02
HC	GFAP (P14136)	CSF	3	0.99	0.56–1.41	<0.01
OD	TAU (P10636)	CSF	67	−0.89	−1.00–−0.79	<0.01
OD	SYUA (P37840)	CSF	21	−0.37	−0.69–−0.05	0.02
OD	NFL (P07196)	CSF	7	−0.32	−0.53–−0.12	<0.01
AD	TAU (P10636)	CSF	63	−1.02	−1.15–−0.90	<0.01
AD	SYUA (P37840)	CSF	21	−0.36	−0.68–−0.04	0.03
HC	CLAT (P28329)	Brain Tissue	5	−3.64	−6.75–−0.54	0.02

CG: Comparison group; Studies: Number of studies that investigated the differential abundance of the specific protein in people with dementia with Lewy Bodies (DLB); Effect size: Standardised Mean Difference (Hedges’ g); HC: When compared to people without cognitive impairment; OD: When compared to people with other dementia; AD: When compared to people with Alzheimer’s Disease; CSF: cerebrospinal fluid.

**Table 2.** Other replicated* differentially abundant proteins in cerebrospinal fluid (CSF) of people with dementia with Lewy bodies (DLB)

Protein (Uniprot ID)	Healthy controls	Other dementia
A1AT (P01009)	↑↑	NS
AACT (P01011)	↑↑	NS
BNC2 (Q6ZN30)	↓↓	–
CMGA (P10645)	↑↑	–
PROF2 (P35080)	↑↑	–
PTPR2 (Q92932)	↓↓	–
SMS (P61278)	↓↓	–
UCHL1 (P09936)	↑↑	↓NS
NPTXR (O95502)	↓↓	–

* At least two independent studies have reported statistically significant (*p* < 0.05) differential abundance in the same direction in the CSF of people with DLB; Healthy controls: When compared to people without cognitive impairment; Other Dementia: When compared to people with other dementia; ↑: Statistically significant increased level of expression when compared to the comparison group in at least one of the included studies; ↓: Statistically significant decreased level of expression when compared to the comparison group in at least one of the included studies; NS: Statistically not significant result; -: Has not been investigated in any included study.

### DAP in CSF of people with DLB

267 DAP have been reported in CSF of people with DLB so far. 14 of them have been replicated by two or more studies (Table 1 and Table 2). Among them, five DAP were confirmed by our meta-analyses (Table 1 and supplementary information-7). Differential abundance of TAU in CSF of people with DLB have been extensively investigated in 70 independent samples (Supplementary information-4 and supplementary information-7). Our random-effects meta-analysis confirmed that CSF TAU levels were significantly higher in people with DLB, when compared to people without cognitive impairment (Standardised mean difference (SMD) = 0.47; 95%CI 0.36 – 0.58; *p* < 0.01) (Supplementary information-7.1.1.1). However, our meta-analysis showed that CSF TAU levels were significantly decreased in people with DLB, when compared to people with other dementia (SMD = −0.89; 95%CI −1.00–−0.78; *p* < 0.01) (Supplementary information-7.1.1.2), especially those with AD (SMD = −1.02; 95%CI −1.15–−0.90; *p* < 0.01) (Supplementary information-7.1.1.3).

**Figure 2.**
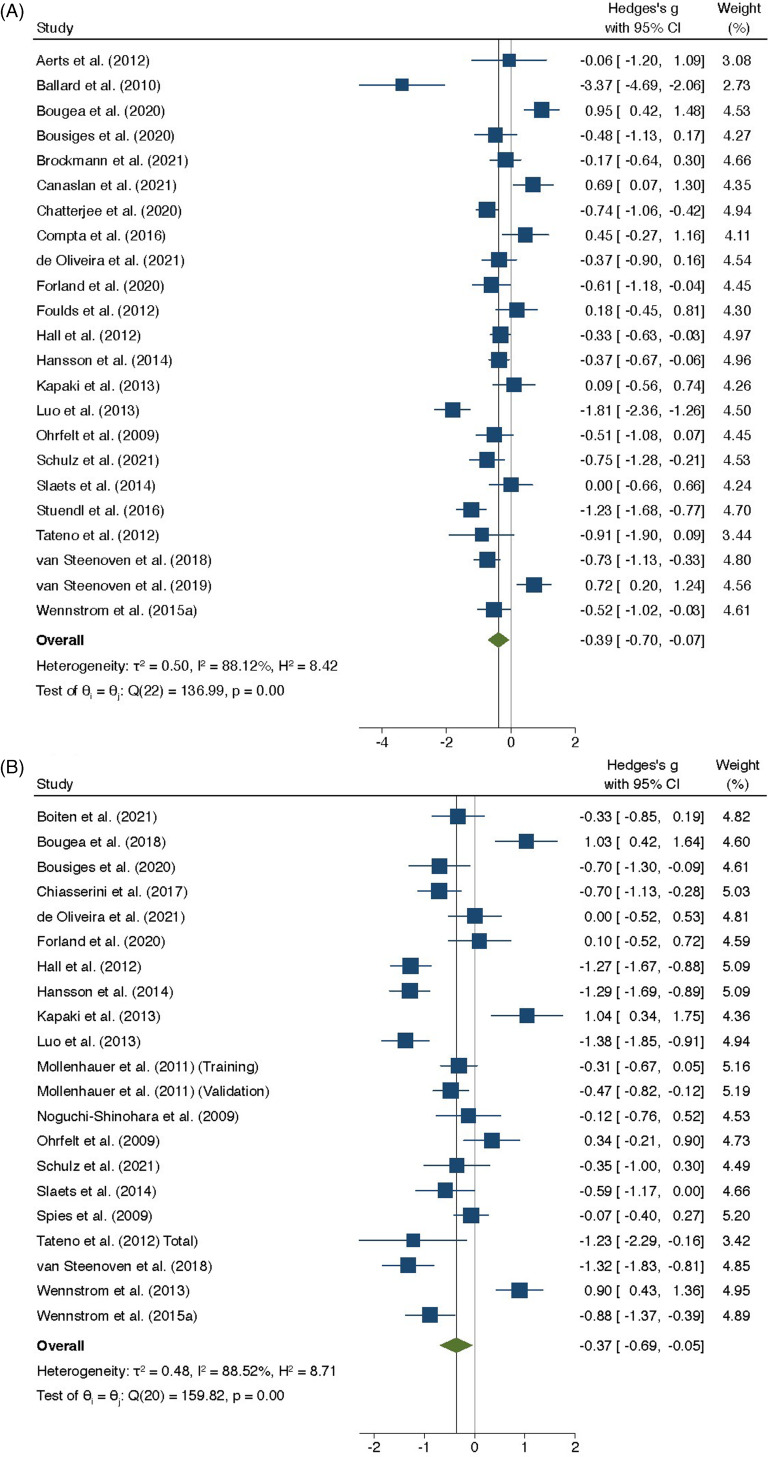
Random-effects meta-analysis of studies that have investigated the differential abundance of α-synuclein in cerebrospinal fluid (CSF) of people with dementia with Lewy bodies (DLB). (2A) Random-effects meta-analysis of studies that have investigated the differential abundance of α-synuclein in CSF of people with DLB, when compared to people without cognitive impairment. (2B) Random-effects meta-analysis of studies that have investigated the differential abundance of α-synuclein in CSF of people with DLB, when compared to people with other dementia.

α-synuclein (SYUA) is the second most investigated protein in CSF of people with DLB. Our random effects meta-analysis showed that CSF SYUA levels were significantly less in people with DLB, when compared to people without cognitive impairment (SMD = −0.39; 95%CI −0.70–−0.07; *p* = 0.02) (Figure 2A). Similarly, another meta-analysis revealed that CSF SYUA levels were significantly less in people with DLB, when compared to people with other dementia (SMD = −0.37; 95%CI −0.69–−0.05; *p* = 0.02) (Figure 2B). We conducted another meta-analysis that confirmed statistically significant reduction of CSF SYUA levels in people with DLB, when compared to those of people with AD (SMD = −0.36; 95%CI −0.68–−0.04; *p* = 0.03) (Supplementary information-7.1.2.3).

Our meta-analysis confirmed that CSF Neurofilament light polypeptide (NFL) levels were significantly higher in people with DLB, when compared to those without cognitive impairment (SMD = 1.19; 95%CI 0.55–1.83; *p* < 0.01) (Supplementary information-7.1.3.1). However, our next meta-analysis showed that CSF NFL levels were significantly lower in people with DLB, when compared to people with other dementia including those with AD and Frontotemporal Dementia (SMD = −0.32; 95%CI −0.53–−0.12; *p* < 0.01) (Supplementary information-7.1.3.2). Nevertheless, when we conducted a meta-analysis including only the studies that directly compared people with DLB with those with AD, we found that CSF NFL levels in people with DLB were not significantly different from those of people with AD (SMD = −0.13; 95%CI −0.41–0.15; *p* = 0.38) (Supplementary information-7.1.3.3). Moreover, our meta-analysis showed that CSF CHI3L1 levels were significantly higher in people with DLB, when compared to those of people without cognitive impairment (SMD = 0.53; 95%CI 0.09−0.97; *p* = 0.02) (Supplementary information-7.1.4.1). Subsequent meta-analysis revealed that CSF CHI3L1 levels in people with DLB were not significantly different from those of people with AD (SMD = −0.37; 95%CI 0.79–0.06; *p* = 0.09) (Supplementary information-7.1.4.2) (Bartres-Faz *et al*., 2015). Furthermore, we conducted another meta-analysis and confirmed that CSF Glial fibrillary acidic protein (GFAP) levels were significantly higher in people with DLB, when compared to those of without cognitive impairment (SMD = 0.99; 95%CI 0.56–1.41; *p* < 0.01) (Supplementary information-7.1.5.1). Besides, we performed two more meta-analyses that did not confirm statistically significant differential abundance of Fatty Acid-binding protein, heart (FABPH, P05413) (SMD = 0.51; 95%CI −1.32–0.31; *p* = 0.22) (Supplementary information-7.1.6.1) and of S100B (P04271) (SMD = 0.53; 95%CI −0.36−1.42; *p* = 0.24) (Supplementary information-7.1.7.1) protein levels in CSF of people with DLB, when compared to people with AD and to those without cognitive impairment, respectively.

This systematic review found the following five replicated DAP, Alpha-1 antitrypsin (A1AT), Alpha-1-antichymotrypsin (AACT), Chromogranin-A (CMGA), Profilin-2 (PROF2), and Ubiquitin carboxyl-terminal hydrolase isozyme L1 (UCHL1), which were significantly more abundant in CSF of people with DLB, when compared to people without cognitive impairment (Table 2). Moreover, significantly reduced expression levels of the following four DAP, Zinc finger protein basonuclin-2 (BNC2), Receptor-type tyrosine-protein phosphatase N2 (PTPR2), Somatostatin (SMS), and Neuronal pentraxin receptor (NPTXR), in CSF of people with DLB, when compared to people without cognitive impairment, have been replicated by independent studies (Table 2).

### DAP in post-mortem brain tissue from people with DLB

Twenty eight DAP have been reported in post-mortem DLB brains. Among them, only one was confirmed by our meta-analysis (Table 1 and supplementary information-7), and another one has been replicated by independent studies. Differential abundance of 26 reported DAP in post-mortem DLB brains have not been replicated so far (supplementary information-6). Choline-O-Acetyltransferase (CLAT) was the only DAP that was confirmed by our meta-analysis. Our random-effects meta-analysis confirmed that CLAT expression levels were significantly lower in post-mortem DLB brains, when compared to post-mortem brains of people without cognitive impairment (SMD = −3.64; 95%CI −6.75–−0.54; *p* = 0.02) (Supplementary information-7.2.2.1). Subsequent meta-analysis showed that CLAT expression levels in post-mortem DLB brains were not significantly different from those of post-mortem AD brains (SMD = −1.17; 95%CI −2.36–0.03; *p* = 0.06) (Supplementary information-7.2.2.2).

The second replicated DAP in post-mortem DLB brains was Synaptosome Associated Protein 25 (SNAP25). Two studies included in this systematic review have reported significantly reduced SNAP25 expression levels in post-mortem DLB brains, when compared to post-mortem brains of people without cognitive impairment (Mukaetova-Ladinska *et al*., 2013; Bereczki *et al*., 2016). However, both studies investigated different areas of brain tissue. One study showed that SNAP25 was significantly less abundant in the middle dorsolateral prefrontal cortex, ventral anterior cingulate cortex, and left supramarginal gyrus of post-mortem DLB brains (Bereczki *et al*., 2016). The second study reported that SNAP25 was significantly less abundant in the middle and lower occipital cortex of post-mortem DLB brains, when compared to post-mortem brains of people without cognitive impairment (Mukaetova-Ladinska *et al*., 2013).

Unlike differential abundance of SYUA in CSF of people with DLB, our meta-analyses did not confirm statistically significant differential abundance of SYUA in post-mortem DLB brains, when compared to post-mortem brains of people without cognitive impairment (SMD = 1.66; 95%CI −1.58–4.90; *p* = 0.32) (Supplementary information-7.2.1.1), of people with other dementia (SMD = 2.16; 95%CI −0.43–4.74; *p* = 0.10) (Supplementary information-7.2.1.2) and of people with AD (SMD = 3.35; 95%CI −2.02–8.72; *p* = 0.22) (Supplementary information-7.2.1.3). However, a study included in this systematic review has reported significantly increased SYUA levels in only caudate and putamen tissue from post-mortem DLB brains, when compared to those from people without cognitive impairment (Tu *et al*., 2022).

### Blood-based DAP in with DLB

This systematic review did not find any replicated DAP in plasma, serum or whole blood of people with DLB. Our meta-analysis showed that SYUA levels in plasma or serum of people with DLB were not significantly different from those of people without dementia (SMD = 0.12; 95%CI –1.11–1.36; *p* = 0.84) (Supplementary information-7.3.1.1). Differential abundance of 32 reported blood-based DAP in people with DLB have not been replicated by an independent study so far (supplementary information-6). Among the 13 DAP pending replication in plasma of people with DLB, five were interleukin proteins. Those interleukins were significantly less abundant in plasma of people with DLB, when compared to people with mild cognitive impairment (King *et al*., 2018; Usenko *et al*., 2020).

## Differentially abundant proteins in people with PDD

37 DAP have been reported in all tissues of people with PDD (Supplementary information-6 and supplementary information-8). Among them, three CSF DAP (TAU, SYUA, and NFL) were confirmed by our meta-analyses. Table 3 and supplementary information-8 present further details of our meta-analyses of studies that investigated people with PDD. There was no replicated DAP in post-mortem PDD brains, and there was no replicated blood-based DAP in people with PDD. Differential abundance of 34 reported DAP in people with PDD have not been replicated so far (supplementary information-6).

**Table 3.** Differentially abundant proteins, confirmed by meta-analyses, in cerebrospinal fluid (CSF) of people with Parkinson’s Disease Dementia (PDD)

CG	Protein (UniProt ID)	Studies	Effect size	95%CI	*p*-value
HC	TAU (P10636)	21	0.27	0.02–0.53	0.03
HC	NFL (P07196)	4	1.09	0.86–1.32	<0.01
OD	TAU (P10636)	13	−0.94	−1.17–−0.72	<0.01
AD	TAU (P10636)	13	−0.99	−1.19–0.79	<0.01
AD	SYUA (P37840)	5	−0.83	−1.58–−0.07	0.03

CG: Comparison group; Studies: Number of studies that investigated the differential abundance of the specific protein in people with PDD; Effect size: Standardised Mean Difference (Hedges’ g); HC: When compared to people without cognitive impairment; OD: When compared to people with other dementia; AD: When compared to people with Alzheimer’s Disease.

### DAP in CSF of people with PDD

**Figure 3.**
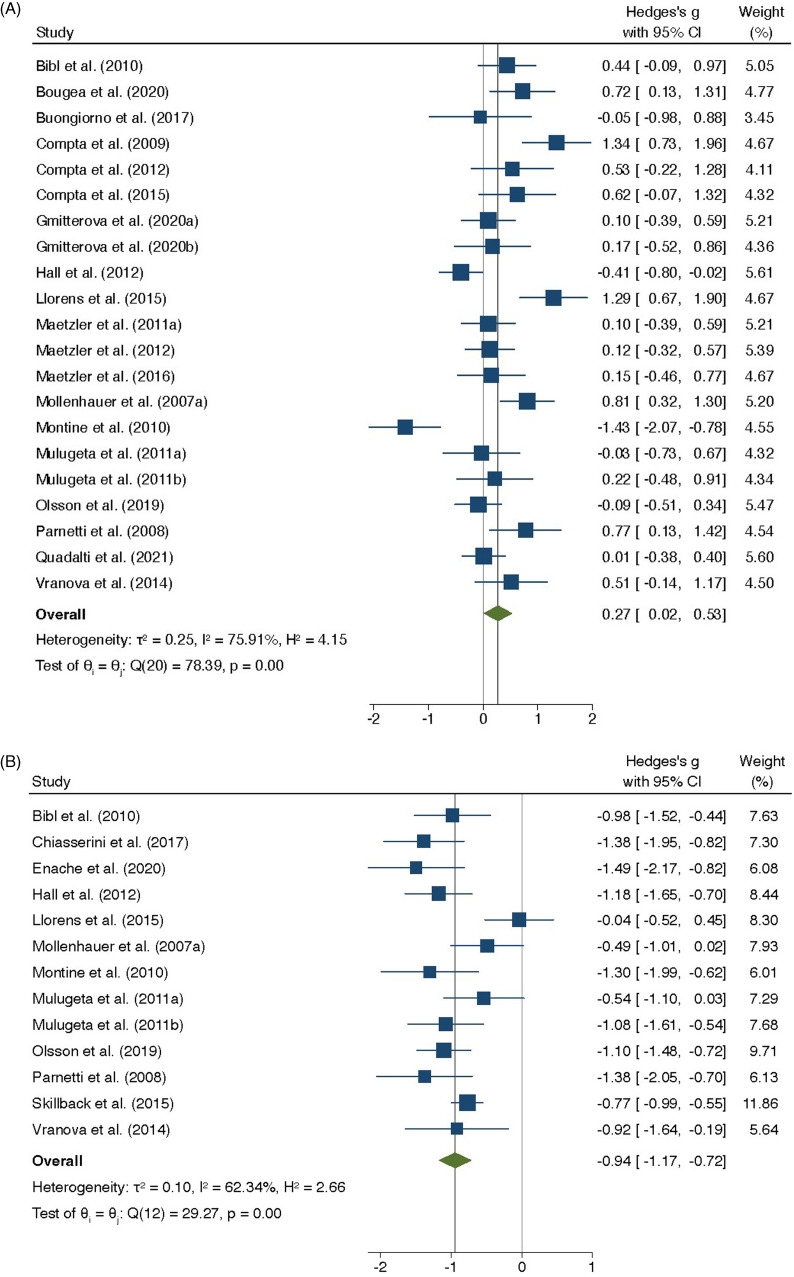
Random-effects meta-analysis of studies that have investigated the differential abundance of microtubule-associated tau protein (TAU) in cerebrospinal fluid (CSF) of people with Parkinson’s disease dementia (PDD). (3A) Random-effects meta-analysis of studies that have investigated the differential abundance of TAU in CSF of people with PDD, when compared to people without cognitive impairment. (3B) Random-effects meta-analysis of studies that have investigated the differential abundance of TAU in CSF of people with PDD, when compared to people with other dementia.

Fourteen DAP have been reported in CSF of people with PDD so far. Three of them were confirmed by our meta-analyses (Table 3 and supplementary information-8). The remaining 11 reported DAP in CSF of people with PDD have not been replicated by an independent study. Our meta-analysis confirmed that CSF TAU levels were significantly higher in people with PDD, when compared to people without dementia (SMD = 0.27; 95%CI 0.02–0.53; *p* = 0.03) (Figure 3A and Supplementary information-8.1.1.1). However, further meta-analyses showed that CSF TAU levels were significantly lower in people with PDD, when compared to people with other dementia (SMD = −0.94; 95%CI −1.17– −0.72; *p* < 0.01) (Figure 3B and Supplementary information-8.1.1.2) and to people with AD (SMD = −0.99; 95%CI −1.19–−0.79; *p* < 0.01) (Supplementary information-8.1.1.3).

We conducted more meta-analyses and confirmed that CSF SYUA levels were significantly lower in people with PDD, when compared to people with AD (SMD = −0.83; 95%CI −1.58– −0.07; *p* = 0.03) (Supplementary information-8.1.2.2). However, differential abundance of SYUA in CSF of people with PDD, when compared to people without dementia, was not statistically significant (SMD = −0.34; 95%CI−0.67 – 0.00; *p* = 0.05) (Supplementary information-8.1.2.1). NFL was the third replicated DAP in CSF of people with PDD. Our meta-analysis confirmed that CSF NFL levels were significantly higher in people with PDD, when compared to people without dementia (SMD = 1.09; 95%CI 0.86–1.32; *p* < 0.01) (Supplementary information-8.1.3.1). Among the 11 reported DAP pending replication in CSF of people with PDD, S100B was significantly more abundant in people with PDD than in people without dementia (Gmitterova *et al*., 2020).

### DAP in post-mortem brain tissue from people with PDD

Studies, included in this systematic review, have reported 17 DAP in post-mortem PDD brains. None of them was confirmed by our meta-analysis (supplementary information-8), and differential abundance of all 17 reported DAP in post-mortem PDD brains have not been replicated (supplementary information-6). Our meta-analyses showed that SYUA levels in post-mortem PDD brains were not significantly different from those of people without dementia (SMD = 0.18; 95%CI −1.58–1.93; *p* = 0.83) (Supplementary information-8.2.1.1) and from those of people with other dementia (SMD = 2.15; 95%CI −0.42–4.73; *p* = 0.10) (Supplementary information-8.2.1.2). Among the DAP pending replication in post-mortem PDD brains, Synaptophysin (SYPH, P08247), Allograft inflammatory factor 1 (AIF1, P55008), and Discoidin domain-containing receptor 2 (DDR2, Q16832) were significantly more abundant in PDD brains, when compared to brains of people without dementia. Disks large homologue 4 (DLG4, P78352) and NAD-dependent protein deacetylase sirtuin-1 (SIR1, Q96EB6) proteins were reportedly significantly less abundant in post-mortem PDD brains than in post-mortem brains of people without dementia.

### Blood-based DAP people with PDD

This systematic review did not find any replicated blood-based DAP in people with PDD. Differential abundance of ten reported blood-based DAP in people with PDD have not been replicated so far (supplementary information-6). Among the six reported DAP pending replication in plasma of people with PDD, plasma NFL levels were significantly decreased in PDD when compared to people with AD (Lin *et al*., 2018). However, plasma NFL levels in PDD were significantly higher than those in PD (Lin *et al*., 2018). The reported findings on the differential abundance of NFL in plasma of people with PDD, when compared to people without dementia, are contradictory. A study has reported significantly more abundant plasma NFL and another study has found significantly less abundant plasma NFL in people with PDD, when compared to people without dementia (Lin *et al*., 2018; Quadalti *et al*., 2021).

### DAP in people with LBD

The included studies that did not present the results from people with DLB and people with PDD separately have reported 62 DAP in CSF of people with LBD. Apart from TAU, differential abundance of the remaining 61 reported DAP in CSF have not been replicated (Supplementary information-6). CSF TAU levels were reportedly higher in people with LBD, when compared to people without dementia. They were found to be significantly lower in people with LBD, when compared to people with other dementia. The reported differences between CSF TAU levels in people with LBD and people with AD were not statistically significant. Moreover, a study reported statistically significant increased CSF NFL levels (Ashton *et al*., 2021), and another study found statistically significant decreased CSF NFL levels (Diekämper *et al*., 2021) in people with LBD, when compared to people without dementia. Besides, this systematic review did not find any additional replicated blood-based DAP or DAP in post-mortem brains from the studies that did not present the results from people with DLB and people with PDD separately. Dipeptidyl peptidase 2 (DPP2, Q9UHL4) was the only additional DAP pending replication in post-mortem LBD brains, and it was shown to be significantly decreased in frontal cortex of people with LBD (Mantle *et al*., 1995). Furthermore, there were six additional reported blood-based DAP pending replication in people with LBD (Supplementary information-6). Blood SYUA and TAU levels were significantly decreased in people with LBD, when compared to people without dementia (Daniele *et al*., 2021). Serum β-Synuclein (SYUB, Q16143) levels were found to be significantly less in people with LBD, when compared to those with Creutzfeldt–Jakob disease (Oeckl *et al*., 2020). Plasma GFAP (Chouliaras *et al*., 2022), NFL (Chouliaras *et al*., 2022), and Major prion protein (PRIO, P04156) (Llorens *et al*., 2019) levels were reportedly significantly higher in people with LBD, when compared to people without dementia.

### Posttranslational protein modifications (PTM) in people with LBD

Detailed analysis of PTM of the reported DAP is beyond the scope of this systematic review. However, 90 included studies have reported PTM of investigated proteins in people with LBD. CSF Phosphorylated TAU 181 (p-TAU181) levels were found statistically significantly decreased in people with DLB, when compared to people with AD. Plasma p-TAU levels were significantly increased in people with DLB, when compared to people without dementia (Alcolea *et al*., 2021; Gonzalez *et al*., 2022). However, the findings comparing plasma p-Tau levels between DLB and AD were inconclusive (Alcolea *et al*., 2021; Chouliaras *et al*., 2022; Gonzalez *et al*., 2022). CSF phosphorylated neurofilament heavy polypeptide levels were significantly increased in people with DLB, when compared to people without dementia (de Jong *et al*., 2007; Schulz *et al*., 2021), and they did not differ significantly from those of people with other dementia. Moreover, included studies have investigated oligomeric SYUA levels specifically. CSF oligomeric SYUA levels were significantly higher in people with PDD, when compared to people without dementia and to people with AD (Compta *et al*., 2014). The findings comparing CSF oligomeric SYUA levels in people with DLB with those in people without dementia remain inconclusive (Foulds *et al*., 2012; Hansson *et al*., 2014; van Steenoven *et al*., 2020). Furthermore, phosphorylated amyloid-β precursor protein (p-APP) was significantly higher in temporal cortex of post-mortem DLB and PDD brains, when compared to brains of people without dementia (Tu *et al*., 2022). p-APP was significantly increased in caudate nucleus of only DLB brains, when compared to people without dementia (Tu *et al*., 2022). Similarly, phosphorylated Calmodulin-dependent protein kinase-II was significantly decreased in the left supramarginal gyrus of post-mortem DLB brains, when compared to people without dementia (Vallortigara *et al*., 2014). It was significantly decreased in middle dorsolateral prefrontal cortex and left supramarginal gyrus of post-mortem PDD brains, when compared to brains of people without dementia (Vallortigara *et al*., 2014).

### Functional enrichment analyses of reported DAP

Supplementary information-9 and Supplementary information-10 present the FDR (5%) corrected results from *DAVID* functional enrichment analyses including all reported DAP in people with DLB and in people with PDD, respectively. The reported DAP in people with DLB were significantly (Benjamini–Hochberg FDR (5%) corrected p-values <0.05) enriched among the proteins involved in 69 biological processes including cell adhesion, immune response, inflammatory response, microglial cell activation, neutrophil chemotaxis, glycolysis, signal transduction, nitric oxide biosynthesis, regulation of protein phosphorylation, regulation of apoptosis, regulation of gene expression, memory, and ageing. They were significantly enriched among the proteins involved in 18 molecular functions including cytokine activity, protease binding, protein binding, serine-type endopeptidase inhibitor activity, lipid binding, and chaperone binding. Extracellular region, extracellular space, extracellular exosome, endoplasmic reticulum lumen, and blood microparticle were the top-5, ranked by the FDR-corrected p-values, among the 39 significantly enriched cellular components. The reported DAP in people with DLB were significantly enriched among the proteins involved in 37 KEGG functional pathways including complement and coagulation cascades, inflammatory bowel disease, glycolysis, IL-17 signalling pathway, HIF-1 signalling pathway, cell adhesion, NOD-like receptor signalling pathway, C-type lectin receptor signalling pathway, and pathways of neurodegeneration (Supplementary information-9).

The reported DAP in people with PDD were significantly enriched among the proteins involved in 22 biological processes including microglial cell activation, regulation of neuron death, nitric oxide biosynthesis, regulation of chemokine production, regulation of lipid storage, dopamine biosynthesis, humoral immune response, synaptic vesicle maturation, regulation of gene expression, regulation of apoptosis, astrocyte activation, long-term neuronal synaptic plasticity, regulation of beta-amyloid formation, synaptic vesicle exocytosis, and inflammatory response. They were significantly enriched among 15 cellular components including neuron projection, synaptic vesicle, axon, extracellular region, extracellular space, neuronal cell body, mitochondrion, glutamatergic synapse, and synaptic vesicle membrane. The reported DAP in people with PDD were significantly enriched among the proteins involved in 10 KEGG functional pathways including pathways of neurodegeneration, Parkinson disease, Rheumatoid arthritis, IL-17 signalling pathway, NOD-like receptor signalling pathway, and cytokine-cytokine receptor interaction (Supplementary information-10).

## Discussion

This is the first comprehensive systematic review of all reported DAP in all tissues from people with LBD. To the best of our knowledge, our meta-analyses are the first to confirm significantly reduced CSF TAU levels in people with DLB, when compared to people with AD, and to confirm significantly reduced CSF α-synuclein (SYUA) levels in people with PDD, when compared to people with AD. This systematic review is the first to present a comprehensive list of all replicated DAP in people with DLB and in people with PDD. We have listed all reported DAP in LBD and have investigated their functional implications. The strengths of this systematic review include its broad eligibility criteria, following PRISMA guidelines, searching multiple databases including grey literature, completing multiple meta-analyses, and employing functional enrichment analyses. Its limitations are excluding studies that were not published in English, excluding studies that investigated animal models or cell lines, not excluding studies that had poor quality assessment scores, assuming Gaussian distribution for studies that reported only median values, combining all brain regions together in our meta-analyses, and substantial heterogeneity among the included studies. Excluding non-English studies might have excluded relevant research and biased our results, and it may limit the generalisability of our findings. The majority of the included studies were at risk of type-II error because of small sample sizes and lack of reporting of power analyses. They also have the risk of type-I error due to the lack of appropriate multiple testing corrections. Moreover, there was high heterogeneity among the included studies because they differed widely on their population characteristics, LBD case definitions, selection of controls, experimental methods for measuring differential protein abundance, and statistical analyses.

Accurately differentiating people with DLB from people with AD is the key clinical challenge (Galvin *et al*., 2010; Thomas *et al*., 2017). Prior reviews on CSF SYUA levels in people with DLB have included only ELISA studies (Zhang *et al*., 2022) or have excluded studies that did not report mean values in each study group (Wang *et al*., 2015). Our comprehensive meta-analyses confirmed that CSF SYUA levels are significantly lower in people with DLB than in people with AD (Lim *et al*., 2013; Wang *et al*., 2015; Mavroudis *et al*., 2020; Zhang *et al*., 2022). They confirmed that CSF SYUA levels in people with DLB are significantly lower than those in people without dementia (Zhang *et al*., 2022). These findings set the stage for future clinical studies investigating the diagnostic biomarker potential of CSF α-synuclein for aiding DLB diagnosis in clinical settings. However, similar to a prior systematic review (Kasuga *et al*., 2012), our meta-analysis showed that plasma or serum SYUA levels in people with DLB did not differ significantly from those of people without dementia. α-synuclein oligomerisation is the key initial step in the formation of Lewy bodies (Beyer *et al*., 2009), and future blood-based DLB biomarker studies may focus on oligomeric SYUA.

Tau pathology is likely to contribute more towards AD than towards DLB pathology (Arezoumandan *et al*., 2024), and differential abundance of total TAU and of p-TAU may help differentiating people with DLB from people with AD accurately. We have presented the hitherto most comprehensive meta-analysis of CSF TAU levels in people with DLB. Our meta-analyses confirmed that CSF TAU levels are significantly higher in people with DLB than in people without dementia, and that they are significantly lower in people with DLB than in people with other dementia, especially AD (van Harten *et al*., 2011; Zhang *et al*., 2022). These findings highlight the need for large clinical studies investigating the diagnostic biomarker potential of CSF TAU levels for improving DLB diagnosis in clinical settings. This systematic review identified promising preliminary evidence indicating the diagnostic biomarker potential of p-TAU, and supports further studies focusing on p-TAU levels (Alcolea *et al*., 2021; Chouliaras *et al*., 2022; Gonzalez *et al*., 2022).

NFL is a general marker for axonal damage and neuronal cell death in many neurodegenerative disorders (Jung & Damoiseaux, 2024). A prior review has reported that CSF NFL levels were significantly higher in people with DLB than in people without dementia (Zhao *et al*., 2019). However, another review that analysed people with DLB and people with PD or PDD together did not find statistically significant difference in NFL levels (Bridel *et al*., 2019). Our meta-analyses clarified that CSF NFL levels are significantly higher in people with DLB than in people without dementia, and that they are significantly lower in people with DLB than in people with other dementia including AD. Our meta-analysis showed that CSF NFL levels did not differ significantly between DLB and AD, so CSF NFL levels may not help distinguishing people with DLB from people with AD (Baiardi *et al*., 2022; Verberk *et al*., 2022). Moreover, CHI3L1 is of primarily astrocytic origin, and it is a well-known biomarker for neuroinflammation and neurodegeneration in AD (Connolly *et al*., 2023). A prior meta-analysis that investigated multiple neurogenerative diseases has reported significantly higher CSF CHI3L1 levels in people with DLB or other dementia, when compared to people without dementia (Hao *et al*., 2022). Our meta-analysis confirmed that CSF CHI3L1 levels are significantly higher in DLB than in people without dementia, and that they did not differ significantly from people with AD.

We have presented the first meta-analyses of CSF GFAP, FABPH, and S100B levels in people with DLB. Our meta-analysis showed that CSF GFAP levels were significantly higher in people with DLB than in people without dementia and highlighted the need for investigating whether CSF GFAP levels may help differentiating DLB from other dementia. Besides, statistically significant differential abundance of A1AT, AACT, CMGA, PROF2, UCHL1, BNC2, PTPR2, SMS, and NPTXR in CSF of people with DLB, when compared to people without dementia, have been replicated. Our findings highlight the need for further research investigating the diagnostic biomarker potential of these nine DAP for differentiating people with DLB from people with AD or other dementia. Moreover, another CSF proteomics study that compared people with DLB with people with AD and with people without cognitive impairment was published after the completion of this systematic review (Del Campo *et al*., 2023). It reported 49 DAP in CSF of people with DLB, when compared to people with AD. The study developed a customised multiplex biomarker panel including six of those DAP (DDC, CRH, MMP-3, ABL1, MMP-10, and THOP1), and validated their differential abundance in CSF of people with DLB, when compared to people with AD (Del Campo *et al*., 2023).

Predicting progression to PDD in people with PD is a clinical priority (Phongpreecha *et al*., 2020), but none of the included studies assessed longitudinal changes in expression levels of reported DAP in people with PDD. Synucleinopathy is likely to contribute more towards PDD than towards AD pathology, and differential abundance of SYUA is often hypothesised to differentiate people with PDD from people with AD. Our meta-analysis confirmed the hypothesis that CSF SYUA levels are significantly lower in people with PDD than in people with AD. Diagnostic biomarker potential of this finding warrants further evaluation. Moreover, our comprehensive meta-analysis confirmed that CSF TAU levels are higher in people with PDD than in people without dementia (Chin *et al*., 2020; Virgilio *et al*., 2022). TAU pathology is common in both PDD and AD (Zhang *et al*., 2023). However, our meta-analyses showed that CSF TAU levels were significantly lower in people with PDD than in people with AD or other dementia. Hence, CSF TAU levels may help differentiating people with PDD from people with AD. Furthermore, we have presented the first meta-analysis of CSF NFL levels in people with PDD. Our meta-analysis showed that CSF NFL levels were significantly higher in people with PDD than in people without dementia. The remaining 34 reported DAP need further research for verifying their differential abundance, and this systematic review revealed the need for more research on blood-based DAP in people with PDD.

Our functional enrichment analyses help advancing our understanding of molecular pathology of LBD. LBD are protein misfolding neurodegenerative diseases, and Lewy bodies are made of many distinct misfolded proteins (Wakabayashi *et al*., 2012). The reported DAP may contribute to Lewy body formation by impacting protein phosphorylation, protease binding, and serine-type endopeptidase inhibitor activity. The identified DAP may lead to neurodegeneration by impacting apoptosis, regulation of gene expression, and mitochondrial functions. The reported DAP in people with PDD can lead to defective dopamine neurotransmission by interfering dopamine biosynthesis, synaptic plasticity, synaptic vesicle membrane formation, synaptic vesicle maturation, and signal transduction. Further research focusing on these DAP and their functional impact on the enriched molecular pathways may lead to discovery of novel therapeutic targets for LBD.

Chronic neuroinflammation and microglial activation contribute towards AD and PDD pathology (Stamper *et al*., 2008). However, the current evidence indicate absence of chronic neuroinflammation in people with DLB, especially in the later stages of their disease (Santpere *et al*., 2017; Erskine *et al*., 2018; Chowdhury & Rajkumar, 2020; Rajkumar *et al*., 2020). This systematic review has found several neuroinflammation related DAP in DLB and PDD that can influence immune response, inflammatory response, microglial activation, neutrophil chemotaxis, complement cascade, cytokine activity, IL-17 signalling pathway, and NOD-like receptor signalling pathway. Similar to the systematic review of LBD gene expression studies (Chowdhury & Rajkumar, 2020), the direction of differential abundance of reported inflammation related DAP indicates the differences between DLB and PDD pathology. The reported DAP involved in inflammatory pathways, especially interleukins, were significantly less abundant in people with DLB (King *et al*., 2018; Usenko *et al*., 2020). A comprehensive summary of prior mechanistic studies that investigated functional implications of the reported DAP is beyond the scope of this systematic review. However, similar to prior transcriptomic studies that have reported statistically significant downregulation of several pro-inflammatory genes including *IL1B*, *IL2*, *IL6*, *CXCL2*, *CXCL3*, *CXCL8*, *CXCL10*, and *CXCL11* (Santpere *et al*., 2017; Rajkumar *et al*., 2020), 23 neuroinflammation related (GO:0006954; Supplementary information-9) proteins including Interleukin-1 beta, Interleukin-6, Interleukin-8, Interleukin-22, tumour necrosis factor, Complement C3, Complement C4-B, Complement C5, Allograft inflammatory factor 1, and Macrophage migration inhibitory factor were reportedly differentially abundant in people with DLB. Optimal microglial activation and proinflammatory protein expression are essential for neuronal survival and synaptic plasticity (Chen *et al*., 2014). Microglial dysfunction and immunosenescence related changes in the levels of proinflammatory proteins may impair synaptic plasticity and may lead to neurodegeneration in DLB (Rajkumar *et al*., 2020, Chowdhury & Rajkumar, 2020).

The reported DAP in DLB and PDD were enriched among the proteins involved in extracellular region and extracellular space. The DAP in people with DLB were enriched among the proteins involved in extracellular exosomes. CSF small extracellular vesicles (SEV; exosomes) from people with LBD can transmit α-synuclein oligomerisation in vitro (Stuendl *et al*., 2016). SEV can cross blood brain barrier (Schiera *et al*., 2015), and they transport RNA and proteins between brain and blood circulation. Diagnostic biomarker and therapeutic drug delivery potential of SEV in various neurodegenerative disorders are increasingly recognised (Mustapic *et al*., 2017; Isik *et al*., 2024). Our findings highlight the need for further research focusing on blood-based SEV proteins that may facilitate the discovery of novel blood-based biomarkers for LBD.

On the basis of the findings of this systematic review, we suggest the following future research directives: (i) it is high time to plan multi-centre large clinical studies for assessing the clinical utility of CSF SYUA and TAU levels as diagnostic biomarkers for DLB; (ii) more studies are needed for evaluating the diagnostic biomarker potential of various p-TAU levels in CSF and blood of people with DLB; (iii) the current evidence does not support planning further research on CSF NFL levels for differentiating people with DLB from people with AD or other dementia; (iv) there is need for planning longitudinal studies investigating changes in CSF TAU levels for facilitating early diagnosis of PDD in people with PD; (v) future DLB biomarker studies focusing on oligomeric SYUA are warranted; (vi) future DLB and PDD proteomic and targeted protein assay studies should prioritise investigating plasma because of the difficulties in implementing routine lumbar puncture and CSF analysis in mental health settings. Investigating standardised multiplex protein panels in multi-centre clinical cohorts may set the stage for improving clinical diagnosis of DLB and PDD in old age psychiatry and Neurology clinical settings; (vii) more proteomic studies investigating post-mortem DLB and PDD brains are needed for improving our understanding of their molecular biology and for facilitating the discovery of novel therapeutic targets; (viii) future studies investigating blood-based DAP in LBD should focus on plasma small extracellular vesicles; (ix) future studies on this important topic should not fail to consider power estimation, selection bias and multiple testing corrections; and (x) there is need for achieving consensus on uniform use of protein nomenclature in future studies reporting DAP in LBD.

## Supporting information

Farr et al. supplementary material 1 Farr et al. supplementary material

Farr et al. supplementary material 2 Farr et al. supplementary material

Farr et al. supplementary material 3 Farr et al. supplementary material

Farr et al. supplementary material 4 Farr et al. supplementary material

Farr et al. supplementary material 5 Farr et al. supplementary material

Farr et al. supplementary material 6 Farr et al. supplementary material

Farr et al. supplementary material 7 Farr et al. supplementary material

Farr et al. supplementary material 8 Farr et al. supplementary material

Farr et al. supplementary material 9 Farr et al. supplementary material

Farr et al. supplementary material 10 Farr et al. supplementary material

## Data Availability

The data that support the findings of this systematic review are available from the corresponding author upon reasonable request.

## Supplementary material

The supplementary material for this article can be found at https://doi.org/10.1017/neu.2025.15.
