# Supplementary material for: Systematic review of differentially abundant proteins in people with Lewy body dementia
Source: Acta Neuropsychiatr. 2025 Mar 27;37:e59. doi: 10.1017/neu.2025.15 (PMC13130301; doi:10.1017/neu.2025.15)
Supplement: Farr et al. supplementary material 3 — Farr et al. supplementary material [file S0924270825000158sup003.docx]

**Supplementary material-2: A report detailing the search strategies**

**for this systematic review**

**Database Searching**

| Name of Database | Platform | Date searched | Number of results |
| --- | --- | --- | --- |
| Medline | Ovid MEDLINE(R) ALL <1946 to 15^th^ of February 2023> | 16-02-2023 | 1,906 |
| Embase | Ovid Embase <1974 to 2023 16^th^ of February 2023> | 16-02-2023 | 4,642 |
| PsycINFO | Ovid PsycINFO <1806 to 16^th^ of February 2023> | 16-02-2023 | 484 |
| Web of Science Core Collection | Clarivate Analytics | 16-02-2023 | 1,494 |
| Scopus | Elsevier |  | 2,150 |
|  | | | |
| Total number of results retrieved | | | **10,676** |
|  | | | |
| Duplicates found | | | 4,996 |
| Animal studies excluded (that were not captured by the database filters) | | | 157 |
| Article types removed (editorials, letters, commentaries, replies to the author) | | | 294 |
| Database results to review for inclusion | | | **5,229** |

**Website Searching**

| Name of website | Link | Date searched | Number of new results |
| --- | --- | --- | --- |
| TRIP (Turning Research Into Practice) | <https://www.tripdatabase.com/> | 23-02-2023 | 329 |
| National Grey Literature Collection | <https://allcatsrgrey.org.uk/wp/> | 23-02-2023 | 1 |
| Website results to review for inclusion | | | 330 |

**Search Results Total**

| Search | Number of Results |
| --- | --- |
| Database search | 5,229 |
| Website search | 330 |
|  | |
| Total number of results to screen | **5,559** |

**Search History for Databases for published literature**

**Platform and database**: Ovid MEDLINE® ALL <1946 to February 15, 2023>

**Date searched**: 16^th^ of February 2023

|  | Search term(s) | Result(s) |
| --- | --- | --- |
| 1 | "differential* abundant protein*".tw,kw,kf. | 605 |
| 2 | exp Peptides/ | 2890823 |
| 3 | (peptide* or polypeptide*).tw,kw,kf. | 665245 |
| 4 | exp Biomarkers/ | 832260 |
| 5 | (biomarker* or "biological marker*" or "biological indicator*").tw,kw,kf. | 353852 |
| 6 | exp Proteomics/ | 61693 |
| 7 | proteomic*.tw,kw,kf. | 94806 |
| 8 | exp Blotting, Western/ | 163501 |
| 9 | ("western blot*" or "western immunoblot*").tw,kw,kf. | 272916 |
| 10 | exp Immunohistochemistry/ | 611032 |
| 11 | (immunocytochemistry or "immunogold techni*" or "immunogold-silver techni*" or immunohistochemistry or immunohistocytochemistry or "immunolabeling techni*").tw,kw,kf. | 244632 |
| 12 | exp Mass Spectrometry/ | 268091 |
| 13 | ("mass spectrometry" or "mass spectroscopy" or "mass spectrum analys*" or LC-MS).tw,kw,kf. | 305035 |
| 14 | exp Immunoassay/ | 497318 |
| 15 | (immunoassay* or "immunochromatographic assay*").tw,kw,kf. | 75017 |
| 16 | (SIMOA or ELISA or "enzyme-linked immunosorbent assay*").tw,kw,kf. | 237487 |
| 17 | or/1-16 | 4938798 |
| 18 | exp Lewy Body Disease/ | 3860 |
| 19 | (("lewy body" or "lewy bodies") adj2 (dementia or "neurocognitive disorder*")).tw,kw,kf. | 5725 |
| 20 | (parkinson* adj1 (dementia* or "neurocognitive disorder*")).tw,kw,kf. | 2225 |
| 21 | or/18-20 | 8295 |
| 22 | 17 and 21 | 2060 |
| 23 | Animals/ not Humans/ | 4926082 |
| 24 | 22 not 23 | 1971 |
| 25 | limit 24 to english language | 1906 |

**Platform and database**: Ovid Embase <1974 to 2023 February 15>

**Date searched**: 16^th^ of February 2023

|  | Search term(s) | Result(s) |
| --- | --- | --- |
| 1 | "differential* abundant protein*".tw,kw,kf. | 662 |
| 2 | exp peptide/ | 354516 |
| 3 | (peptide* or polypeptide*).tw,kw,kf. | 784703 |
| 4 | biological marker/ | 372578 |
| 5 | (biomarker* or "biological marker*" or "biological indicator*").tw,kw,kf. | 535454 |
| 6 | exp proteomics/ | 111746 |
| 7 | proteomic*.tw,kw,kf. | 123160 |
| 8 | Western blotting/ | 433827 |
| 9 | ("western blot*" or "western immunoblot*").tw,kw,kf. | 392021 |
| 10 | exp immunohistochemistry/ | 700623 |
| 11 | (immunocytochemistry or "immunogold techni*" or "immunogold-silver techni*" or immunohistochemistry or immunohistocytochemistry or "immunolabeling techni*").tw,kw,kf. | 378605 |
| 12 | exp mass spectrometry/ | 530428 |
| 13 | ("mass spectrometry" or "mass spectroscopy" or "mass spectrum analys*" or LC-MS).tw,kw,kf. | 375910 |
| 14 | exp immunoassay/ | 644985 |
| 15 | (immunoassay* or "immunochromatographic assay*").tw,kw,kf. | 100990 |
| 16 | (SIMOA or ELISA or "enzyme-linked immunosorbent assay*").tw,kw,kf. | 354424 |
| 17 | or/1-16 | 3642219 |
| 18 | diffuse Lewy body disease/ | 10120 |
| 19 | (("lewy body" or "lewy bodies") adj2 (dementia or "neurocognitive disorder*")).tw,kw,kf. | 9301 |
| 20 | (parkinson* adj1 (dementia* or "neurocognitive disorder*")).tw,kw,kf. | 3882 |
| 21 | or/18-20 | 15702 |
| 22 | 17 and 21 | 5195 |
| 23 | exp Animals/ or exp Invertebrate/ or Animal Experiment/ or Animal Model/ or Animal Tissue/ or Animal Cell/ or Nonhuman/ | 30132424 |
| 24 | Human/ or Normal Human/ or Human Cell/ | 23359461 |
| 25 | 23 and 24 | 23291563 |
| 26 | 23 not 25 | 6840861 |
| 27 | 22 not 26 | 4767 |
| 28 | limit 27 to english language | 4642 |

**Platform and database**: Ovid APA PsycINFO <1806 to February Week 1 2023>

**Date searched**: 16^th^ of February 2023

|  | Search term(s) | Result(s) |
| --- | --- | --- |
| 1 | "differential* abundant protein*".tw. | 5 |
| 2 | exp Peptides/ | 42045 |
| 3 | (peptide* or polypeptide*).tw. | 18316 |
| 4 | Biological Markers/ | 15523 |
| 5 | (biomarker* or "biological marker*" or "biological indicator*").tw. | 25850 |
| 6 | Proteomics/ | 705 |
| 7 | proteomic*.tw. | 1868 |
| 8 | ("western blot*" or "western immunoblot*").tw. | 7782 |
| 9 | Immunocytochemistry/ | 1944 |
| 10 | (immunocytochemistry or "immunogold techni*" or "immunogold-silver techni*" or immunohistochemistry or immunohistocytochemistry or "immunolabeling techni*").tw. | 9703 |
| 11 | ("mass spectrometry" or "mass spectroscopy" or "mass spectrum analys*" or LC-MS).tw. | 3272 |
| 12 | (immunoassay* or "immunochromatrographic assay*").tw. | 1396 |
| 13 | (SIMOA or ELISA or "enzyme-linked immunosorbent assay*").tw. | 4863 |
| 14 | or/1-13 | 98324 |
| 15 | Dementia with Lewy Bodies/ | 2158 |
| 16 | (("lewy body" or "lewy bodies") adj2 (dementia or "neurocognitive disorder*")).tw. | 2967 |
| 17 | (parkinson* adj1 (dementia or "neurocognitive disorder*")).tw. | 396 |
| 18 | or/15-17 | 3635 |
| 19 | 13 and 17 | 525 |
| 20 | exp Animals/ not (exp Human Males/ or exp Human Females/) | 364281 |
| 21 | 19 not 20 | 505 |
| 22 | limit 21 to english language | 493 |

**Platform and database**: Clarivate Analytics Web of Science Core Collection

**Editions**: All

**Date searched**: 16^th^ of February 2023

|  | Search term(s) | Result(s) |
| --- | --- | --- |
| 1 | "differential* abundant protein*" (Topic) | 651 |
| 2 | peptide* or polypeptide* (Topic) | 851,528 |
| 3 | biomarker* or "biological marker*" or "biological indicator*" (Topic) | 459,066 |
| 4 | proteomic* (Topic) | 127,597 |
| 5 | "western blot*" or "western immunoblot*" (Topic) | 256,105 |
| 6 | immunocytochemistry or "immunogold techni*" or "immunogold-silver techni*" or immunohistochemistry or immunohistocytochemistry or "immunolabeling techni*" (Topic) | 264,982 |
| 7 | "mass spectrometry" or "mass spectroscopy" or "mass spectrum analys*" or LC-MS (Topic) | 555,591 |
| 8 | immunoassay* or "immunochromatrographic assay*" (Topic) | 98,496 |
| 9 | SIMOA or ELISA or "enzyme-linked immunosorbent assay*" (Topic) | 233,463 |
| 10 | 1 OR 2 OR 3 OR 4 OR 5 OR 6 OR 7 OR 8 OR 9 | 2,458,203 |
| 11 | ("lewy body" or "lewy bodies") NEAR/2 (dementia or "neurocognitive disorder*") (Topic) | 6,658 |
| 12 | parkinson* NEAR/1 (dementia or "neurocognitive disorder*") (Topic) | 3,704 |
| 13 | 11 OR 12 | 9,820 |
| 14 | 10 AND 13 | 1,521 |
| 15 | 10 AND 13 Refined By: Languages: English | 1,494 |

**Platform and database**: Elsevier Scopus

**Date searched**: 16^th^ of February 2023

|  | Search term(s) | Result(s) |
| --- | --- | --- |
| 1 | TITLE-ABS-KEY ( "differential* abundant protein*" ) | 663 |
| 2 | TITLE-ABS-KEY ( peptide* OR polypeptide* ) | 1,125,866 |
| 3 | TITLE-ABS-KEY ( biomarker* OR "biological marker*" OR "biological indicator*" ) | 709,599 |
| 4 | TITLE-ABS-KEY ( proteomic* ) | 146,208 |
| 5 | TITLE-ABS-KEY ( "western blot*" OR "western immunoblot*" ) | 454,427 |
| 6 | TITLE-ABS-KEY ( immunocytochemistry OR "immunogold techni*" OR "immunogold-silver techni*" OR immunohistochemistry OR immunohistocytochemistry OR "immunolabeling techni*" ) | 767,565 |
| 7 | TITLE-ABS-KEY ( "mass spectrometry" OR "mass spectroscopy" OR "mass spectrum analys*" OR lc-ms ) | 759,967 |
| 8 | TITLE-ABS-KEY ( immunoassay* OR "immunochromatrographic assay*" ) | 169,774 |
| 9 | TITLE-ABS-KEY ( simoa OR elisa OR "enzyme-linked immunosorbent assay*" ) | 450,754 |
| 10 | 1 OR 2 OR 3 OR 4 OR 5 OR 6 OR 7 OR 8 OR 9 | 3,791,639 |
| 11 | TITLE-ABS-KEY ( ( "lewy body" OR "lewy bodies" ) W/2 ( dementia OR "neurocognitive disorder*" ) ) | 6,649 |
| 12 | TITLE-ABS-KEY ( parkinson* W/1 ( dementia OR "neurocognitive disorder*" ) ) | 4,526 |
| 13 | 11 OR 12 | 10,064 |
| 14 | 10 AND 13 | 2,235 |
| 15 | (10 AND 13) AND (LIMIT-TO ( LANGUAGE , "English" ) ) | 2,150 |

**Website**: TRIP (Turning Research Into Practice)

**Date searched**: 23^rd^ of February 2023

**Terms searched:**

"differentially abundant protein" AND "lewy body"

Peptide AND "lewy body"

Biomarker AND "lewy body"

Proteomic AND "lewy body"

"western blot" AND "lewy body"

Immunohistochemistry AND "lewy body"

"mass spectrometry" AND "lewy body"

Immunoassay AND "lewy body"

"differentially abundant protein" AND "Parkinson dementia"

Peptide AND "Parkinson dementia"

Biomarker AND "Parkinson dementia"

Proteomic AND "Parkinson dementia"

"western blot" AND "Parkinson dementia"

Immunohistochemistry AND "Parkinson dementia"

"mass spectrometry" AND "Parkinson dementia"

Immunoassay AND "Parkinson dementia"

**Website**: National Grey Literature Collection

**Date searched**: 23-02-2023

**Terms searched:**

"differentially abundant protein"

Peptide

Biomarker

Proteomic

"western blot"

Immunohistochemistry

"mass spectrometry"

Immunoassay

**Unique results found**: 1

Biomarker AND "lewy body": [A review of diagnostic imaging for dementia in Wessex](https://allcatsrgrey.org.uk/wp/download/public_health/mental_health/dementia/CS50620-Wessex-review-of-diagnostic-imaging-for-dementia-web.pdf) (NHS Wessex Clinical Networks, January 2020).
