# Supplementary material for: Systematic review of differentially abundant proteins in people with Lewy body dementia
Source: Acta Neuropsychiatr. 2025 Mar 27;37:e59. doi: 10.1017/neu.2025.15 (PMC13130301; doi:10.1017/neu.2025.15)
Supplement: Farr et al. supplementary material 4 — Farr et al. supplementary material [file S0924270825000158sup004.docx]

**Quality assessment of studies investigating differential abundance of proteins^1^**

^1^ **Adapted from**:

Sohani ZN, Meyre D, De Souza RJ, Joseph PG, Gandhi M, Dennis BB, et al. Assessing the quality of published genetic association studies in meta-analyses: the quality of genetic studies (Q-Genie) tool. BMC Genet. 2015;16:50.

## 1. Rationale for study

Please rate the study on the adequacy of the presented hypothesis and rationale.

When rating the study, please consider the following:

• Was a scientific rationale for chosen proteins presented to avoid selective reporting of positive results?

If this is a proteomic study, where a hypothesis-free approach is taken, a rationale for selecting this design should be presented.

| 1 |  | 2 |  | 3 |  | 4 |  | 5 |  | 6 |  | 7 |
| --- | --- | --- | --- | --- | --- | --- | --- | --- | --- | --- | --- | --- |

Poor Good Very Good Excellent

**2. Selection and definition of outcome of interest.** The outcome can be cases/disease status or a quantitative trait.

Please rate the study on the classification of the outcome (e.g. disease status or quantitative trait).

When rating the study, please consider the following:

- Were the cases appropriately defined?

Outcome definitions will vary from independent adjudication or reliable data from clinical records or laboratory measures (strong) to self-report (moderate) to no-description (poor) `

- Were participants appropriately sampled?

Participants should be sampled in a way to avoid selection bias as appropriate to the study objectives (e.g. such as selecting the most sick cases if the objective is not to enrich cases). Included participants should reflect the entire population of interest.

- Were the case/outcome assessors blinded to the protein expression status?
- If applicable, was follow-up length appropriate for outcome to occur and was the attrition rate acceptable?

| 1 |  | 2 |  | 3 |  | 4 |  | 5 |  | 6 |  | 7 |
| --- | --- | --- | --- | --- | --- | --- | --- | --- | --- | --- | --- | --- |

Poor Good Very Good Excellent

## 3. Selection and comparability of comparison groups (if applicable)

Please rate the study on appropriateness of comparison groups (e.g. control groups).

When rating the study, please consider the following:

- Were the controls appropriately defined?
- Were the controls sampled in a way to minimize selection bias?
- Was a detailed description of selection procedure (i.e. eligibility criteria, sources and methods of ascertainment, methods of matching if applicable) outlined or referenced?
- Were the assessors of control status blinded to the protein expression status?

| 1 |  | 2 |  | 3 |  | 4 |  | 5 |  | 6 |  | 7 |
| --- | --- | --- | --- | --- | --- | --- | --- | --- | --- | --- | --- | --- |

Poor Good Very Good Excellent

## 4. Technical classification of the exposure

Please rate the study on the technical assessment of protein expression.

When rating the study, please consider the following:

- Was the source (e.g. CSF fluid) and method of storage for the sample appropriate?
- Were the methods of measuring protein expression similar for comparison groups (if applicable)?
- Was the assessment of protein expression appropriate?
- If applicable, did the authors check for potential outlier samples to assess quality of protein expression assessment?

| 1 |  | 2 |  | 3 |  | 4 |  | 5 |  | 6 |  | 7 |
| --- | --- | --- | --- | --- | --- | --- | --- | --- | --- | --- | --- | --- |

Poor Good Very Good Excellent

## 5. Non-technical classification of the exposure

Please rate the study on the non-technical assessment of protein expression.

When rating the study, please consider the following:

- Did a blinded assessor conduct the protein expression assessment?
- Was protein expression assessment conducted in all the participants from the study simultaneously or in smaller batches? If so, were methods across batches same?
- If applicable, were samples randomized prior to protein expression assessment (e.g. not all controls on one plate and cases on another)?
- Were there appropriate negative controls?

| 1 |  | 2 |  | 3 |  | 4 |  | 5 |  | 6 |  | 7 |
| --- | --- | --- | --- | --- | --- | --- | --- | --- | --- | --- | --- | --- |

Poor Good Very Good Excellent

## 6. Other sources of bias

Please rate the study on the disclosure and discussion of sources of bias.

In addition to selection and classification bias previously discussed, many other potential sources of bias exist (e.g. time-lag bias, attrition bias, et cetera). Please consider whether all sources of bias were disclosed and their effect on the results discussed.

| 1 |  | 2 |  | 3 |  | 4 |  | 5 |  | 6 |  | 7 |
| --- | --- | --- | --- | --- | --- | --- | --- | --- | --- | --- | --- | --- |

Poor Good Very Good Excellent

## 7. Sample size and power

Please rate whether the study was adequately powered.

- Was the sample size appropriate?
- Was an a priori power analysis conducted?

| 1 |  | 2 |  | 3 |  | 4 |  | 5 |  | 6 |  | 7 |
| --- | --- | --- | --- | --- | --- | --- | --- | --- | --- | --- | --- | --- |

Poor Good Very Good Excellent

## 8. A priori planning of analyses

Please rate the study on the planned analyses.

- Was the analysis plan appropriate and sufficiently described?
- Was selective and/or inappropriate reporting avoided (i.e. all results from tests conducted were reported)? Authors should identify where additional results can be found if not included in the primary paper (e.g. supplementary tables).
- Were the tested subgroups, interactions, and sensitivity analyses described and reported?
- Was the statistical software used identified?

| 1 |  | 2 |  | 3 |  | 4 |  | 5 |  | 6 |  | 7 |
| --- | --- | --- | --- | --- | --- | --- | --- | --- | --- | --- | --- | --- |

Poor Good Very Good Excellent

**9. Statistical methods and control for confounding**

Please rate the study on statistical methods.

- Were important confounders appropriately controlled?
- Were missing data for samples and protein expression appropriately handled?
- Were the results adjusted for multiple testing to avoid false positive results? Please note this is particularly important in analyses of large datasets.

| 1 |  | 2 |  | 3 |  | 4 |  | 5 |  | 6 |  | 7 |
| --- | --- | --- | --- | --- | --- | --- | --- | --- | --- | --- | --- | --- |

Poor Good Very Good Excellent

## 10. Testing of assumptions and inferences for protein expression analyses

Please rate the study on the description and test of all assumptions and inferences Including,

- Was specificity of antibodies, if applicable, tested?
- Did standard curve show a linear relationship between the signal and analyte concentration?

| 1 |  | 2 |  | 3 |  | 4 |  | 5 |  | 6 |  | 7 |
| --- | --- | --- | --- | --- | --- | --- | --- | --- | --- | --- | --- | --- |

Poor Good Very Good Excellent

## 11. Appropriateness of inferences drawn from results

Please rate the study on whether conclusions drawn by the authors were supported by the results and appropriate methods.

| 1 |  | 2 |  | 3 |  | 4 |  | 5 |  | 6 |  | 7 |
| --- | --- | --- | --- | --- | --- | --- | --- | --- | --- | --- | --- | --- |

Poor Good Very Good Excellent

# Scoring

**Please add the total score from each question.**

**For studies with control groups:** Scores ≤35 indicate poor quality studies, >35 and ≤45 indicate studies of moderate quality, and >45 indicate good quality studies.

**For studies without control groups:** Scores ≤32 indicate poor quality studies, >32 and ≤40 indicate studies of moderate quality, and >40 indicate good quality studies.
