# Supplementary material for: Systematic review of differentially abundant proteins in people with Lewy body dementia
Source: Acta Neuropsychiatr. 2025 Mar 27;37:e59. doi: 10.1017/neu.2025.15 (PMC13130301; doi:10.1017/neu.2025.15)
Supplement: Farr et al. supplementary material 5 — Farr et al. supplementary material [file S0924270825000158sup005.docx]

**Supplementary material-4: Full List of Included Studies**

1. **Abdi F, Quinn JF, Jankovic J, McIntosh M, Leverenz JB, Peskind E, Nixon R, Nutt J, Chung K, Zabetian C, Samii A, Lin M, Hattan S, Pan C, Wang Y, Jin J, Zhu D, Li GJ, Liu Y, Waichunas D, Montine TJ and Zhang J** (2006). Detection of biomarkers with a multiplex quantitative proteomic platform in cerebrospinal fluid of patients with neurodegenerative disorders. Journal of Alzheimer’s Disease **9(3)**, 293–348.
2. **Abu-Rumeileh S, Capellari S, Stanzani-Maserati M, Polischi B, Martinelli P, Caroppo P, Ladogana A and Parchi P** (2018). The CSF neurofilament light signature in rapidly progressive neurodegenerative dementias. Alzheimer’s Research and Therapy **10(1)**, 3.
3. **Abu-Rumeileh S, Giannini G, Polischi B, Albini-Riccioli L, Milletti D, Oppi F, Stanzani-Maserati M, Capellari S, Mantovani P, Palandri G, Cortelli P, Cevoli S and Parchi P** (2019). Revisiting the Cerebrospinal Fluid Biomarker Profile in Idiopathic Normal Pressure Hydrocephalus: The Bologna Pro-Hydro Study. Journal of Alzheimer’s Disease **68(2)**, 723–733.
4. **Aerts MB, Esselink RAJ, Abdo WF, Bloem BR and Verbeek MM** (2012). CSF α-synuclein does not differentiate between parkinsonian disorders. Neurobiology of Aging **33(2)**, 430.e1-430.e3.
5. **Aksenova MV, Aksenov MY, Payne RM, Trojanowski JQ, Schmidt ML, Carney JM, Butterfield DA and Markesbery WR** (1999). Oxidation of cytosolic proteins and expression of creatine kinase BB in frontal lobe in different neurodegenerative disorders. Dementia and Geriatric Cognitive Disorders **10(2)**, 158–165.
6. **Alcolea D, Delaby C, Muñoz L, Torres S, Estellés T, Zhu N, Barroeta I, Carmona-Iragui M, Illán-Gala I, Santos-Santos MÁ, Altuna M, Sala I, Sánchez-Saudinós MB, Videla L, Valldeneu S, Subirana A, Pegueroles J, Hirtz C, Vialaret J, Lehmann S, Karikari TK, Ashton NJ, Blennow K, Zetterberg H, Belbin O, Blesa R, Clarimón J, Fortea J, and Lleó, A** (2021). Use of plasma biomarkers for AT(N) classification of neurodegenerative dementias. Journal of Neurology, Neurosurgery and Psychiatry **92(11)**, 1206–1214.
7. **Amin J, Boche D, Clough Z, Teeling J, Williams A, Gao Y, Chudley L, Lau L, Smith F, Harris S and Holmes C** (2020). Peripheral immunophenotype in dementia with Lewy bodies and Alzheimer’s disease: An observational clinical study. Journal of Neurology, Neurosurgery and Psychiatry **91(11)**, 1219–1226.
8. **Amin J, Holmes C, Dorey RB, Tommasino E, Casal YR, Williams DM, Dupuy C, Nicoll JAR and Boche D** (2020). Neuroinflammation in dementia with Lewy bodies: A human post-mortem study. Translational Psychiatry **10(1)**, 267.
9. **Andreasen N, Minthon L, Davidsson P, Vanmechelen E, Vanderstichele H, Winblad B and Blennow K** (2001). Evaluation of CSF-tau and CSF-A242 as Diagnostic Markers for Alzheimer Disease in Clinical Practice. Archives of Neurology **58(3)**, 373-379.
10. **Ashton NJ, Janelidze S, Al Khleifat A, Leuzy A, Van Der Ende EL, Karikari TK, Benedet AL, Pascoal TA, Lleó A, Parnetti L, Galimberti D, Bonanni L, Pilotto A, Padovani A, Lycke J, Novakova L, Axelsson M, Velayudhan L, Rabinovici GD, Miller B, Pariante C, Nikkheslat N, Resnick SM, Thambisetty M, Schöll M, Fernández-Eulate G, Gil-Bea FJ, de Munain AL, Al-Chalabi A, Rosa-Neto P, Strydom A, Svenningsson P, Stomrud E, Santillo A, Aarsland D, van Swieten JC, Palmqvist S, Zetterberg H, Blennow K, Hye A, and Hansson O** (2021). A multicentre validation study of the diagnostic value of plasma neurofilament light. Nature Communications **12(1)**, 3400.
11. **Ayton S, Hall S, Janelidze S, Kalinowski P, Palmqvist S, Belaidi AA, Roberts B, Roberts A, Stomrud E, Bush AI and Hansson O** (2022). The Neuroinflammatory Acute Phase Response in Parkinsonian‐Related Disorders. Movement Disorders **37(5)**, 993–1003.
12. **Baek JH, Whitfield D, Howlett D, Francis P, Bereczki E, Ballard C, Hortobágyi T, Attems J and Aarsland D** (2016). Unfolded protein response is activated in Lewy body dementias: UPR activation in Lewy body dementias. Neuropathology and Applied Neurobiology **42(4)**, 352–365.
13. **Ballard C, Jones EL, Londos E, Minthon L, Francis P and Aarsland D** (2010). α-synuclein antibodies recognize a protein present at lower levels in the CSF of patients with dementia with Lewy bodies. International Psychogeriatrics **22(2)**, 321–327.
14. **Baumann CR, Dauvilliers Y, Mignot E and Bassetti CL** (2004). Normal CSF Hypocretin-1 (Orexin A) Levels in Dementia with Lewy Bodies Associated with Excessive Daytime Sleepiness. European Neurology **52(2)**, 73–76.
15. **Bawaskar HS, Bawaskar PH and Bawaskar PH** (2015). RBC acetyl cholinesterase: A poor man’s early diagnostic biomarker for familial alzheimer’s and Parkinson’s disease dementia. Journal of Neurosciences in Rural Practice **6(01)**, 33–38.
16. **Bereczki E, Branca RM, Francis PT, Pereira JB, Baek JH, Hortobágyi T, Winblad B, Ballard C, Lehtiö J and Aarsland D** (2018). Synaptic markers of cognitive decline in neurodegenerative diseases: A proteomic approach. Brain **141(2)**, 582–595.
17. **Bereczki E, Francis PT, Howlett D, Pereira JB, Höglund K, Bogstedt A, Cedazo‐Minguez A, Baek J, Hortobágyi T, Attems J, Ballard C and Aarsland D** (2016). Synaptic proteins predict cognitive decline in Alzheimer’s disease and Lewy body dementia. Alzheimer’s and Dementia **12(11)**, 1149–1158.
18. **Bibl M, Esselmann H, Lewczuk P, Trenkwalder C, Otto M, Kornhuber J, Wiltfang J and Mollenhauer B** (2010). Combined Analysis of CSF Tau, Aβ42, Aβ1-42% and Aβ1-40% in Alzheimer's Disease, Dementia with Lewy Bodies and Parkinson's Disease Dementia. International Journal of Alzheimer’s Disease **2010**, 761571.
19. **Bibl M, Mollenhauer B, Esselmann H, Lewczuk P, Trenkwalder C, Brechlin P, Rüther E, Kornhuber J, Otto M and Wiltfang J** (2006). CSF diagnosis of Alzheimer’s disease and dementia with Lewy bodies. Journal of Neural Transmission **113(11)**, 1771–1778.
20. **Björkqvist M, Ohlsson M, Minthon L and Hansson O** (2012). Evaluation of a Previously Suggested Plasma Biomarker Panel to Identify Alzheimer’s Disease. PLoS ONE **7(1)**, e29868.
21. **Boiten W, Van Steenoven I, Xiao MF, Worley P, Noli B, Cocco C, Ferri GL, Lemstra A and Teunissen C** (2020). Pathologically Decreased CSF Levels of Synaptic Marker NPTX2 in DLB Are Correlated with Levels of Alpha-Synuclein and VGF. Cells **10(1)**, 38.
22. **Bonomi CG, De Lucia V, Mascolo AP, Assogna M, Motta C, Scaricamazza E, Sallustio F, Mercuri NB, Koch G and Martorana A** (2021). Brain energy metabolism and neurodegeneration: Hints from CSF lactate levels in dementias. Neurobiology of Aging **105**, 333–339.
23. **Borroni B, Malinverno M, Gardoni F, Alberici A, Parnetti L, Premi E, Bonuccelli U, Grassi M, Perani D, Calabresi P, Di Luca M and Padovani A** (2008). Tau forms in CSF as a reliable biomarker for progressive supranuclear palsy. *Neurology*, **71(22)**, 1796–1803.
24. **Boström F, Hansson O, Blennow K, Gerhardsson L, Lundh T, Minthon L, Zetterberg H and Londos E** (2009). Cerebrospinal Fluid Total Tau Is Associated with Shorter Survival in Dementia with Lewy Bodies. Dementia and Geriatric Cognitive Disorders **28(4)**, 314–319.
25. **Bougea A, Stefanis L, Emmanouilidou E, Vekrelis K and Kapaki E** (2020). High discriminatory ability of peripheral and CFSF biomarkers in Lewy body diseases. Journal of Neural Transmission **127(3)**, 311–322.
26. **Bougea A, Stefanis L, Paraskevas GP, Emmanouilidou E, Efthymiopoulou E, Vekrelis K and Kapaki E** (2018). Neuropsychiatric symptoms and α-Synuclein profile of patients with Parkinson’s disease dementia, dementia with Lewy bodies and Alzheimer’s disease. Journal of Neurology **265(10)**, 2295–2301.
27. **Bousiges O, Bombois S, Schraen S, Wallon D, Quillard MM, Gabelle A, Lehmann S, Paquet C, Amar-Bouaziz E, Magnin E, Miguet-Alfonsi C, Delbeuck X, Lavaux T, Anthony P, Philippi N and Blanc F** (2018). Cerebrospinal fluid Alzheimer biomarkers can be useful for discriminating dementia with Lewy bodies from Alzheimer’s disease at the prodromal stage. Journal of Neurology, Neurosurgery and Psychiatry **89(5)**, 467–475.
28. **Bousiges O, Philippi N, Lavaux T, Perret-Liaudet A, Lachmann I, Schaeffer-Agalède C, Anthony P, Botzung A, Rauch L, Jung B, De Sousa PL, Demuynck C, Martin-Hunyadi C, Cretin B and Blanc F** (2020). Differential diagnostic value of total alpha-synuclein assay in the cerebrospinal fluid between Alzheimer’s disease and dementia with Lewy bodies from the prodromal stage. Alzheimer’s Research and Therapy **12(1)**, 120.
29. **Brockmann K, Quadalti C, Lerche S, Rossi M, Wurster I, Baiardi S, Roeben B, Mammana A, Zimmermann M, Hauser AK, Deuschle C, Schulte C, Waniek K, Lachmann I, Sjödin S, Brinkmalm A, Blennow K, Zetterberg H, Gasser T and Parchi P** (2021). Association between CSF alpha-synuclein seeding activity and genetic status in Parkinson’s disease and dementia with Lewy bodies. Acta Neuropathologica Communications **9(1)**, 175.
30. **Brooks D and Halliday GM** (2009). Intralaminar nuclei of the thalamus in Lewy body diseases. Brain Research Bulletin **78(2–3**), 97–104.
31. **Bruggink KA, Kuiperij HB, Gloerich J, Otte-Höller I, Rozemuller AJM, Claassen JAHR, Küsters B and Verbeek MM** (2015). Dickkopf-related protein 3 is a potential Aβ-associated protein in Alzheimer’s Disease. Journal of Neurochemistry **134(6)**, 1152–1162.
32. **Brunnström H, Rawshani N, Zetterberg H, Blennow K, Minthon L, Passant U and Englund E** (2010). Cerebrospinal fluid biomarker results in relation to neuropathological dementia diagnoses. Alzheimer’s and Dementia **6(2)**, 104–109.
33. **Bruun M, Rhodius‐Meester HFM, Koikkalainen J, Baroni M, Gjerum L, Lemstra AW, Barkhof F, Remes AM, Urhemaa T, Tolonen A, Rueckert D, Gils M, Frederiksen KS, Waldemar G, Scheltens P, Mecocci P, Soininen H, Lötjönen J, Hasselbalch SG and Flier WM** (2018). Evaluating combinations of diagnostic tests to discriminate different dementia types. Alzheimer’s and Dementia: Diagnosis, Assessment and Disease Monitoring **10(1)**, 509–518.
34. **Buongiorno M, Antonelli F, Compta Y, Fernandez Y, Pavia J, Lomeña F, Ríos J, Ramírez I, García JR, Soler M, Cámara A, Fernández M, Basora M, Salazar F, Sanchez-Etayo G, Valldeoriola F, Barrio JR and Marti MJ** (2016). Cross-Sectional and Longitudinal Cognitive Correlates of FDDNP PET and CSF Amyloid-β and Tau in Parkinson’s Disease. Journal of Alzheimer’s Disease **55(3)**, 1261–1272.
35. **Canaslan S, Schmitz M, Villar-Piqué A, Maass F, Gmitterová K, Varges D, Lingor P, Llorens F, Hermann P and Zerr I** (2021). Detection of Cerebrospinal Fluid Neurofilament Light Chain as a Marker for Alpha-Synucleinopathies. Frontiers in Aging Neuroscience **13**, 717930.
36. **Chatterjee M, Van Steenoven I, Huisman E, Oosterveld L, Berendse H, Van Der Flier WM, Del Campo M, Lemstra AW, Van De Berg WDJ and Teunissen CE** (2020). Contactin-1 is Reduced in Cerebrospinal Fluid of Parkinson’s Disease Patients and Is Present within Lewy Bodies. Biomolecules **10(8)**, 1177.
37. **Chen CH, Lee BC and Lin CH** (2020). Integrated Plasma and Neuroimaging Biomarkers Associated with Motor and Cognition Severity in Parkinson’s Disease. Journal of Parkinson’s Disease **10(1)**, 77–88.
38. **Chiasserini D, Biscetti L, Eusebi P, Salvadori N, Frattini G, Simoni S, De Roeck N, Tambasco N, Stoops E, Vanderstichele H, Engelborghs S, Mollenhauer B, Calabresi P and Parnetti L** (2017). Differential role of CSF fatty acid binding protein 3, α-synuclein and Alzheimer’s disease core biomarkers in Lewy body disorders and Alzheimer’s dementia. Alzheimer’s Research and Therapy **9(1)**, 52.
39. **Chouliaras L, Thomas A, Malpetti M, Donaghy P, Kane J, Mak E, Savulich G, Prats-Sedano MA, Heslegrave AJ, Zetterberg H, Su L, Rowe JB and O’Brien JT** (2022). Differential levels of plasma biomarkers of neurodegeneration in Lewy body dementia, Alzheimer’s disease, frontotemporal dementia and progressive supranuclear palsy. Journal of Neurology, Neurosurgery and Psychiatry **93(6)**, 651–658.
40. **Clark CM, Xie S, Chittams J, Ewbank D, Peskind E, Galasko D, Morris JC, McKeel DW, Farlow M, Weitlauf SL, Quinn J, Kaye J, Knopman D, Arai H, Doody RS, DeCarli C, Leight S, Lee VMY and Trojanowski JQ** (2003). Cerebrospinal fluid tau and beta-amyloid: How well do these biomarkers reflect autopsy-confirmed dementia diagnoses? Archives of Neurology **60(12)**, 1696–1702.
41. **Colloby SJ, McParland S, O’Brien JT and Attems J** (2012). Neuropathological correlates of dopaminergic imaging in Alzheimer’s disease and Lewy body dementias. Brain **135(9)**, 2798–2808.
42. **Compta Y, Buongiorno M, Bargalló N, Valldeoriola F, Muñoz E, Tolosa E, Ríos J, Cámara A, Fernández M and Martí MJ** (2016). White matter hyperintensities, cerebrospinal amyloid-β and dementia in Parkinson’s disease. Journal of the Neurological Sciences **367**, 284–290.
43. **Compta Y, Ibarretxe-Bilbao N, Pereira JB, Junqué C, Bargalló N, Tolosa E, Valldeoriola F, Muñoz E, Camara A, Buongiorno M and Martí MJ** (2012). Grey matter volume correlates of cerebrospinal markers of Alzheimer-pathology in Parkinson’s disease and related dementia. Parkinsonism and Related Disorders **18(8)**, 941–947.
44. **Compta Y, Martí MJ, Ibarretxe-Bilbao N, Junqué C, Valldeoriola F, Muñoz E, Ezquerra M, Ríos J and Tolosa E** (2009). Cerebrospinal tau, phospho-tau and beta-amyloid and neuropsychological functions in Parkinson’s disease: CSF and Neuropsychological Markers in PD. Movement Disorders **24(15)**, 2203–2210.
45. **Compta Y, Valente T, Saura J, Segura B, Iranzo, Á, Serradell M, Junqué C, Tolosa E, Valldeoriola F, Muñoz E, Santamaria J, Cámara A, Fernández M, Fortea J, Buongiorno M, Molinuevo JL, Bargalló N and Martí MJ** (2015). Correlates of cerebrospinal fluid levels of oligomeric- and total-α-synuclein in premotor, motor and dementia stages of Parkinson’s disease. Journal of Neurology **262(2)**, 294–306.
46. **Daniele S, Baldacci F, Piccarducci R, Palermo G, Giampietri L, Manca ML, Pietrobono D, Frosini D, Nicoletti V, Tognoni G, Giorgi FS, Lo Gerfo A, Petrozzi L, Cavallini C, Franzoni F, Ceravolo R, Siciliano G, Trincavelli ML, Martini C and Bonuccelli U** (2021). α-Synuclein Heteromers in Red Blood Cells of Alzheimer’s Disease and Lewy Body Dementia Patients. Journal of Alzheimer’s Disease **80(2)**, 885–893.
47. **De Jong D, Jansen RWMM, Pijnenburg YAL, Van Geel WJA, Borm GF, Kremer HPH and Verbeek MM** (2007). CSF neurofilament proteins in the differential diagnosis of dementia. Journal of Neurology, Neurosurgery and Psychiatry **78(9)**, 936–938.
48. **De Oliveira FF, Miraldo MC, De Castro-Neto EF, De Almeida SS, Matas SLDA, Bertolucci PHF and Naffah-Mazzacoratti MDG** (2021). Associations of Neuropsychiatric Features with Cerebrospinal Fluid Biomarkers of Amyloidogenesis and Neurodegeneration in Dementia with Lewy Bodies Compared with Alzheimer’s Disease and Cognitively Healthy People. Journal of Alzheimer’s Disease **81(3)**, 1295–1309.
49. **De Riva V, Galloni E, Lealini B, Zarantonello G, Disco C, Dionisio L, Meligrana L, Marcon M and Perini F** (2019). Ratio Aβ1-42:p-Tau: A Possible Diagnostic Tool in Differentiating Dementias. Clinical Laboratory **65**.
50. **Delaby C, Alcolea D, Carmona-Iragui M, Illán-Gala I, Morenas-Rodríguez E, Barroeta I, Altuna M, Estellés T, Santos-Santos M, Turon-Sans J, Muñoz L, Ribosa-Nogué R, Sala-Matavera I, Sánchez-Saudinos B, Subirana A, Videla L, Benejam B, Sirisi S, Lehmann S, Belbin O, Clarimon J, Blesa R, Pagonabarraga J, Rojas-Garcia R, Fortea J,  and Lleó A** (2020). Differential levels of Neurofilament Light protein in cerebrospinal fluid in patients with a wide range of neurodegenerative disorders. Scientific Reports **10(1)**, 9161.
51. **Delaby C, Estellés T, Zhu N, Arranz J, Barroeta I, Carmona-Iragui M, Illán-Gala I, Santos-Santos MÁ, Altuna M, Sala I, Sánchez-Saudinós MB, Videla L, Valldeneu S, Subirana A, Tondo M, Blanco-Vaca F, Lehmann S, Belbin O, Blesa R, Fortea J, Lleó A and Alcolea D** (2022). The Aβ1–42/Aβ1–40 ratio in CSF is more strongly associated to tau markers and clinical progression than Aβ1–42 alone. Alzheimer’s Research and Therapy **14(1)**, 20.
52. **Diaz-Lucena D, Escaramis G, Villar-Piqué A, Hermann P, Schmitz M, Varges D, Santana I, Del Rio JA, Martí E, Ferrer I, Baldeiras I, Zerr I and Llorens F** (2020). A new tetra-plex fluorimetric assay for the quantification of cerebrospinal fluid β-amyloid42, total-tau, phospho-tau and α-synuclein in the differential diagnosis of neurodegenerative dementia. Journal of Neurology **267(9)**, 2567–2581.
53. **Diekämper E, Brix B, Stöcker W, Vielhaber S, Galazky I, Kreissl MC, Genseke P, Düzel E and Körtvelyessy P** (2021). Neurofilament Levels Are Reflecting the Loss of Presynaptic Dopamine Receptors in Movement Disorders. Frontiers in Neuroscience **15**, 690013.
54. **Dieks JK, Gawinecka J, Asif AR, Varges D, Gmitterova K, Streich JH, Dihazi H, Heinemann U and Zerr I** (2013). Low-Abundant Cerebrospinal Fluid Proteome Alterations in Dementia with Lewy Bodies. Journal of Alzheimer’s Disease **34(2)**, 387–397.
55. **Enache D, Pereira JB, Jelic V, Winblad B, Nilsson P, Aarsland D and Bereczki E** (2020). Increased Cerebrospinal Fluid Concentration of ZnT3 Is Associated with Cognitive Impairment in Alzheimer’s Disease. Journal of Alzheimer’s Disease, **77(3)**, 1143–1155.
56. **Engelborghs S, Maertens K, Vloeberghs E, Aerts T, Somers N, Mariën P and De Deyn PP** (2006). Neuropsychological and behavioural correlates of CSF biomarkers in dementia. Neurochemistry International **48(4)**, 286–295.
57. **Ernst A, Morgenthaler N, Buerger K, Dodel R, Noelker C, Sommer N, Schwarz M, Koehrle J, Bergmann A and Hampel H** (2007). Procalcitonin is elevated in the cerebrospinal fluid of patients with dementia and acute neuroinflammation. Journal of Neuroimmunology **189(1–2)**, 169–174.
58. **Erskine D, Taylor JP, Firbank MJ, Patterson L, Onofrj M, O’Brien JT, McKeith IG, Attems J, Thomas AJ, Morris CM and Khundakar AA** (2016). Changes to the lateral geniculate nucleus in Alzheimer’s disease but not dementia with Lewy bodies. Neuropathology and Applied Neurobiology **42(4)**, 366–376.
59. **Ewers M, Mattsson N, Minthon L, Molinuevo JL, Antonell A, Popp J, Jessen F, Herukka S, Soininen H, Maetzler W, Leyhe T, Bürger K, Taniguchi M, Urakami K, Lista S, Dubois B, Blennow K and Hampel H** (2015). CSF biomarkers for the differential diagnosis of Alzheimer’s disease: A large‐scale international multicenter study. Alzheimer’s and Dementia **11(11)**, 1306–1315.
60. **Førland MG, Tysnes OB, Aarsland D, Maple‐Grødem J, Pedersen KF, Alves G and Lange J** (2020). The value of cerebrospinal fluid α‐synuclein and the tau/α‐synuclein ratio for diagnosis of neurodegenerative disorders with Lewy pathology. European Journal of Neurology **27(1)**, 43–50.
61. **Foulds PG, Yokota O, Thurston A, Davidson Y, Ahmed Z, Holton J, Thompson JC, Akiyama H, Arai T, Hasegawa M, Gerhard A, Allsop D and Mann DMA** (2012). Post mortem cerebrospinal fluid α-synuclein levels are raised in multiple system atrophy and distinguish this from the other α-synucleinopathies, Parkinson’s disease and Dementia with Lewy bodies. Neurobiology of Disease **45(1)**, 188–195.
62. **Gjerum L, Frederiksen KS, Henriksen OM, Law I, Bruun M, Simonsen AH, Mecocci P, Baroni M, Dottorini ME, Koikkalainen J, Lötjönen J and Hasselbalch SG** (2020). Evaluating 2-[^18^F]FDG-PET in differential diagnosis of dementia using a data-driven decision model. NeuroImage. Clinical **27**, 102267.
63. **Gmitterová K, Gawinecka J, Llorens F, Varges D, Valkovič P and Zerr I** (2020). Cerebrospinal fluid markers analysis in the differential diagnosis of dementia with Lewy bodies and Parkinson’s disease dementia. European Archives of Psychiatry and Clinical Neuroscience **270(4)**, 461–470.
64. **Gmitterova K, Varges D, Schmitz M, Zafar S, Maass F, Lingor P and Zerr I** (2020). Chromogranin A Analysis in the Differential Diagnosis Across Lewy Body Disorders. Journal of Alzheimer’s Disease **73(4)**, 1355–1361.
65. **Gómez-Tortosa E, Gonzalo I, Fanjul S, Sainz MJ, Cantarero S, Cemillán C, Yébenes JG and Del Ser T** (2003). Cerebrospinal Fluid Markers in Dementia With Lewy Bodies Compared With Alzheimer Disease. Archives of Neurology **60(9)**, 1218-1222.
66. **Gómez-Tortosa E, Sanders JL, Newell K and Hyman BT** (2001). Cortical neurons expressing calcium binding proteins are spared in dementia with Lewy bodies. Acta Neuropathologica **101(1)**, 36–42.
67. **Gonzalez MC, Ashton NJ, Gomes BF, Tovar-Rios DA, Blanc F, Karikari TK, Mollenhauer B, Pilotto A, Lemstra A, Paquet C, Abdelnour C, Kramberger MG, Bonanni L, Vandenberghe R, Hye A, Blennow K, Zetterberg H, Aarsland D and European–Dementia With Lewy Bodies (E-DLB) Consortium** (2022). Association of Plasma p-tau181 and p-tau231 Concentrations With Cognitive Decline in Patients With Probable Dementia With Lewy Bodies. JAMA Neurology **79(1)**, 32-37.
68. **Goossens J, Bjerke M, Struyfs H, Niemantsverdriet E, Somers C, Van Den Bossche T, Van Mossevelde S, De Vil B, Sieben A, Martin JJ, Cras P, Goeman J, De Deyn PP, Van Broeckhoven C, Van Der Zee J and Engelborghs S** (2017). No added diagnostic value of non-phosphorylated tau fraction (p-tau_rel_) in CSF as a biomarker for differential dementia diagnosis. Alzheimer’s Research and Therapy **9(1)**, 49.
69. **Hall S, Öhrfelt A, Constantinescu R, Andreasson U, Surova Y, Bostrom F, Nilsson C, Widner H, Decraemer H, Nägga K, Minthon L, Londos E, Vanmechelen E, Holmberg B, Zetterberg H, Blennow K and Hansson O** (2012). Accuracy of a Panel of 5 Cerebrospinal Fluid Biomarkers in the Differential Diagnosis of Patients With Dementia and/or Parkinsonian Disorders. Archives of Neurology **69(11)**, 1445-1452.
70. **Hampel H, Buerger K, Zinkowski R, Teipel SJ, Goernitz A andreasen N, Sjoegren M, DeBernardis J, Kerkman D, Ishiguro K, Ohno H, Vanmechelen E, Vanderstichele H, McCulloch C, Moller HJ, Davies P and Blennow K** (2004). Measurement of phosphorylated tau epitopes in the differential diagnosis of Alzheimer disease: A comparative cerebrospinal fluid study. Archives of General Psychiatry **61(1)**, 95–102.
71. **Hansen LA, Daniel SE, Wilcock GK and Love S** (1998). Frontal cortical synaptophysin in Lewy body diseases: Relation to Alzheimer’s disease and dementia. Journal of Neurology, Neurosurgery and Psychiatry **64(5)**, 653–656.
72. **Hansson O, Hall S, Öhrfelt A, Zetterberg H, Blennow K, Minthon L, Nägga K, Londos E, Varghese S, Majbour NK, Al-Hayani A and El-Agnaf OM** (2014). Levels of cerebrospinal fluid α-synuclein oligomers are increased in Parkinson’s disease with dementia and dementia with Lewy bodies compared to Alzheimer’s disease. Alzheimer’s Research and Therapy **6(3)**, 25.
73. **Hansson O, Santillo AF, Meeter LH, Nilsson K, Landqvist Waldö M, Nilsson C, Blennow K, Swieten JC and Janelidze S** (2019). CSF placental growth factor – a novel candidate biomarker of frontotemporal dementia. Annals of Clinical and Translational Neurology **6(5)**, 863–872.
74. **Harrington CR, Perry RH, Perry EK, Hurt J, McKeith IG, Roth M and Wischik CM** (1994). Senile dementia of Lewy body type and Alzheimer type are biochemically distinct in terms of paired helical filaments and hyperphosphorylated tau protein. Dementia **5(5)**, 215–228.
75. **Herbert MK, Aerts MB, Kuiperij HB, Claassen JAHR, Spies PE, Esselink RAJ, Bloem BR and Verbeek MM** (2014). Addition of MHPG to Alzheimer’s disease biomarkers improves differentiation of dementia with Lewy bodies from Alzheimer’s disease but not other dementias. Alzheimer’s and Dementia **10(4)**, 448-455.
76. **Heywood WE, Galimberti D, Bliss E, Sirka E, Paterson RW, Magdalinou NK, Carecchio M, Reid E, Heslegrave A, Fenoglio C, Scarpini E, Schott JM, Fox NC, Hardy J, Bahtia K, Heales S, Sebire NJ, Zetterburg H and Mills K** (2015). Identification of novel CSF biomarkers for neurodegeneration and their validation by a high-throughput multiplexed targeted proteomic assay. Molecular Neurodegeneration **10(1)**, 64.
77. **Howlett DR, Whitfield D, Johnson M, Attems J, O’Brien JT, Aarsland D, Lai MKP, Lee JH, Chen C, Ballard C, Hortobágyi T and Francis PT** (2015). Regional Multiple Pathology Scores Are Associated with Cognitive Decline in Lewy Body Dementias: Cognitive Decline in Lewy Body Dementias. Brain Pathology **25(4)**, 401–408.
78. **Ishiki A, Kamada M, Kawamura Y, Terao C, Shimoda F, Tomita N, Arai H and Furukawa, K** (2016). Glial fibrillar acidic protein in the cerebrospinal fluid of Alzheimer’s disease, dementia with Lewy bodies and frontotemporal lobar degeneration. Journal of Neurochemistry **136(2)**, 258–261.
79. **Jack CR, Wiste HJ, Botha H, Weigand SD, Therneau TM, Knopman DS, Graff-Radford J, Jones DT, Ferman TJ, Boeve BF, Kantarci K, Lowe VJ, Vemuri P, Mielke MM, Fields JA, Machulda MM, Schwarz CG, Senjem ML, Gunter JL and Petersen RC** (2019). The bivariate distribution of amyloid-β and tau: relationship with established neurocognitive clinical syndromes. Brain **142(10)**, 3230-3242.
80. **Janelidze S, Hertze J, Zetterberg H, Landqvist Waldö M, Santillo A, Blennow K and Hansson O** (2016). Cerebrospinal fluid neurogranin and YKL ‐40 as biomarkers of Alzheimer’s disease. Annals of Clinical and Translational Neurology **3(1)**, 12–20.
81. **Janelidze S, Lindqvist D, Francardo V, Hall S, Zetterberg H, Blennow K, Adler CH, Beach TG, Serrano GE, Van Westen D, Londos E, Cenci MA and Hansson O** (2015). Increased CSF biomarkers of angiogenesis in Parkinson disease. Neurology **85(21)**, 1834–1842.
82. **Johnson M, Ekonomou A, Hobbs C, Ballard CG, Perry RH and Perry EK** (2011). Neurogenic marker abnormalities in the hippocampus in dementia with Lewy bodies. Hippocampus **21(10)**, 1126–1136.
83. **Kaerst L, Kuhlmann A, Wedekind D, Stoeck K, Lange P and Zerr I** (2013). Using Cerebrospinal Fluid Marker Profiles in Clinical Diagnosis of Dementia with Lewy Bodies, Parkinson’s Disease and Alzheimer’s Disease. Journal of Alzheimer’s Disease **38(1)**, 63–73.
84. **Kapaki E, Paraskevas GP, Emmanouilidou E and Vekrellis K** (2013). The Diagnostic Value of CSF α-Synuclein in the Differential Diagnosis of Dementia with Lewy Bodies vsNormal Subjects and Patients with Alzheimer’s Disease. PLoS ONE **8(11)**, e81654.
85. **Kasuga K, Tokutake T, Ishikawa A, Uchiyama T, Tokuda T, Onodera O, Nishizawa M and Ikeuchi T** (2010). Differential levels of alpha-synuclein, beta-amyloid42 and tau in CSF between patients with dementia with Lewy bodies and Alzheimer's disease. Journal of Neurology, Neurosurgery and Psychiatry **81(6)**, 608–610.
86. **King E, O’Brien JT, Donaghy P, Morris C, Barnett N, Olsen K, Martin-Ruiz C, Taylor JP and Thomas AJ** (2018). Peripheral inflammation in prodromal Alzheimer’s and Lewy body dementias. Journal of Neurology, Neurosurgery and Psychiatry **89(4)**, 339–345.
87. **Klucken J, Ingelsson M, Shin Y, Irizarry MC, Hedley-Whyte ET, Frosch MP, Growdon JH, McLean PJ and Hyman BT** (2006). Clinical and biochemical correlates of insoluble α-synuclein in dementia with Lewy bodies. Acta Neuropathologica **111(2)**, 101–108.
88. **Koyama A, Hashimoto M, Tanaka H, Fujise N, Matsushita M, Miyagawa Y, Hatada Y, Fukuhara R, Hasegawa N, Todani S, Matsukuma K, Kawano M and Ikeda M** (2016). Malnutrition in Alzheimer’s Disease, Dementia with Lewy Bodies and Frontotemporal Lobar Degeneration: Comparison Using Serum Albumin, Total Protein and Hemoglobin Level. PLoS One **11(6)**, e0157053.
89. **Kronimus Y, Albus A, Balzer-Geldsetzer M, Straub S, Semler E, Otto M, Klotsche J, Dodel R, LANDSCAPE Consortium and Mengel D** (2016). Naturally Occurring Autoantibodies against Tau Protein Are Reduced in Parkinson’s Disease Dementia. PLoS One **11(11)**, e0164953.
90. **Laske C, Fallgatter AJ, Stransky E, Hagen K, Berg D and Maetzler W** (2011). Decreased α-Synuclein Serum Levels in Patients with Lewy Body Dementia Compared to Alzheimer’s Disease Patients and Control Subjects. Dementia and Geriatric Cognitive Disorders **31(6)**, 413–416.
91. **Lehnert S, Jesse S, Rist W, Steinacker P, Soininen H, Herukka SK, Tumani H, Lenter M, Oeckl P, Ferger B, Hengerer B and Otto M** (2012). ITRAQ and multiple reaction monitoring as proteomic tools for biomarker search in cerebrospinal fluid of patients with Parkinson’s disease dementia. Experimental Neurology **234(2)**, 499–505.
92. **Lin YS, Lee WJ, Wang SJ and Fuh JL** (2018). Levels of plasma neurofilament light chain and cognitive function in patients with Alzheimer or Parkinson disease. Scientific Reports **8(1)**, 17368.
93. **Lindqvist D, Hall S, Surova Y, Nielsen HM, Janelidze S, Brundin L and Hansson O** (2013). Cerebrospinal fluid inflammatory markers in Parkinson’s disease – Associations with depression, fatigue and cognitive impairment*.* Brain, Behavior and Immunity **33**, 183–189.
94. **Lippa CF, Smith TW and Perry E** (1999). Dementia with Lewy bodies: Choline acetyltransferase parallels nucleus basalis pathology. Journal of Neural Transmission **106(5–6)**, 525–535.
95. **Llorens F, Schmitz M, Gloeckner SF, Kaerst L, Hermann P, Schmidt C, Varges D and Zerr I** (2015). Increased albumin CSF/serum ratio in dementia with Lewy bodies. Journal of the Neurological Sciences **358(1–2)**, 398–403.
96. **Llorens F, Villar‐Piqué A, Schmitz M, Diaz‐Lucena D, Wohlhage M, Hermann P, Goebel S, Schmidt I, Glatzel M, Hauw JJ, Sikorska B, Liberski PP, Riggert J, Ferrer I and Zerr I** (2020). Plasma total prion protein as a potential biomarker for neurodegenerative dementia: Diagnostic accuracy in the spectrum of prion diseases. Neuropathology and Applied Neurobiology **46(3)**, 240–254.
97. **Longobardi A, Nicsanu R, Bellini S, Squitti R, Catania M, Tiraboschi P, Saraceno C, Ferrari C, Zanardini R, Binetti G, Di Fede G, Benussi L and Ghidoni R** (2022). Cerebrospinal Fluid EV Concentration and Size Are Altered in Alzheimer’s Disease and Dementia with Lewy Bodies. Cells **11(3)**, 462.
98. **Love S** (2001). Damage to nuclear DNA in Lewy body disease: Neuroreport **12(12)**, 2725–2729.
99. **Lue LF, Schmitz CT, Snyder NL, Chen K, Walker, DG, Davis KJ, Belden C, Caviness JN, Driver-Dunckley E, Adler CH, Sabbagh MN and Shill HA** (2016). Converging mediators from immune and trophic pathways to identify Parkinson disease dementia. Neurology Neuroimmunology & Neuroinflammation **3(1)**, e193.
100. **Luo X, Hou L, Shi H, Zhong X, Zhang Y, Zheng D, Tan Y, Hu G, Mu N, Chan J, Chen X, Fang Y, Wu F, He H and Ning Y** (2013). CSF levels of the neuronal injury biomarker visinin-like protein-1 in Alzheimer’s disease and dementia with Lewy bodies. Journal of Neurochemistry **127(5)**, 681–690.
101. **Maetzler W, Berg D, Synofzik M, Brockmann K, Godau J, Melms A, Gasser T, Hörnig S and Langkamp M** (2011). Autoantibodies Against Amyloid and Glial-Derived Antigens are Increased in Serum and Cerebrospinal Fluid of Lewy Body-Associated Dementias. Journal of Alzheimer’s Disease **26(1)**, 171–179.
102. **Maetzler W, Deleersnijder W, Hanssens V, Bernard A, Brockmann K, Marquetand J, Wurster I, Rattay TW, Roncoroni L, Schaeffer E, Lerche S, Apel A, Deuschle C and Berg D** (2016). GDF15/MIC1 and MMP9 Cerebrospinal Fluid Levels in Parkinson’s Disease and Lewy Body Dementia. PLoS One **11(3)**, e0149349.
103. **Maetzler W, Schmid B, Synofzik M, Schulte C, Riester K, Huber H, Brockmann K, Gasser T, Berg D and Melms A** (2010). The CST3 BB Genotype and Low Cystatin C Cerebrospinal Fluid Levels are Associated with Dementia in Lewy Body Disease. Journal of Alzheimer’s Disease **19(3)**, 937–942.
104. **Maetzler W, Schmid SP, Wurster I, Liepelt I, Gaenslen A, Gasser T and Berg D** (2011). Reduced but not oxidized cerebrospinal fluid glutathione levels are lowered in Lewy body diseases. Movement Disorders **26(1)**, 176–181.
105. **Maetzler W, Stapf AK, Schulte C, Hauser AK, Lerche S, Wurster I, Schleicher E, Melms A and Berg D** (2011). Serum and Cerebrospinal Fluid Uric Acid Levels in Lewy Body Disorders: Associations with Disease Occurrence and Amyloid-β Pathway. Journal of Alzheimer’s Disease **27(1)**, 119–126.
106. **Maetzler W, Stoycheva V, Schmid B, Schulte C, Hauser AK, Brockmann K, Melms A, Gasser T and Berg D** (2010). Neprilysin Activity in Cerebrospinal Fluid is Associated with Dementia and Amyloid-β42 Levels in Lewy Body Disease. Journal of Alzheimer’s Disease **22(3)**, 933–938.
107. **Maetzler W, Tian Y, Baur SM, Gauger T, Odoj B, Schmid B, Schulte C, Deuschle C, Heck S, Apel A, Melms A, Gasser T and Berg D** (2012). Serum and Cerebrospinal Fluid Levels of Transthyretin in Lewy Body Disorders with and without Dementia. PLoS One **7(10)**, e48042.
108. **Mantle D, Falkous G, Ishiura S, Perry RH and Perry EK** (1995). Comparison of cathepsin protease activities in brain tissue from normal cases and cases with Alzheimer’s disease, Lewy body dementia, Parkinson’s disease and Huntington’s disease. Journal of the Neurological Sciences **131(1)**, 65–70.
109. **Martin-Ruiz C, Lawrence S, Piggott M, Kuryatov A, Lindstrom J, Gotti C, Cookson MR, Perry RH, Jaros E, Perry EK and Court JA** (2002). Nicotinic receptors in the putamen of patients with dementia with Lewy bodies and Parkinson's disease: relation to changes in alpha-synuclein expression. Neuroscience Letters **335(2)**, 134-138.
110. **Meyne F, Gloeckner SF, Ciesielczyk B, Heinemann U, Krasnianski A, Meissner B and Zerr I** (2009). Total Prion Protein Levels in the Cerebrospinal Fluid are Reduced in Patients with Various Neurological Disorders. Journal of Alzheimer’s Disease **17(4)**, 863–873.
111. **Miller RL, Dhavale DD, O’Shea JY, Andruska KM, Liu J, Franklin EE, Buddhala C, Loftin SK, Cirrito JR, Perrin RJ, Cairns NJ, Campbell MC, Perlmutter JS and Kotzbauer PT** (2022). Quantifying regional α ‐synuclein, amyloid β and tau accumulation in lewy body dementia. Annals of Clinical and Translational Neurology **9(2)**, 106–121.
112. **Mollenhauer B, Bibl M, Esselmann H, Steinacker P, Trenkwalder C, Wiltfang J and Otto M** (2007). Tauopathies and synucleinopathies: Do cerebrospinal fluid β-amyloid peptides reflect disease-specific pathogenesis? Journal of Neural Transmission **114(7)**, 919–927.
113. **Mollenhauer B, Bibl M, Trenkwalder C, Stiens G, Cepek L, Steinacker P, Ciesielczyk B, Neubert K, Wiltfang J, Kretzschmar HA, Poser S and Otto M** (2005). Follow-up investigations in cerebrospinal fluid of patients with dementia with Lewy bodies and Alzheimer’s disease. Journal of Neural Transmission **112(7)**, 933–948.
114. **Mollenhauer B, Bibl M, Wiltfang J, Steinacker P, Ciesielczyk B, Neubert K, Trenkwalder C and Otto M** (2006). Total tau protein, phosphorylated tau (181p) protein, β-amyloid1–42 and β-amyloid1–40 in cerebrospinal fluid of patients with dementia with Lewy bodies. Clinical Chemistry and Laboratory Medicine **44(2)**, 192-195.
115. **Mollenhauer B, Cepek L, Bibl M, Wiltfang J, Schulz-Schaeffer WJ, Ciesielczyk B, Neumann M, Steinacker P, Kretzschmar HA, Poser S, Trenkwalder C and Otto M** (2005). Tau Protein, Aβ42 and S-100B Protein in Cerebrospinal Fluid of Patients with Dementia with Lewy Bodies. Dementia and Geriatric Cognitive Disorders **19(2–3)**, 164–170.
116. **Mollenhauer B, Locascio JJ, Schulz-Schaeffer W, Sixel-Döring F, Trenkwalder C and Schlossmacher MG** (2011). α-Synuclein and tau concentrations in cerebrospinal fluid of patients presenting with parkinsonism: A cohort study. The Lancet Neurology **10(3)**, 230–240.
117. **Mollenhauer B, Steinacker P, Bahn E, Bibl M, Brechlin P, Schlossmacher MG, Locascio JJ, Wiltfang J, Kretzschmar HA, Poser S, Trenkwalder C and Otto M** (2007). Serum Heart-Type Fatty Acid-Binding Protein and Cerebrospinal Fluid Tau: Marker Candidates for Dementia with Lewy Bodies. Neuro-Degenerative Diseases **4(5)**, 366-375.
118. **Mollenhauer B, Trenkwalder C, Von Ahsen N, Bibl M, Steinacker P, Brechlin P, Schindehuette J, Poser S, Wiltfang J and Otto M** (2006). Beta-Amlyoid 1–42 and Tau-Protein in Cerebrospinal Fluid of Patients with Parkinson’s Disease Dementia. Dementia and Geriatric Cognitive Disorders **22(3)**, 200–208.
119. **Montine TJ, Shi M, Quinn JF, Peskind ER, Craft S, Ginghina C, Chung KA, Kim H, Galasko DR, Jankovic J, Zabetian CP, Leverenz JB and Zhang J** (2010). CSF Aβ42 and tau in Parkinson’s disease with cognitive impairment. Movement Disorders **25(15)**, 2682–2685.
120. **Morenas-Rodríguez E, Alcolea D, Suárez-Calvet M, Muñoz-Llahuna L, Vilaplana E, Sala I, Subirana A, Querol-Vilaseca M, Carmona-Iragui M, Illán-Gala I, Ribosa-Nogué R, Blesa R, Haass C, Fortea J and Lleó A** (2019). Different pattern of CSF glial markers between dementia with Lewy bodies and Alzheimer’s disease. Scientific Reports **9(1)**, 7803.
121. **Mukaetova-Ladinska EB, Andras A, Milne J, Abdel-All Z, Borr I, Jaros E, Perry RH, Honer WG, Cleghorn A, Doherty J, McIntosh G, Perry EK, Kalaria RN and McKeith IG** (2013). Synaptic Proteins and Choline Acetyltransferase Loss in Visual Cortex in Dementia With Lewy Bodies. Journal of Neuropathology and Experimental Neurology **72(1)**, 53–60.
122. **Mukaetova-Ladinska EB, Milne J, Andras A, Abdel-All Z, Cerejeira J, Greally E, Robson J, Jaros E, Perry R, McKeith IG, Brayne C, Xuereb J, Cleghorn A, Doherty J, McIntosh G and Milton I** (2008). Alpha- and Gamma-Synuclein Proteins Are Present in Cerebrospinal Fluid and Are Increased in Aged Subjects with Neurodegenerative and Vascular Changes. Dementia and Geriatric Cognitive Disorders **26(1)**, 32–42.
123. **Müller M, Claassen JA, Kuiperij HB and Verbeek MM** (2015). Cerebrospinal Fluid NrCAM is not a Suitable Biomarker to Discriminate between Dementia Disorders – A Pilot Study. Journal of Alzheimer’s Disease **46(3)**, 605–609.
124. **Mulugeta E, Londos E, Ballard C, Alves G, Zetterberg H, Blennow K, Skogseth R, Minthon L and Aarsland D** (2011). CSF amyloid 38 as a novel diagnostic marker for dementia with Lewy bodies. Journal of Neurology, Neurosurgery and Psychiatry **82(2)**, 160–164.
125. **Mulugeta E, Londos E, Hansson O, Ballard C, Skogseth R, Minthon L, Blennow K, Zetterberg H and Aarsland D** (2011). Cerebrospinal Fluid Levels of sAPPα and sAPPβ in Lewy Body and Alzheimer's Disease: Clinical and Neurochemical Correlates. International Journal of Alzheimer’s Disease **2011**, 495025.
126. **Musaeus CS, Gleerup HS, Høgh P, Waldemar G, Hasselbalch SG and Simonsen AH** (2020). Cerebrospinal Fluid/Plasma Albumin Ratio as a Biomarker for Blood-Brain Barrier Impairment Across Neurodegenerative Dementias. Journal of Alzheimer’s Disease **75(2)**, 429–436.
127. **Nielsen HM, Hall S, Surova Y, Nägga K, Nilsson C, Londos E, Minthon L, Hansson O and Wennström M** (2014). Low Levels of Soluble NG2 in Cerebrospinal Fluid from Patients with Dementia with Lewy Bodies. Journal of Alzheimer’s Disease **40(2)**, 343–350.
128. **Nielsen HM, Londos E, Minthon L and Janciauskiene SM** (2007). Soluble adhesion molecules and angiotensin-converting enzyme in dementia. Neurobiology of Disease **26(1)**, 27–35.
129. **Nielsen HM, Minthon L, Londos E, Blennow K, Miranda E, Perez J, Crowther DC, Lomas DA and Janciauskiene SM** (2007). Plasma and CSF serpins in Alzheimer disease and dementia with Lewy bodies. Neurology **69(16)**, 1569–1579.
130. **Noguchi-Shinohara M, Tokuda T, Yoshita M, Kasai T, Ono K, Nakagawa M, El-Agnaf OMA and Yamada M** (2009). CSF α-synuclein levels in dementia with Lewy bodies and Alzheimer’s disease. Brain Research **1251**, 1–6.
131. **Oeckl P, Halbgebauer S, Anderl-Straub S, Von Arnim CAF, Diehl-Schmid J, Froelich L, Grimmer T, Hausner L, Denk J, Jahn H, Steinacker P, Weishaupt JH, Ludolph AC and Otto M** (2020). Targeted Mass Spectrometry Suggests Beta-Synuclein as Synaptic Blood Marker in Alzheimer’s Disease. Journal of Proteome Research **19(3)**, 1310–1318.
132. **Öhrfelt A, Grognet P, Andreasen N, Wallin A, Vanmechelen E, Blennow K and Zetterberg H** (2009). Cerebrospinal fluid α-synuclein in neurodegenerative disorders—A marker of synapse loss? Neuroscience Letters **450(3)**, 332–335.
133. **Olsson B, Portelius E, Cullen NC, Sandelius Å, Zetterberg H, Andreasson U, Höglund K, Irwin DJ, Grossman M, Weintraub D, Chen-Plotkin A, Wolk D, McCluskey L, Elman L, Shaw LM, Toledo JB, McBride J, Hernandez-Con P, Lee VM, Trojanowski JQ and Blennow K** (2019). Association of Cerebrospinal Fluid Neurofilament Light Protein Levels With Cognition in Patients With Dementia, Motor Neuron Disease and Movement Disorders. JAMA Neurology **76(3)**, 318-325.
134. **Palumbo B, Siepi D, Sabalich I, Tranfaglia C and Parnetti L** (2008). Cerebrospinal fluid neuron-specific enolase: A further marker of Alzheimer’s disease? Functional Neurology **23(2)**, 93–96.
135. **Park H, Lee K, Park ES, Oh S, Yan R, Zhang J, Beach TG, Adler CH, Voronkov M, Braithwaite SP, Stock, JB and Mouradian MM** (2016). Dysregulation of protein phosphatase 2A in parkinson disease and dementia with lewy bodies. Annals of Clinical and Translational Neurology **3(10)**, 769–780.
136. **Parnetti L, Tiraboschi P, Lanari A, Peducci M, Padiglioni C, D’Amore C, Pierguidi L, Tambasco N, Rossi A and Calabresi P** (2008). Cerebrospinal Fluid Biomarkers in Parkinson’s Disease with Dementia and Dementia with Lewy Bodies. Biological Psychiatry **64(10)**, 850–855.
137. **Paterson RW, Slattery CF, Poole T, Nicholas JM, Magdalinou NK, Toombs J, Chapman, MD, Lunn MP, Heslegrave AJ, Foiani, MS, Weston PSJ, Keshavan A, Rohrer JD, Rossor MN, Warren JD, Mummery CJ, Blennow K, Fox NC, Zetterberg H and Schott JM** (2018). Cerebrospinal fluid in the differential diagnosis of Alzheimer’s disease: Clinical utility of an extended panel of biomarkers in a specialist cognitive clinic. Alzheimer’s Research and Therapy **10(1)**, 32.
138. **Přikrylová Vranová H, Hényková E, Mareš J, Kaiserová M, Menšíková K, Vaštík M, Hluštík P, Zapletalová J, Strnad M, Stejskal D and Kaňovský P** (2016). Clusterin CSF levels in differential diagnosis of neurodegenerative disorders. Journal of the Neurological Sciences **361**, 117–121.
139. **Quadalti C, Calandra-Buonaura G, Baiardi S, Mastrangelo A, Rossi M, Zenesini C, Giannini G, Candelise N, Sambati L, Polischi B, Plazzi G, Capellari S, Cortelli P and Parchi P** (2021). Neurofilament light chain and α-synuclein RT-QuIC as differential diagnostic biomarkers in parkinsonisms and related syndromes. NPJ Parkinson's Disease **7(1)**, 93.
140. **Sahin HA, Emre M, Ziabreva I, Perry E, Celasun B and Perry** R (2006). The distribution pattern of pathology and cholinergic deficits in amygdaloid complex in Alzheimer’s disease and dementia with Lewy bodies. Acta Neuropathologica **111(2)**, 115–125.
141. **Samuel W, Alford M, Hofstetter CR and Hansen L** (1997). Dementia with Lewy bodies versus pure Alzheimer disease: Differences in cognition, neuropathology, cholinergic dysfunction and synapse density. Journal of Neuropathology and Experimental Neurology **56(5)**, 499–508.
142. **Schoonenboom NSM, Reesink FE, Verwey NA, Kester MI, Teunissen CE, Van De Ven PM, Pijnenburg YAL, Blankenstein MA, Rozemuller AJ, Scheltens P and Van Der Flier WM** (2012). Cerebrospinal fluid markers for differential dementia diagnosis in a large memory clinic cohort. Neurology **78(1)**, 47–54.
143. **Schultz K, Nilsson K, Nielsen JE, Lindquis SG, Hjermind LE, Andersen BB, Wallin A, Nilsson C and Petersén Å** (2010). Transthyretin as a potential CSF biomarker for Alzheimer’s disease and dementia with Lewy bodies: Effects of treatment with cholinesterase inhibitors: CSF transthyretin as a biomarker. European Journal of Neurology **17(3)**, 456–460.
144. **Schulz I, Kruse N, Gera RG, Kremer T, Cedarbaum J, Barbour R, Zago W, Schade S, Otte B, Bartl M, Hutten SJ, Trenkwalder C and Mollenhauer B** (2021). Systematic Assessment of 10 Biomarker Candidates Focusing on α‐Synuclein‐Related Disorders. Movement Disorders **36(12)**, 2874–2887.
145. **Sinclair LI, Kumar A, Darreh-Shori T and Love S** (2019). Visual hallucinations in Alzheimer’s disease do not seem to be associated with chronic hypoperfusion of to visual processing areas V2 and V3 but may be associated with reduced cholinergic input to these areas. Alzheimer’s Research and Therapy **11(1)**, 80.
146. **Singh P, Hanson PS and Morris CM** (2017). SIRT1 ameliorates oxidative stress induced neural cell death and is down-regulated in Parkinson’s disease. BMC Neuroscience **18(1)**, 46.
147. **Skillbäck T, Farahmand BY, Rosén C, Mattsson N, Nägga K, Kilander L, Religa D, Wimo A, Winblad B, Schott JM, Blennow K, Eriksdotter M and Zetterberg H** (2015). Cerebrospinal fluid tau and amyloid-β 1-42 in patients with dementia. Brain **138(9)**, 2716–2731.
148. **Slaets S, Le Bastard N, Theuns J, Sleegers K, Verstraeten A, De Leenheir E, Luyckx J, Martin JJ, Van Broeckhoven C and Engelborghs S** (2013). Amyloid Pathology Influences Aβ1-42 Cerebrospinal Fluid Levels in Dementia with Lewy Bodies. Journal of Alzheimer’s Disease **35(1)**, 137–146.
149. **Slaets S, Vanmechelen E, Le Bastard N, Decraemer H, Vandijck M, Martin J, De Deyn PP and Engelborghs** S (2014). Increased CSF α‐synuclein levels in Alzheimer’s disease: Correlation with tau levels. Alzheimer’s and Dementia **10(5 Suppl)**, S290-298.
150. **Smith BR, Nelson KM, Kemper LJ, Leinonen-Wright K, Petersen A, Keene CD and Ashe KH** (2019). A soluble tau fragment generated by caspase-2 is associated with dementia in Lewy body disease. Acta Neuropathologica Communications **7(1)**, 124.
151. **Sohma H, Imai S, Takei N, Honda H, Matsumoto K, Utsumi K, Matsuki K, Hashimoto E, Saito T and Kokai Y** (2013). Evaluation of annexin A5 as a biomarker for Alzheimer’s disease and dementia with lewy bodies. Frontiers in Aging Neuroscience **5**, 15.
152. **Spies PE, Melis RJF, Sjögren MJC, Olde Rikkert MGM and Verbeek MM** (2009). Cerebrospinal Fluid α-Synuclein Does Not Discriminate Between Dementia Disorders. Journal of Alzheimer’s Disease **16(2)**, 363–369.
153. **Spies PE, Slats D, Sjögren JMC, Kremer BPH, Verhey FRJ, Rikkert MGMO and Verbeek MM** (2010). The cerebrospinal fluid amyloid beta42/40 ratio in the differentiation of Alzheimer’s disease from non-Alzheimer’s dementia. Current Alzheimer Research **7(5)**, 470–476.
154. **Steinacker P, Mollenhauer B, Bibl M, Cepek L, Esselmann H, Brechlin P, Lewczuk P, Poser S, Kretzschmar HA, Wiltfang J, Trenkwalder C and Otto M** (2004). Heart fatty acid binding protein as a potential diagnostic marker for neurodegenerative diseases. Neuroscience Letters **370(1)**, 36–39.
155. **Stuendl A, Kraus T, Chatterjee M, Zapke B, Sadowski B, Moebius W, Hobert MA, Deuschle C, Brockmann K, Maetzler W, Mollenhauer B and Schneider A** (2021). α-Synuclein in Plasma-Derived Extracellular Vesicles Is a Potential Biomarker of Parkinson's Disease. Movement Disorders **36(11)**, 2508–2518.
156. **Stuendl A, Kunadt M, Kruse N, Bartels C, Moebius W, Danzer KM, Mollenhauer B and Schneider A** (2016). Induction of α-synuclein aggregate formation by CSF exosomes from patients with Parkinson’s disease and dementia with Lewy bodies. Brain **139(2)**, 481–494.
157. **Surendranathan A, Su L, Mak E, Passamonti L, Hong YT, Arnold R, Vázquez Rodríguez P, Bevan-Jones WR, Brain SAE, Fryer TD, Aigbirhio FI, Rowe JB and O’Brien JT** (2018). Early microglial activation and peripheral inflammation in dementia with Lewy bodies. Brain **141(12)**, 3415–3427.
158. **Suzuki K, Suzuki S, Ishii Y, Fujita H, Matsubara T, Okamura M, Sakuramoto H and Hirata K** (2019). Serum insulin‐like growth factor‐1 levels in neurodegenerative diseases. Acta Neurologica Scandinavica **139(6)**, 563–567.
159. **Suzuki M, Desmond TJ, Albin RL and Frey KA** (2002). Striatal monoaminergic terminals in Lewy body and Alzheimer’s dementias. Annals of Neurology **51(6)**, 767–771.
160. **Tabet N, Walker Z, Mantle D, Costa D and Orrell M** (2003). In vivo dopamine pre-synaptic receptors and antioxidant activities in patients with Alzheimer's disease, dementia with Lewy bodies and in controls. A preliminary report**.** Dementia and Geriatric Cognitive Disorders **16(1)**, 46–51.
161. **Tateno F, Sakakibara R, Kawai T, Kishi M and Murano T** (2012). Alpha-synuclein in the Cerebrospinal Fluid Differentiates Synucleinopathies (Parkinson Disease, Dementia With Lewy Bodies, Multiple System Atrophy) From Alzheimer Disease. Alzheimer Disease and Associated Disorders **26(3)**, 213–216.
162. **Teichmann M, Epelbaum S, Samri D, Levy Nogueira M, Michon A, Hampel H, Lamari F and Dubois B** (2017). Free and Cued Selective Reminding Test – accuracy for the differential diagnosis of Alzheimer’s and neurodegenerative diseases: A large‐scale biomarker‐characterized monocenter cohort study (ClinAD). Alzheimer’s and Dementia **13(8)**, 913–923.
163. **Timmer NM, Herbert MK, Claassen JAHR, Kuiperij HB and Verbeek MM** (2015). Total glutamine synthetase levels in cerebrospinal fluid of Alzheimer’s disease patients are unchanged. Neurobiology of Aging **36(3)**, 1271–1273.
164. **Tong M, Dong M and De La Monte SM** (2009). Brain Insulin-Like Growth Factor and Neurotrophin Resistance in Parkinson’s Disease and Dementia with Lewy Bodies: Potential Role of Manganese Neurotoxicity. Journal of Alzheimer’s Disease **16(3)**, 585–599.
165. **Trotti LM, Bliwise DL, Keating GL, Rye DB and Hu WT** (2021). Cerebrospinal Fluid Hypocretin and Nightmares in Dementia Syndromes. Dementia and Geriatric Cognitive Disorders Extra **11(1)**, 19–25.
166. **Tu H, Zhang ZW, Qiu L, Lin Y, Jiang M, Chia SY, Wei Y, Ng ASL, Reynolds R, Tan EK and Zeng L** (2022). Increased expression of pathological markers in Parkinson’s disease dementia post-mortem brains compared to dementia with Lewy bodies. BMC Neuroscience **23(1)**, 3.
167. **Tuna G, Yener GG, Oktay G, İşlekel GH and Kİrkalİ FG** (2018). Evaluation of Matrix Metalloproteinase-2 (MMP-2) and -9 (MMP-9) and Their Tissue Inhibitors (TIMP-1 and TIMP-2) in Plasma from Patients with Neurodegenerative Dementia. Journal of Alzheimer’s Disease **66(3)**, 1265–1273.
168. **Usenko TS, Nikolaev MA, Miliukhina IV, Bezrukova AI, Senkevich KA, Gomzyakova NA, Beltceva YA, Zalutskaya NM, Gracheva EV, Timofeeva AA, Petrova OA, Semenov AV, Lubimova NE, Totolyan AA and Pchelina SN** (2020). Plasma cytokine profile in synucleinophaties with dementia. Journal of Clinical Neuroscience **78**, 323–326.
169. **Vallortigara J, Rangarajan S, Whitfield D, Alghamdi A, Howlett D, Hortobágyi T, Johnson M, Attems J, Ballard C, Thomas A, O’Brien J, Aarsland D and Francis P** (2014). Dynamin1 concentration in the prefrontal cortex is associated with cognitive impairment in Lewy body dementia. F1000 Research **3**, 108.
170. **Vallortigara J, Whitfield D, Quelch W, Alghamdi A, Howlett D, Hortobágyi T, Johnson M, Attems J, O’Brien JT, Thomas A, Ballard CG, Aarsland D and Francis PT** (2016). Decreased Levels of VAMP2 and Monomeric Alpha-Synuclein Correlate with Duration of Dementia. Journal of Alzheimer’s Disease, **50(1)**, 101–110.
171. **Van Den Berge SA, Kevenaar JT, Sluijs JA and Hol EM** (2012). Dementia in Parkinson’s Disease Correlates with α -Synuclein Pathology but Not with Cortical Astrogliosis. Parkinson’s Disease **2012**, 420957.
172. **Van Steenoven I, Koel-Simmelink MJA, Vergouw LJM, Tijms BM, Piersma SR, Pham TV, Bridel C, Ferri GL, Cocco C, Noli B, Worley PF, Xiao MF, Xu D, Oeckl P, Otto M, Van Der Flier WM, De Jong FJ, Jimenez CR, Lemstra AW and Teunissen CE** (2020). Identification of novel cerebrospinal fluid biomarker candidates for dementia with Lewy bodies: A proteomic approach. Molecular Neurodegeneration **15(1)**, 36.
173. **Van Steenoven I, Majbour NK, Vaikath NN, Berendse HW, Van Der Flier WM, Van De Berg WDJ, Teunissen CE, Lemstra AW and El-Agnaf OMA** (2018). α-Synuclein species as potential cerebrospinal fluid biomarkers for dementia with lewy bodies: CSF α-Synuclein species as biomarkers for DLB. Movement Disorders **33(11)**, 1724–1733.
174. **Van Steenoven I, Noli B, Cocco C, Ferri GL, Oeckl P, Otto M, Koel-Simmelink MJA, Bridel C, Van Der Flier WM, Lemstra AW and Teunissen CE** (2019). VGF Peptides in Cerebrospinal Fluid of Patients with Dementia with Lewy Bodies. International Journal of Molecular Sciences **20(19)**, 4674.
175. **Vanderstichele H, De Vreese K, Blennow K, Andreasen N, Sindic C, Ivanoiu A, Hampel H, Bürger K, Parnetti L, Lanari A, Padovani A, DiLuca M, Bläser M, Olsson AO, Pottel H, Hulstaert F and Vanmechelen E** (2006). Analytical performance and clinical utility of the INNOTEST PHOSPHO-TAU181P assay for discrimination between Alzheimer’s disease and dementia with Lewy bodies. Clinical Chemistry and Laboratory Medicine **44(12)**, 1472–1480.
176. **Ventriglia M, Zanardini R, Bonomini C, Zanetti O, Volpe D, Pasqualetti P, Gennarelli M and Bocchio-Chiavetto L** (2013). Serum Brain-Derived Neurotrophic Factor Levels in Different Neurological Diseases. BioMed Research International **2013**, 901082.
177. **Villar-Piqué A, Schmitz M, Hermann P, Goebel S, Bunck T, Varges D, Ferrer I, Riggert J, Llorens F and Zerr I** (2019). Plasma YKL-40 in the spectrum of neurodegenerative dementia. Journal of Neuroinflammation **16(1)**, 145.
178. **Vranová HP, Hényková E, Kaiserová M, Menšíková K, Vaštík M, Mareš J, Hluštík P, Zapletalová J, Strnad M, Stejskal D and Kaňovský P** (2014). Tau protein, beta-amyloid₁₋₄₂ and clusterin CSF levels in the differential diagnosis of Parkinsonian syndrome with dementia. Journal of the Neurological Sciences **343(1–2)**, 120–124.
179. **Wada-Isoe K, Imamura K, Kitamaya M, Kowa H and Nakashima K** (2008). Serum heart-fatty acid binding protein levels in patients with Lewy body disease. Journal of the Neurological Sciences **266(1–2)**, 20–24.
180. **Wada-Isoe K, Kitayama M, Nakaso K and Nakashima K** (2007). Diagnostic markers for diagnosing dementia with Lewy bodies: CSF and MIBG cardiac scintigraphy study. Journal of the Neurological Sciences **260(1–2)**, 33–37.
181. **Walker L, McAleese KE, Thomas AJ, Johnson M, Martin-Ruiz C, Parker C, Colloby SJ, Jellinger K and Attems J** (2015). Neuropathologically mixed Alzheimer’s and Lewy body disease: Burden of pathological protein aggregates differs between clinical phenotypes. Acta Neuropathologica **129(5)**, 729–748.
182. **Wang J, Zheng B, Yang S, Hu M and Wang JH** (2020). Differential Circulating Levels of Naturally Occurring Antibody to α-Synuclein in Parkinson’s Disease Dementia, Alzheimer’s Disease and Vascular Dementia. Frontiers in Aging Neuroscience **12**, 571437.
183. **Wang T, Yuan F, Chen Z, Zhu S, Chang Z, Yang W, Que R, Cao P, Chao Y, Chan L, Pan Y, Wang Y, Xu L, Lyu Q, Chan P, Yenari MA, Tan EK and Wang Q** (2020). Vascular, inflammatory and metabolic risk factors in relation to dementia in Parkinson’s disease patients with type 2 diabetes mellitus. Aging **12(15)**, 15682-15704.
184. **Wellington H, Paterson RW, Portelius E, Törnqvist U, Magdalinou N, Fox NC, Blennow K, Schott JM and Zetterberg H** (2016). Increased CSF neurogranin concentration is specific to Alzheimer disease. Neurology **86(9)**, 829–835.
185. **Wennström M, Hall S, Nägga K, Londos E, Minthon L and Hansson O** (2015). Cerebrospinal fluid levels of IL-6 are decreased and correlate with cognitive status in DLB patients. Alzheimer’s Research and Therapy **7(1)**, 63.
186. **Wennström M, Surova Y, Hall S, Nilsson C, Minthon L, Boström F, Hansson O and Nielsen HM** (2013). Low CSF Levels of Both α-Synuclein and the α-Synuclein Cleaving Enzyme Neurosin in Patients with Synucleinopathy. PLoS One **8(1)**, e53250.
187. **Wennström M, Surova Y, Hall S, Nilsson C, Minthon L, Hansson O and Nielsen HM** (2015). The Inflammatory Marker YKL-40 Is Elevated in Cerebrospinal Fluid from Patients with Alzheimer’s but Not Parkinson’s Disease or Dementia with Lewy Bodies. PLoS One **10(8)**, e0135458.
188. **Whitfield DR, Vallortigara J, Alghamdi A, Howlett D, Hortobágyi T, Johnson M, Attems J, Newhouse S, Ballard C, Thomas AJ, O’Brien JT, Aarsland D and Francis PT** (2014). Assessment of ZnT3 and PSD95 protein levels in Lewy body dementias and Alzheimer’s disease: Association with cognitive impairment. Neurobiology of Aging **35(12)**, 2836–2844.
189. **Willemse EAJ, De Vos A, Herries EM, Andreasson U, Engelborghs S, Van Der Flier WM, Scheltens P, Crimmins D, Ladenson JH, Vanmechelen E, Zetterberg H, Fagan AM, Blennow K, Bjerke M and Teunissen CE** (2018). Neurogranin as Cerebrospinal Fluid Biomarker for Alzheimer Disease: An Assay Comparison Study. Clinical Chemistry **64(6)**, 927–937.
190. **Wills J, Jones J, Haggerty T, Duka V, Joyce JN and Sidhu A** (2010). Elevated tauopathy and alpha-synuclein pathology in postmortem Parkinson’s disease brains with and without dementia. Experimental Neurology **225(1)**, 210–218.
191. **Yang W, Woltjer RL, Sokal I, Pan C, Wang Y, Brodey M, Peskind ER, Leverenz JB, Zhang J, Perl DP, Galasko DR and Montine TJ** (2007). Quantitative Proteomics Identifies Surfactant-Resistant α-Synuclein in Cerebral Cortex of Parkinsonism-Dementia Complex of Guam but Not Alzheimer’s Disease or Progressive Supranuclear Palsy. The American Journal of Pathology **171(3)**, 993–1002.
192. **Zerr I, Schmitz M, Karch A, Villar‐Piqué A, Kanata E, Golanska E, Díaz‐Lucena D, Karsanidou A, Hermann P, Knipper T, Goebel S, Varges D, Sklaviadis T, Sikorska B, Liberski PP, Santana I, Ferrer I, Zetterberg H, Blennow K, Calero O, Calero M, Ladogana A, Sánchez-Valle R, Baldeiras I, Llorens F** (2018). Cerebrospinal fluid neurofilament light levels in neurodegenerative dementia: Evaluation of diagnostic accuracy in the differential diagnosis of prion diseases. Alzheimer’s and Dementia **14(6)**, 751–763.
193. **Zou J, Chen Z, Liang C, Fu Y, Wei X, Lu J, Pan M, Guo Y, Liao X, Xie H, Wu D, Li M, Liang L, Wang P and Wang Q** (2018). Trefoil Factor 3, Cholinesterase and Homocysteine: Potential Predictors for Parkinson’s Disease Dementia and Vascular Parkinsonism Dementia in Advanced Stage. Aging and Disease **9(1)**, 51-65.
