# Supplementary material for: Systematic review of differentially abundant proteins in people with Lewy body dementia
Source: Acta Neuropsychiatr. 2025 Mar 27;37:e59. doi: 10.1017/neu.2025.15 (PMC13130301; doi:10.1017/neu.2025.15)

Supplementary material-7: Meta-analyses of studies investigating differentially abundant proteins in people with Dementia with Lewy Bodies (DLB)

7.1 Cerebrospinal fluid (CSF)

7.1.1 Tau Protein (TAU, P10636)

7.1.1.1. Meta-analysis of TAU levels in CSF of people with DLB, when compared to healthy controls (HC)

Our random effects meta-analysis showed that CSF TAU levels were significantly higher in people with DLB, when compared to healthy controls (SMD = 0.47; 95%CI 0.36 – 0.58; p < 0.01). We provide the funnel plot below and the forest plot of the meta-analysis in the next page.

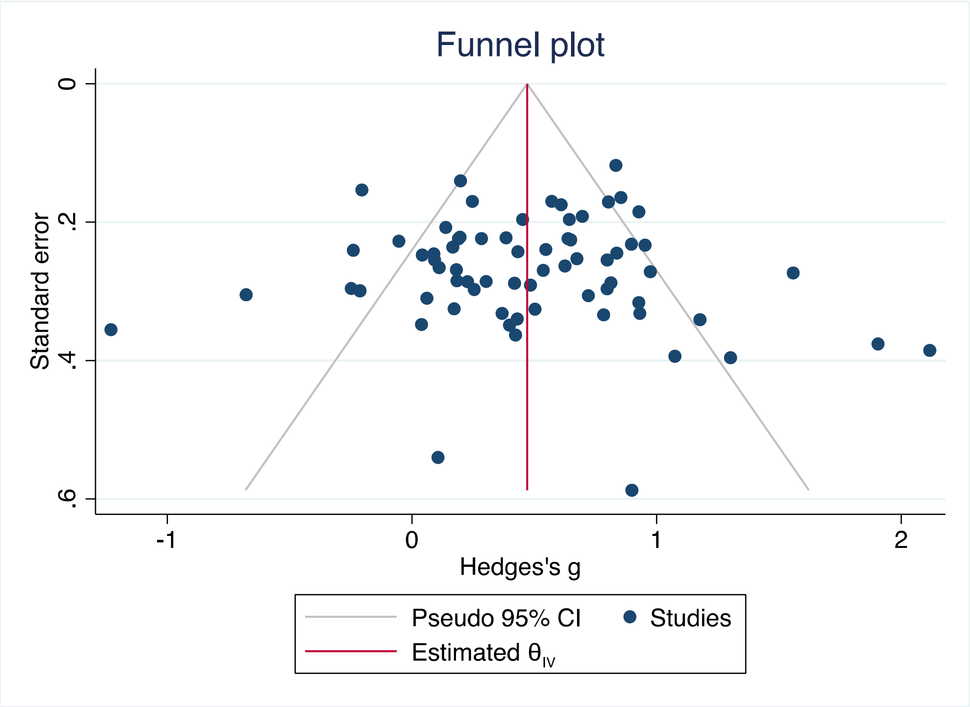

Study	Hedges's g with 95% CI	Weight (%)
Abu-Rumeileh et al. (2018)	0.67 [0.18, 1.17]	1.49
Abu-Rumeileh et al. (2019)	0.64 [0.20, 1.08]	1.59
Alcolea et al. (2021a)	0.80 [0.30, 1.30]	1.48
Bibl et al. (2006)	-1.23 [-1.93, -0.53]	1.16
Bibl et al. (2010)	0.72 [0.12, 1.32]	1.31
Bonomi et al. (2021)	0.50 [-0.14, 1.14]	1.25
Borroni et al. (2008)	0.93 [0.28, 1.58]	1.23
Bostrom et al. (2009)	0.20 [-0.24, 0.63]	1.60
Bougea et al. (2020)	0.63 [0.11, 1.14]	1.45
Bousiges et al. (2018)	0.57 [0.24, 0.90]	1.77
Bousiges et al. (2020)	0.17 [-0.47, 0.81]	1.25
Brockmann et al. (2021)	0.43 [-0.04, 0.91]	1.53
Bruggink et al. (2015)	-0.21 [-0.80, 0.37]	1.33
Bruun et al. (2018)	0.20 [-0.08, 0.47]	1.87
Canaslan et al. (2021)	2.12 [1.36, 2.87]	1.08
Chatterjee et al. (2020)	0.85 [0.53, 1.18]	1.79
Clark et al. (2003)	0.90 [-0.25, 2.05]	0.65
Compta et al. (2016)	0.42 [-0.29, 1.14]	1.14
de Oliveira et al. (2021)	0.18 [-0.35, 0.71]	1.44
Delaby et al. (2020)	0.70 [0.32, 1.07]	1.70
Delaby et al. (2022)	0.83 [0.60, 1.06]	1.93
Engelborghs et al. (2006)	-0.05 [-0.50, 0.39]	1.58
Ewers et al. (2015)	0.17 [-0.30, 0.63]	1.55
Forland et al. (2020)	0.23 [-0.33, 0.79]	1.38
Gmitterova et al. (2020a)	0.19 [-0.25, 0.63]	1.59
Gmitterova et al. (2020b)	0.11 [-0.95, 1.17]	0.73
Gomez-Tortosa (2003)	0.09 [-0.39, 0.57]	1.51
Goossens et al. (2017)	0.11 [-0.41, 0.63]	1.45
Hall et al. (2012)	-0.20 [-0.51, 0.10]	1.83
Kapaki et al. (2013)	0.78 [0.13, 1.44]	1.22
Llorens et al. (2015)	0.09 [-0.41, 0.59]	1.48
Longobardi et al. (2022)	0.30 [-0.26, 0.86]	1.38
Luo et al. (2013)	0.84 [0.36, 1.32]	1.52
Maetzler et al. (2011a)	1.56 [1.02, 2.09]	1.42
Maetzler et al. (2012)	0.38 [-0.05, 0.82]	1.59
Maetzler et al. (2016)	0.93 [0.31, 1.55]	1.28
Mollenhauer et al. (2005a)	0.25 [-0.09, 0.58]	1.77
Mollenhauer et al. (2006a)	1.91 [1.17, 2.64]	1.10
Mollenhauer et al. (2006b)	0.45 [0.07, 0.84]	1.69
Mollenhauer et al. (2007a)	0.90 [0.44, 1.35]	1.56
Mollenhauer et al. (2007b)	1.07 [0.30, 1.85]	1.05
Mollenhauer et al. (2011) (Training)	0.93 [0.56, 1.29]	1.72
Mollenhauer et al. (2011) (Validation)	-0.24 [-0.71, 0.23]	1.53
Morenas-Rodriguez et al. (2019)	0.95 [0.50, 1.41]	1.56
Muller et al. (2015)	-0.21 [-0.80, 0.37]	1.33
Mulugeta et al. (2011a)	0.04 [-0.64, 0.72]	1.18
Mulugeta et al. (2011b)	0.40 [-0.29, 1.08]	1.18
Nielsen et al. (2007a)	0.04 [-0.44, 0.53]	1.51
Nielsen et al. (2014)	0.65 [0.21, 1.09]	1.58
Ohrfelt et al. (2009)	0.48 [-0.09, 1.05]	1.36
Olsson et al. (2019)	0.14 [-0.27, 0.54]	1.65
Parnetti et al. (2008)	1.18 [0.51, 1.85]	1.20
Paterson et al. (2018)	0.18 [-0.37, 0.74]	1.38
Quadalti et al. (2021)	0.61 [0.27, 0.95]	1.76
Schulz et al. (2021)	0.54 [0.01, 1.07]	1.43
Slaets et al. (2014)	0.43 [-0.24, 1.10]	1.21
Spies et al. (2010)	0.42 [-0.15, 0.98]	1.37
Timmer et al. (2015)	-0.68 [-1.28, -0.08]	1.32
Trotti et al. (2021)	-0.25 [-0.83, 0.33]	1.35
van Steenoven et al. (2018)	0.64 [0.26, 1.03]	1.69
van Steenoven et al. (2019)	0.97 [0.44, 1.51]	1.43
van Steenoven et al. (2020) (Cohort 1)	0.06 [-0.55, 0.67]	1.30
van Steenoven et al. (2020) (Cohort 2)	1.30 [0.53, 2.08]	1.05
van Steenoven et al. (2020) (Cohort 3)	0.55 [0.08, 1.02]	1.54
Vanderstichele et al. (2006)	0.80 [0.47, 1.14]	1.77
Vranova et al. (2014)	0.37 [-0.28, 1.02]	1.23
Wennstrom et al. (2013)	0.81 [0.25, 1.38]	1.37
Wennstrom et al. (2015a)	0.80 [0.22, 1.38]	1.34
Wennstrom et al. (2015b)	0.28 [-0.15, 0.72]	1.59
Willemse et al. (2018)	0.25 [-0.33, 0.84]	1.34
**Overall**	0.47 [0.36, 0.58]	

Heterogeneity: $\tau^2 = 0.16$, $I^2 = 72.54\%$, $H^2 = 3.64$

Test of $\theta_i = \theta_j$: Q(69) = 232.19, p = 0.00

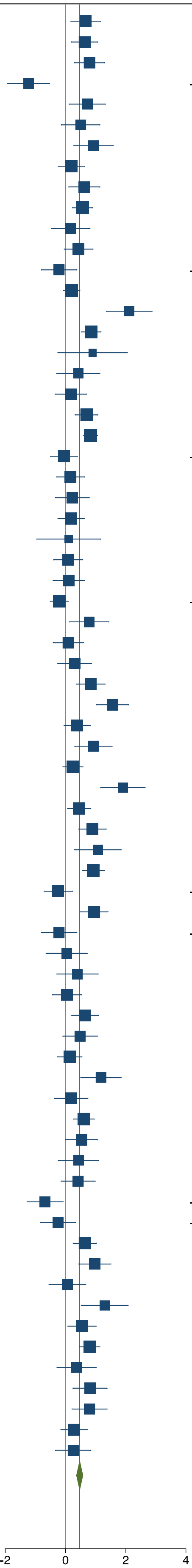

7.1.1.2. Meta-analysis of TAU levels in CSF of people with DLB, when compared to people with other dementia.

Our random effects meta-analysis showed that CSF TAU levels were significantly lower in people with DLB, when compared to people with other dementia (SMD =-0.89; 95%CI -1.00 – -0.78; p<0.01). We provide the funnel plot below and the forest plot of the meta-analysis in the next page.

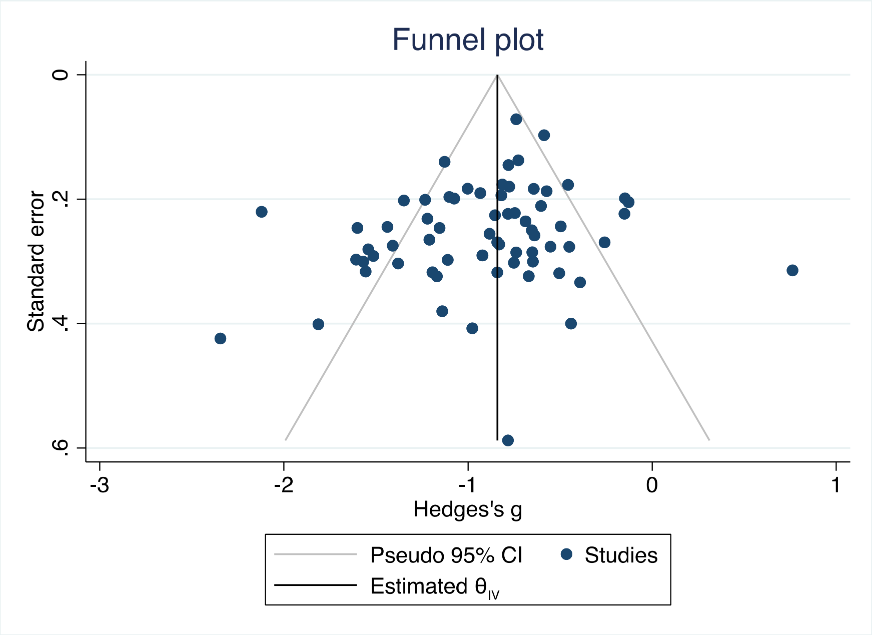

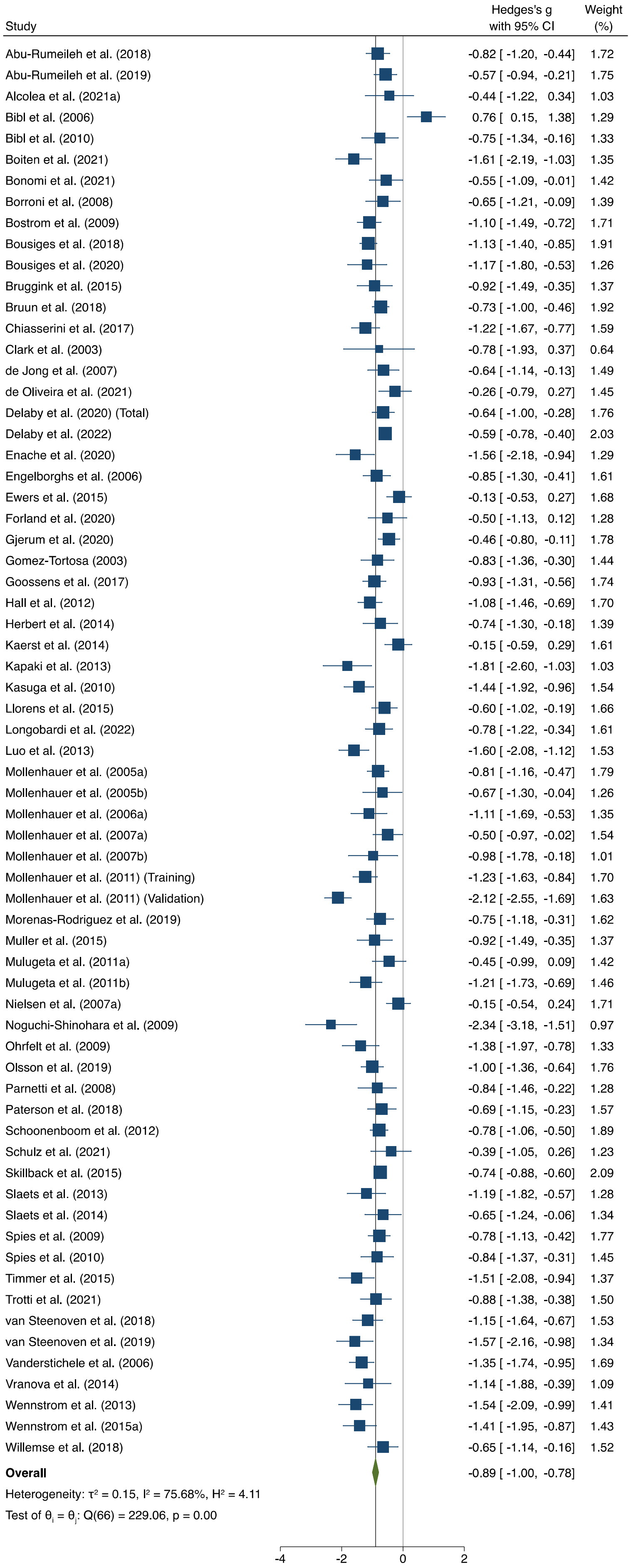
Study
Hedges's g with 95% CI
Weight (%)
Abu-Rumeileh et al. (2018) -0.82 [-1.20, -0.44] 1.72
Abu-Rumeileh et al. (2019) -0.57 [-0.94, -0.21] 1.75
Alcolea et al. (2021a) -0.44 [-1.22, 0.34] 1.03
Bibl et al. (2006) 0.76 [0.15, 1.38] 1.29
Bibl et al. (2010) -0.75 [-1.34, -0.16] 1.33
Boiten et al. (2021) -1.61 [-2.19, -1.03] 1.35
Bonomi et al. (2021) -0.55 [-1.09, -0.01] 1.42
Borroni et al. (2008) -0.65 [-1.21, -0.09] 1.39
Bostrom et al. (2009) -1.10 [-1.49, -0.72] 1.71
Bousiges et al. (2018) -1.13 [-1.40, -0.85] 1.91
Bousiges et al. (2020) -1.17 [-1.80, -0.53] 1.26
Bruggink et al. (2015) -0.92 [-1.49, -0.35] 1.37
Bruun et al. (2018) -0.73 [-1.00, -0.46] 1.92
Chiasserini et al. (2017) -1.22 [-1.67, -0.77] 1.59
Clark et al. (2003) -0.78 [-1.93, 0.37] 0.64
de Jong et al. (2007) -0.64 [-1.14, -0.13] 1.49
de Oliveira et al. (2021) -0.26 [-0.79, 0.27] 1.45
Delaby et al. (2020) (Total) -0.64 [-1.00, -0.28] 1.76
Delaby et al. (2022) -0.59 [-0.78, -0.40] 2.03
Enache et al. (2020) -1.56 [-2.18, -0.94] 1.29
Engelborghs et al. (2006) -0.85 [-1.30, -0.41] 1.61
Ewers et al. (2015) -0.13 [-0.53, 0.27] 1.68
Forland et al. (2020) -0.50 [-1.13, 0.12] 1.28
Gjerum et al. (2020) -0.46 [-0.80, -0.11] 1.78
Gomez-Tortosa (2003) -0.83 [-1.36, -0.30] 1.44
Goossens et al. (2017) -0.93 [-1.31, -0.56] 1.74
Hall et al. (2012) -1.08 [-1.46, -0.69] 1.70
Herbert et al. (2014) -0.74 [-1.30, -0.18] 1.39
Kaerst et al. (2014) -0.15 [-0.59, 0.29] 1.61
Kapaki et al. (2013) -1.81 [-2.60, -1.03] 1.03
Kasuga et al. (2010) -1.44 [-1.92, -0.96] 1.54
Llorens et al. (2015) -0.60 [-1.02, -0.19] 1.66
Longobardi et al. (2022) -0.78 [-1.22, -0.34] 1.61
Luo et al. (2013) -1.60 [-2.08, -1.12] 1.53
Mollenhauer et al. (2005a) -0.81 [-1.16, -0.47] 1.79
Mollenhauer et al. (2005b) -0.67 [-1.30, -0.04] 1.26
Mollenhauer et al. (2006a) -1.11 [-1.69, -0.53] 1.35
Mollenhauer et al. (2007a) -0.50 [-0.97, -0.02] 1.54
Mollenhauer et al. (2007b) -0.98 [-1.78, -0.18] 1.01
Mollenhauer et al. (2011) (Training) -1.23 [-1.63, -0.84] 1.70
Mollenhauer et al. (2011) (Validation) -2.12 [-2.55, -1.69] 1.63
Morenas-Rodriguez et al. (2019) -0.75 [-1.18, -0.31] 1.62
Muller et al. (2015) -0.92 [-1.49, -0.35] 1.37
Mulugeta et al. (2011a) -0.45 [-0.99, 0.09] 1.42
Mulugeta et al. (2011b) -1.21 [-1.73, -0.69] 1.46
Nielsen et al. (2007a) -0.15 [-0.54, 0.24] 1.71
Noguchi-Shinohara et al. (2009) -2.34 [-3.18, -1.51] 0.97
Ohrfelt et al. (2009) -1.38 [-1.97, -0.78] 1.33
Olsson et al. (2019) -1.00 [-1.36, -0.64] 1.76
Parnetti et al. (2008) -0.84 [-1.46, -0.22] 1.28
Paterson et al. (2018) -0.69 [-1.15, -0.23] 1.57
Schoonenboom et al. (2012) -0.78 [-1.06, -0.50] 1.89
Schulz et al. (2021) -0.39 [-1.05, 0.26] 1.23
Skillback et al. (2015) -0.74 [-0.88, -0.60] 2.09
Slaets et al. (2013) -1.19 [-1.82, -0.57] 1.28
Slaets et al. (2014) -0.65 [-1.24, -0.06] 1.34
Spies et al. (2009) -0.78 [-1.13, -0.42] 1.77
Spies et al. (2010) -0.84 [-1.37, -0.31] 1.45
Timmer et al. (2015) -1.51 [-2.08, -0.94] 1.37
Trotti et al. (2021) -0.88 [-1.38, -0.38] 1.50
van Steenoven et al. (2018) -1.15 [-1.64, -0.67] 1.53
van Steenoven et al. (2019) -1.57 [-2.16, -0.98] 1.34
Vanderstichele et al. (2006) -1.35 [-1.74, -0.95] 1.69
Vranova et al. (2014) -1.14 [-1.88, -0.39] 1.09
Wennstrom et al. (2013) -1.54 [-2.09, -0.99] 1.41
Wennstrom et al. (2015a) -1.41 [-1.95, -0.87] 1.43
Willemse et al. (2018) -0.65 [-1.14, -0.16] 1.52
Overall -0.89 [-1.00, -0.78]
Heterogeneity: τ² = 0.15, I² = 75.68%, H² = 4.11
Test of θi = θj: Q(66) = 229.06, p = 0.00
-4
-2
0
2

7.1.1.3. Meta-analysis of TAU levels in CSF of people with DLB, when compared to people with Alzheimer's disease (AD).

Our random effects meta-analysis showed that CSF TAU levels were significantly lower in people with DLB, when compared to those of people with AD (SMD =-1.02; 95%CI -1.15 – -0.90; p<0.01).We provide the funnel plot below and the forest plot of the meta-analysis in the next page.

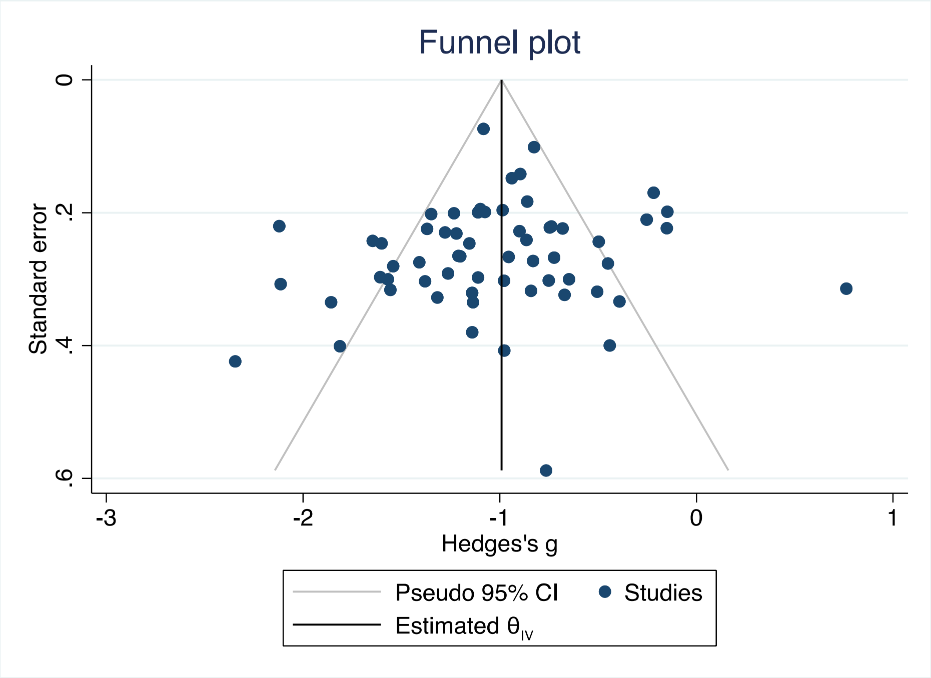

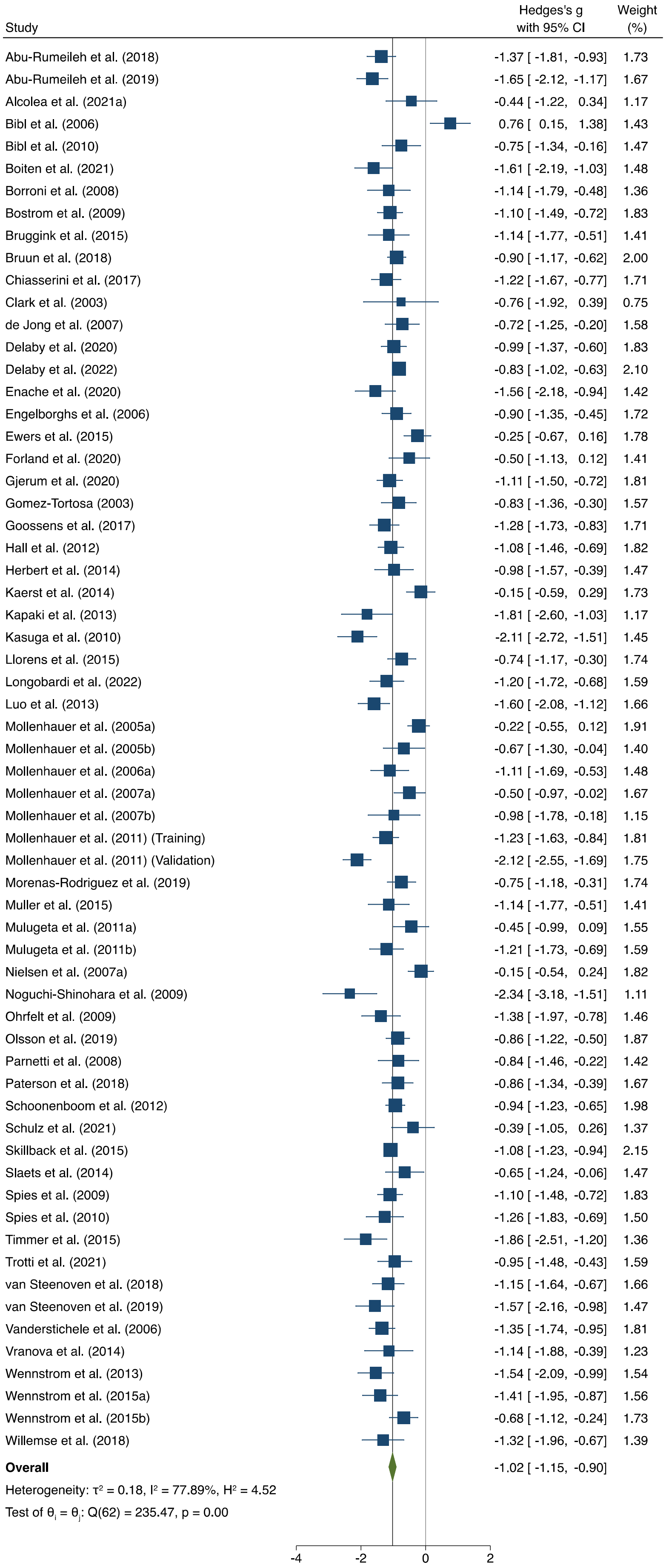
Study
Hedges's g with 95% CI
Weight (%)
Abu-Rumeileh et al. (2018) -1.37 [-1.81, -0.93] 1.73
Abu-Rumeileh et al. (2019) -1.65 [-2.12, -1.17] 1.67
Alcolea et al. (2021a) -0.44 [-1.22, 0.34] 1.17
Bibl et al. (2006) 0.76 [0.15, 1.38] 1.43
Bibl et al. (2010) -0.75 [-1.34, -0.16] 1.47
Boiten et al. (2021) -1.61 [-2.19, -1.03] 1.48
Borroni et al. (2008) -1.14 [-1.79, -0.48] 1.36
Bostrom et al. (2009) -1.10 [-1.49, -0.72] 1.83
Bruggink et al. (2015) -1.14 [-1.77, -0.51] 1.41
Bruun et al. (2018) -0.90 [-1.17, -0.62] 2.00
Chiasserini et al. (2017) -1.22 [-1.67, -0.77] 1.71
Clark et al. (2003) -0.76 [-1.92, 0.39] 0.75
de Jong et al. (2007) -0.72 [-1.25, -0.20] 1.58
Delaby et al. (2020) -0.99 [-1.37, -0.60] 1.83
Delaby et al. (2022) -0.83 [-1.02, -0.63] 2.10
Enache et al. (2020) -1.56 [-2.18, -0.94] 1.42
Engelborghs et al. (2006) -0.90 [-1.35, -0.45] 1.72
Ewers et al. (2015) -0.25 [-0.67, 0.16] 1.78
Forland et al. (2020) -0.50 [-1.13, 0.12] 1.41
Gjerum et al. (2020) -1.11 [-1.50, -0.72] 1.81
Gomez-Tortosa (2003) -0.83 [-1.36, -0.30] 1.57
Goossens et al. (2017) -1.28 [-1.73, -0.83] 1.71
Hall et al. (2012) -1.08 [-1.46, -0.69] 1.82
Herbert et al. (2014) -0.98 [-1.57, -0.39] 1.47
Kaerst et al. (2014) -0.15 [-0.59, 0.29] 1.73
Kapaki et al. (2013) -1.81 [-2.60, -1.03] 1.17
Kasuga et al. (2010) -2.11 [-2.72, -1.51] 1.45
Llorens et al. (2015) -0.74 [-1.17, -0.30] 1.74
Longobardi et al. (2022) -1.20 [-1.72, -0.68] 1.59
Luo et al. (2013) -1.60 [-2.08, -1.12] 1.66
Mollenhauer et al. (2005a) -0.22 [-0.55, 0.12] 1.91
Mollenhauer et al. (2005b) -0.67 [-1.30, -0.04] 1.40
Mollenhauer et al. (2006a) -1.11 [-1.69, -0.53] 1.48
Mollenhauer et al. (2007a) -0.50 [-0.97, -0.02] 1.67
Mollenhauer et al. (2007b) -0.98 [-1.78, -0.18] 1.15
Mollenhauer et al. (2011) (Training) -1.23 [-1.63, -0.84] 1.81
Mollenhauer et al. (2011) (Validation) -2.12 [-2.55, -1.69] 1.75
Morenas-Rodriguez et al. (2019) -0.75 [-1.18, -0.31] 1.74
Muller et al. (2015) -1.14 [-1.77, -0.51] 1.41
Mulugeta et al. (2011a) -0.45 [-0.99, 0.09] 1.55
Mulugeta et al. (2011b) -1.21 [-1.73, -0.69] 1.59
Nielsen et al. (2007a) -0.15 [-0.54, 0.24] 1.82
Noguchi-Shinohara et al. (2009) -2.34 [-3.18, -1.51] 1.11
Ohrfelt et al. (2009) -1.38 [-1.97, -0.78] 1.46
Olsson et al. (2019) -0.86 [-1.22, -0.50] 1.87
Parnetti et al. (2008) -0.84 [-1.46, -0.22] 1.42
Paterson et al. (2018) -0.86 [-1.34, -0.39] 1.67
Schoonenboom et al. (2012) -0.94 [-1.23, -0.65] 1.98
Schulz et al. (2021) -0.39 [-1.05, 0.26] 1.37
Skillback et al. (2015) -1.08 [-1.23, -0.94] 2.15
Slaets et al. (2014) -0.65 [-1.24, -0.06] 1.47
Spies et al. (2009) -1.10 [-1.48, -0.72] 1.83
Spies et al. (2010) -1.26 [-1.83, -0.69] 1.50
Timmer et al. (2015) -1.86 [-2.51, -1.20] 1.36
Trotti et al. (2021) -0.95 [-1.48, -0.43] 1.59
van Steenoven et al. (2018) -1.15 [-1.64, -0.67] 1.66
van Steenoven et al. (2019) -1.57 [-2.16, -0.98] 1.47
Vanderstichele et al. (2006) -1.35 [-1.74, -0.95] 1.81
Vranova et al. (2014) -1.14 [-1.88, -0.39] 1.23
Wennstrom et al. (2013) -1.54 [-2.09, -0.99] 1.54
Wennstrom et al. (2015a) -1.41 [-1.95, -0.87] 1.56
Wennstrom et al. (2015b) -0.68 [-1.12, -0.24] 1.73
Willemse et al. (2018) -1.32 [-1.96, -0.67] 1.39
Overall -1.02 [-1.15, -0.90]
Heterogeneity: τ² = 0.18, I² = 77.89%, H² = 4.52
Test of θi = θj: Q(62) = 235.47, p = 0.00
-4
-2
0
2

7.1.2 Alpha Synuclein (SYUA, P37840)

7.1.2.1. Meta-analysis of SYUA levels in CSF of people with DLB, when compared to healthy controls (HC)

Our random effects meta-analysis showed that CSF SYUA levels were significantly lower in people with DLB, when compared to those of HC (SMD = -0.39; 95%CI -0.70 - -0.07; p=0.02). We provide the funnel plot below and the forest plot of the meta-analysis is presented in Figure 2-A.

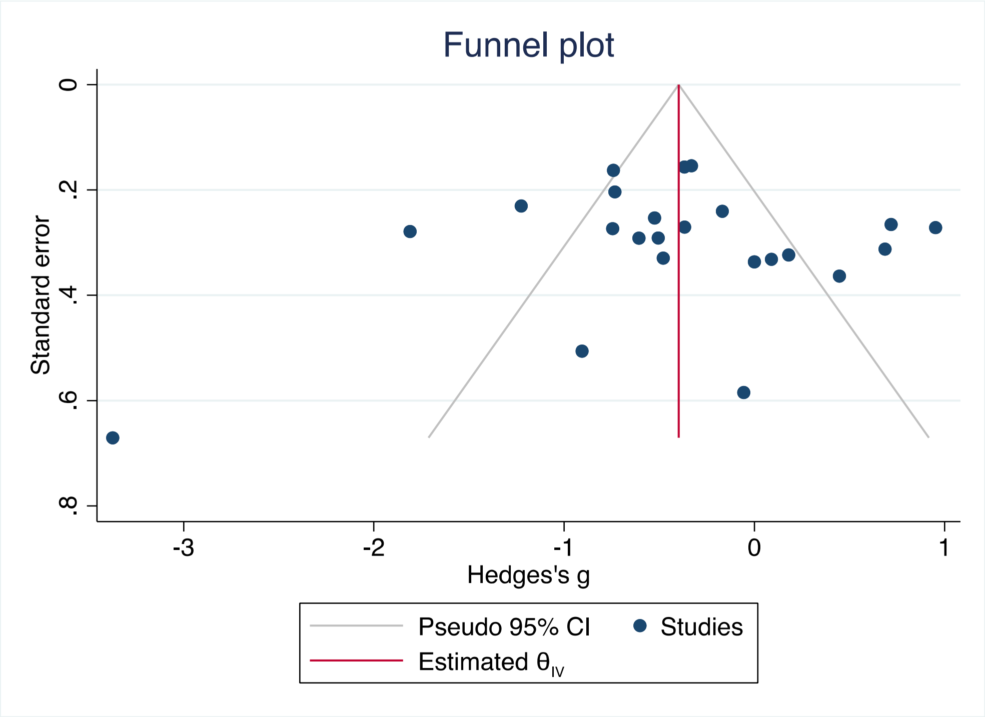

7.1.2.2. Meta-analysis of SYUA levels in CSF of people with DLB, when compared to people with other dementia.

Our random effects meta-analysis showed that CSF SYUA levels were significantly lower in people with DLB, when compared to people with other dementia (SMD =-0.37; 95%CI -0.69 - -0.05; p=0.02). We provide the funnel plot below and the forest plot of the meta-analysis is presented in Figure 2-B.

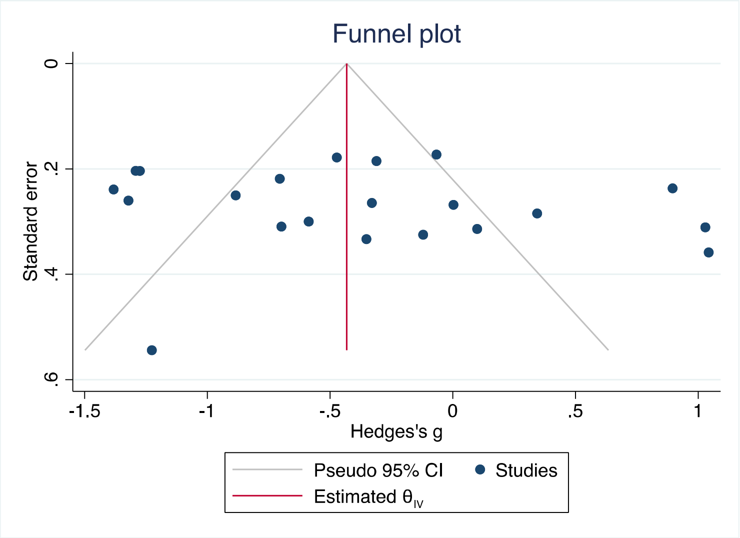

7.1.2.3. Meta-analysis of SYUA levels in CSF of people with DLB, when compared to people with Alzheimer's disease (AD).

Our random effects meta-analysis showed that CSF SYUA levels were significantly lower in people with DLB, when compared to those of people with AD (SMD = -0.36; 95%CI -0.68 - -0.04; p=0.03). We provide the funnel and forest plots of the meta-analysis below.

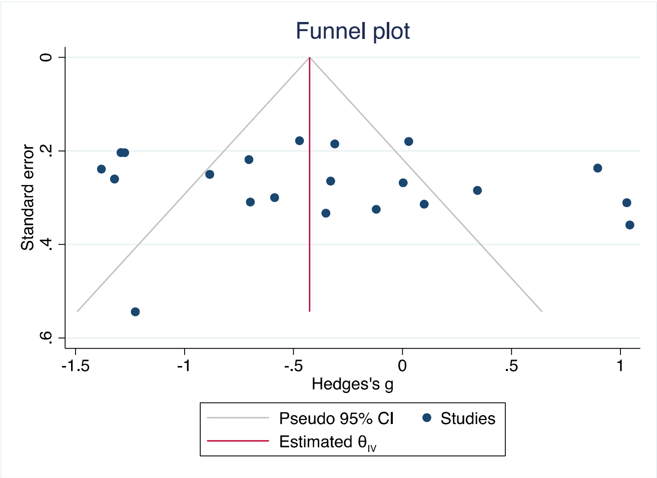

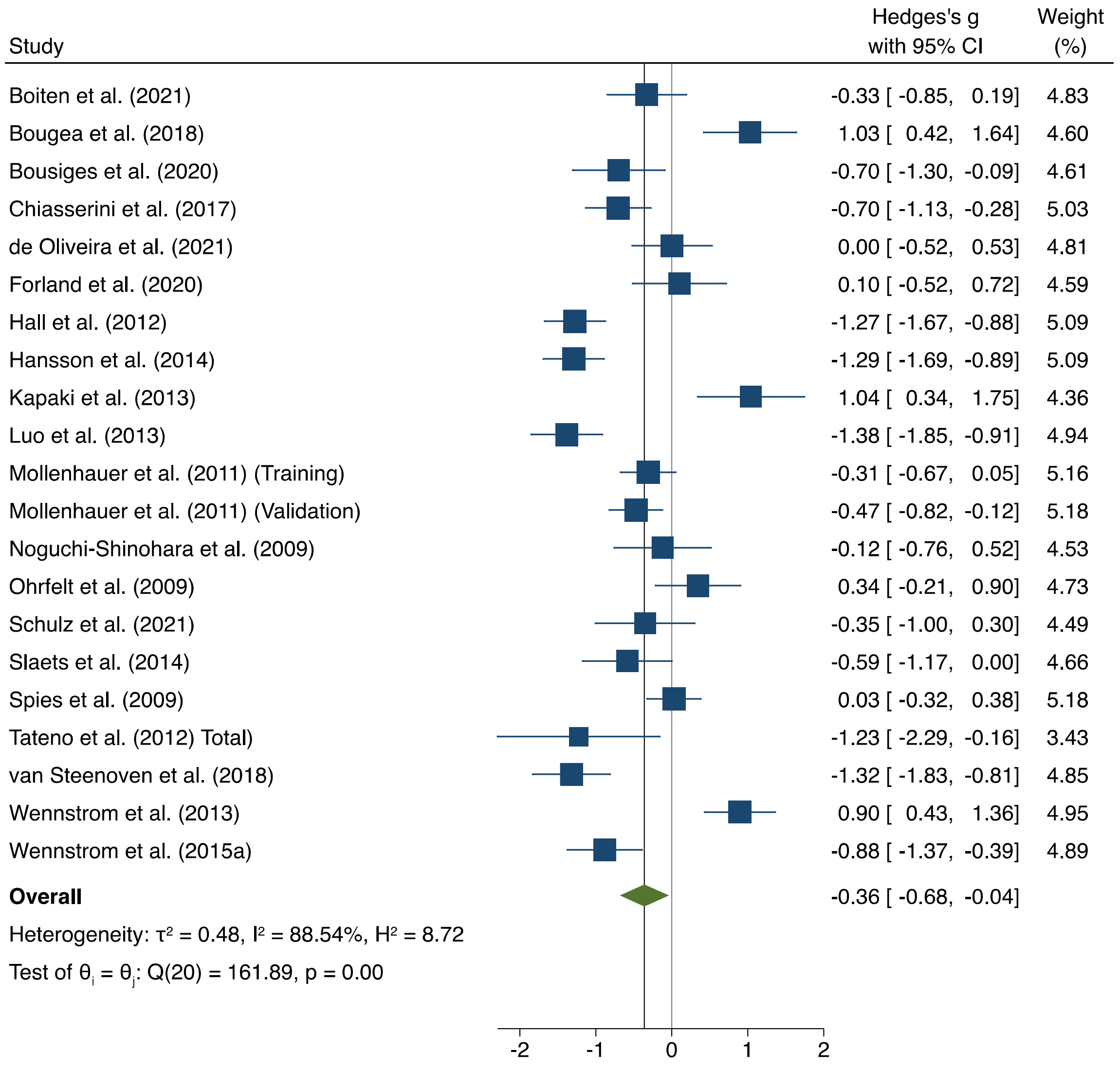

7.1.3 Neurofilament light polypeptide (NFL, P07196)

7.1.3.1. Meta-analysis of NFL levels in CSF of people with DLB, when compared to HC

Our random effects meta-analysis showed that CSF NFL levels were significantly higher in people with DLB, when compared to those of HC (SMD=1.19; 95%CI 0.55 – 1.83; $p<0.01$). We provide the funnel and forest plots of the meta-analysis below.

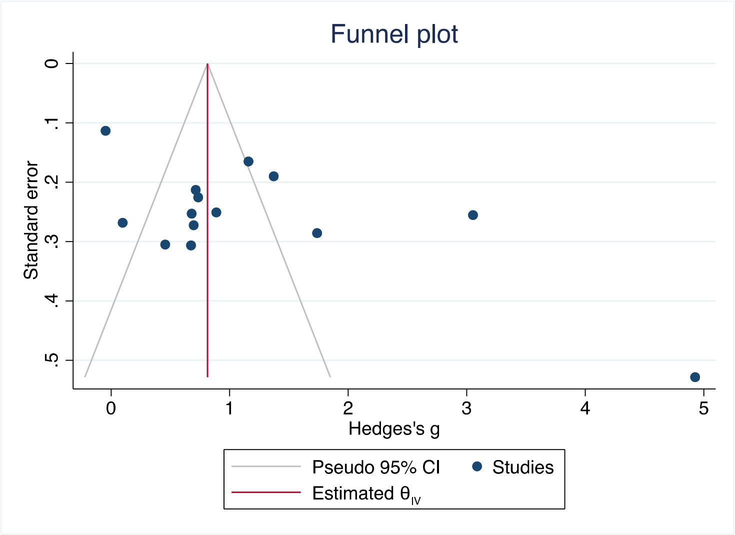

Funnel plot
Standard error
0
.1
.2
.3
.4
.5
0
1
2
3
4
5
Hedges's g
Pseudo 95% CI
Studies
Estimated θ_{IV}

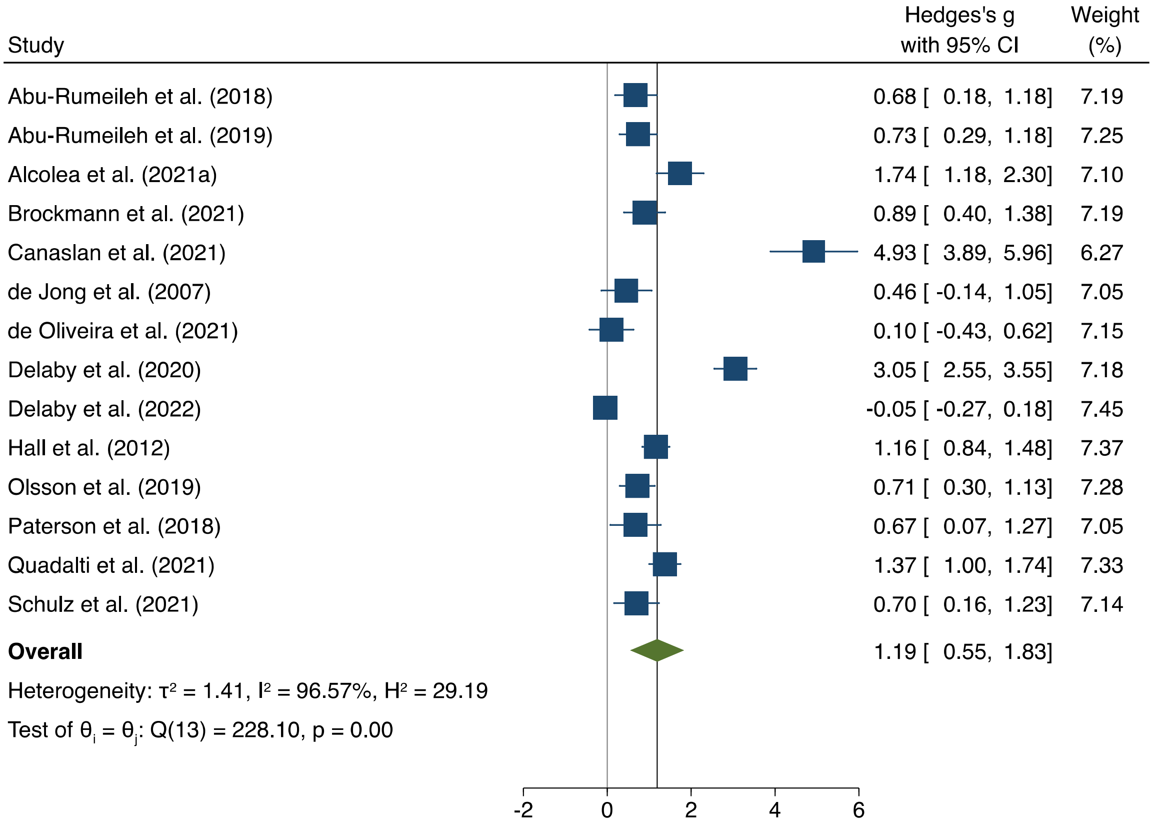

Study
Hedges's g with 95% CI
Weight (%)
Abu-Rumeileh et al. (2018) 0.68 [0.18, 1.18] 7.19
Abu-Rumeileh et al. (2019) 0.73 [0.29, 1.18] 7.25
Alcolea et al. (2021a) 1.74 [1.18, 2.30] 7.10
Brockmann et al. (2021) 0.89 [0.40, 1.38] 7.19
Canaslan et al. (2021) 4.93 [3.89, 5.96] 6.27
de Jong et al. (2007) 0.46 [-0.14, 1.05] 7.05
de Oliveira et al. (2021) 0.10 [-0.43, 0.62] 7.15
Delaby et al. (2020) 3.05 [2.55, 3.55] 7.18
Delaby et al. (2022) -0.05 [-0.27, 0.18] 7.45
Hall et al. (2012) 1.16 [0.84, 1.48] 7.37
Olsson et al. (2019) 0.71 [0.30, 1.13] 7.28
Paterson et al. (2018) 0.67 [0.07, 1.27] 7.05
Quadalti et al. (2021) 1.37 [1.00, 1.74] 7.33
Schulz et al. (2021) 0.70 [0.16, 1.23] 7.14
Overall 1.19 [0.55, 1.83]
Heterogeneity: τ^2 = 1.41, I^2 = 96.57%, H^2 = 29.19
Test of $\theta_i = \theta_j$: Q(13) = 228.10, p = 0.00
-2
0
2
4
6

7.1.3.2. Meta-analysis of NFL levels in CSF of people with DLB, when compared to people with other dementia.

Our random effects meta-analysis showed that CSF NFL levels were significantly lower in people with DLB, when compared to people with other dementia (SMD= -0.32; 95%CI -0.53 - -0.12; p<0.01). We provide the funnel and forest plots of the meta-analysis below.

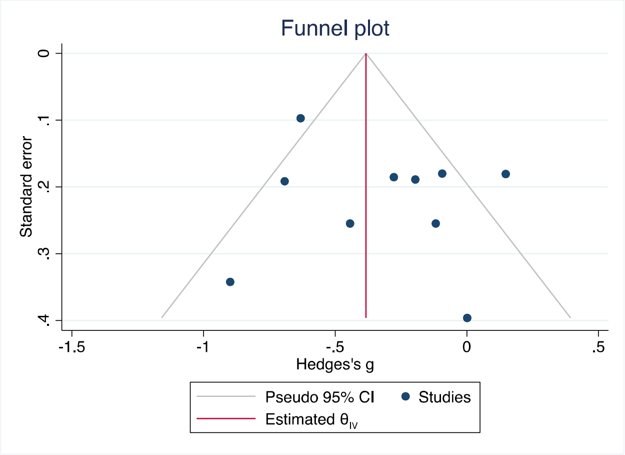

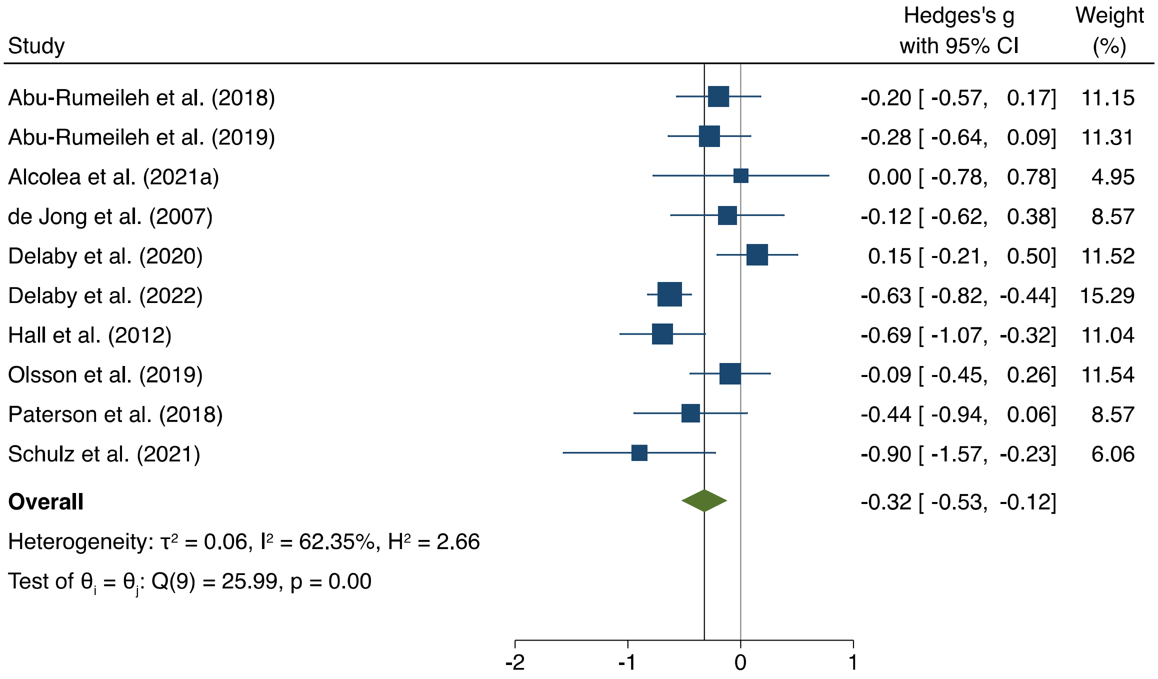

7.1.3.3. Meta-analysis of NFL levels in CSF of people with DLB, when compared to people with AD.

Our random effects meta-analysis showed that CSF NFL levels in people with DLB were not significantly different from those of people with AD (SMD= -0.13; 95%CI -0.41 – 0.15; p=0.38). We provide the funnel and forest plots of the meta-analysis below.

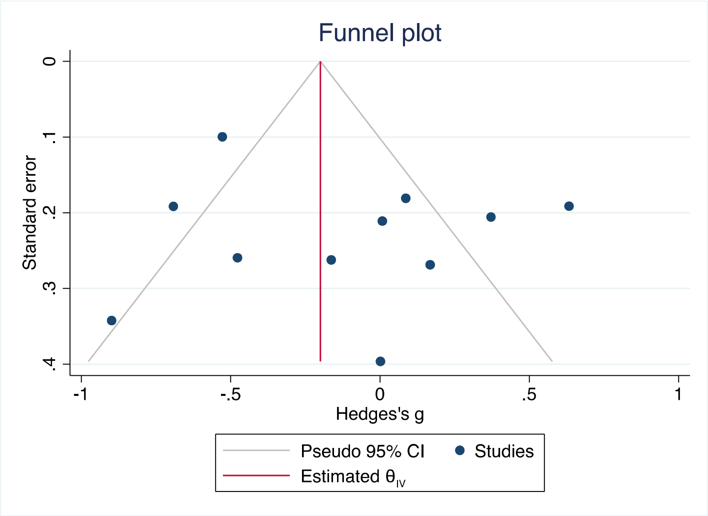

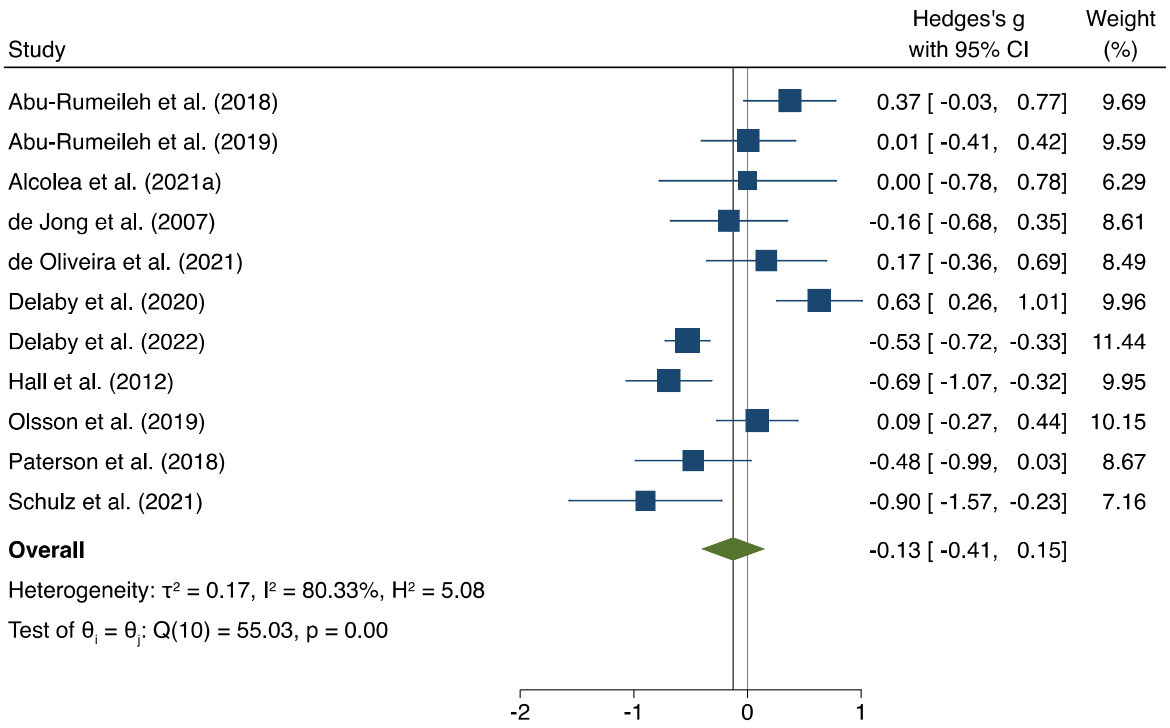

7.1.4 Chitinase-3-like protein 1 (CHI3L1, P36222)

7.1.4.1. Meta-analysis of CHI3L1 levels in CSF of people with DLB, when compared to healthy controls

Our random effects meta-analysis showed that CSF CHI3L1 levels were significantly higher in people with DLB, when compared to those of HC (SMD=0.53; 95%CI 0.09 – 0.97; p=0.02). We provide the funnel and forest plots of the meta-analysis below.

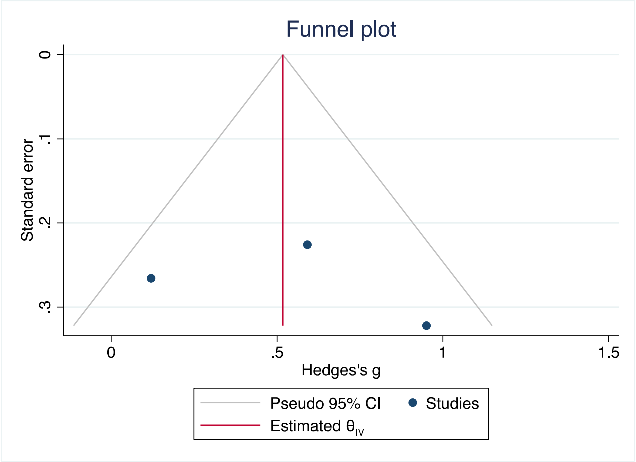

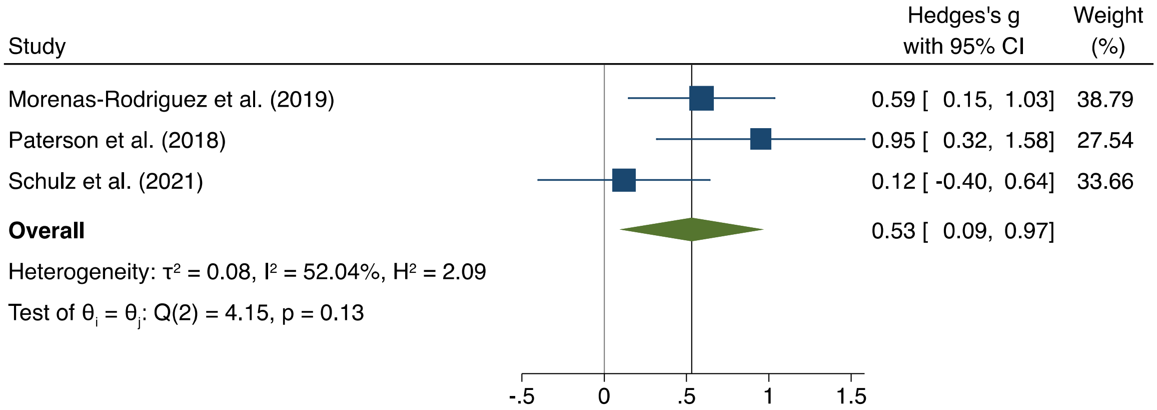

7.1.4.2. Meta-analysis of CHI3L1 levels in CSF of people with DLB, when compared to people with AD.

Our random effects meta-analysis showed that CSF CHI3L1 levels in people with DLB were not significantly different from those of people with AD (SMD= -0.37; 95%CI -0.79 – 0.06; p=0.09). We provide the funnel and forest plots of the meta-analysis below.

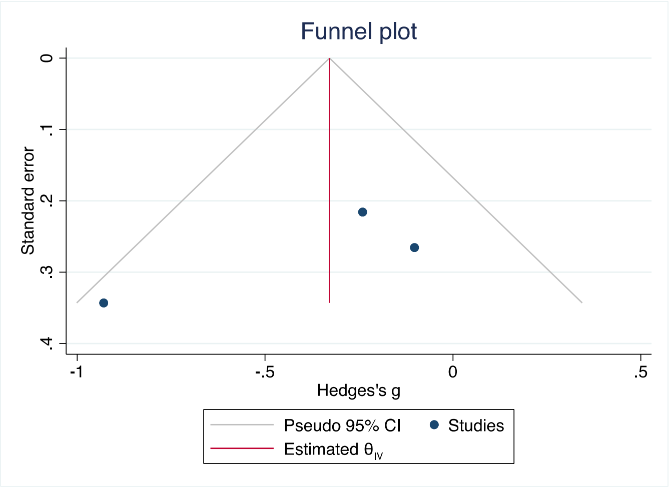

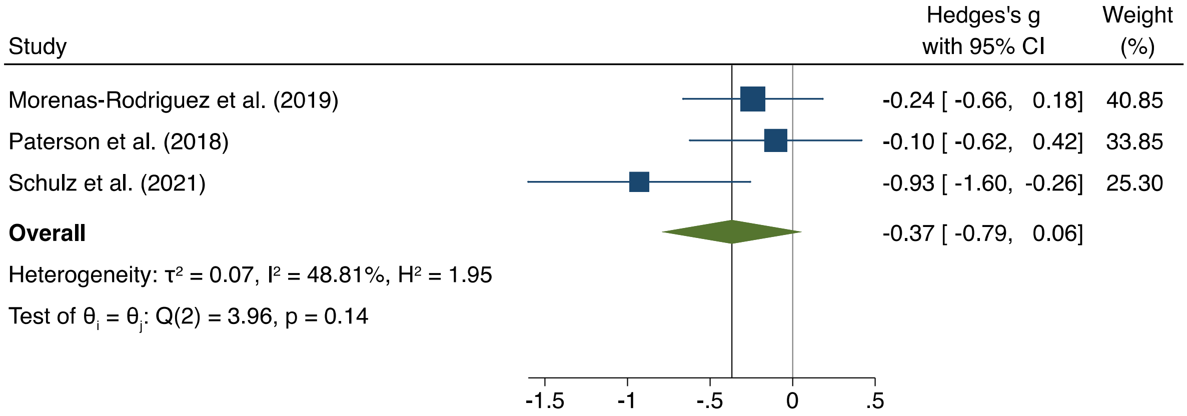

7.1.5 Glial fibrillary acidic protein (GFAP, P14136)

7.1.5.1. Meta-analysis of GFAP levels in CSF of people with DLB, when compared to healthy controls

Our random effects meta-analysis showed that CSF GFAP levels were significantly higher in people with DLB, when compared to those of HC (SMD=0.99; 95%CI 0.56 – 1.41; p<0.01). We provide the funnel and forest plots of the meta-analysis below.

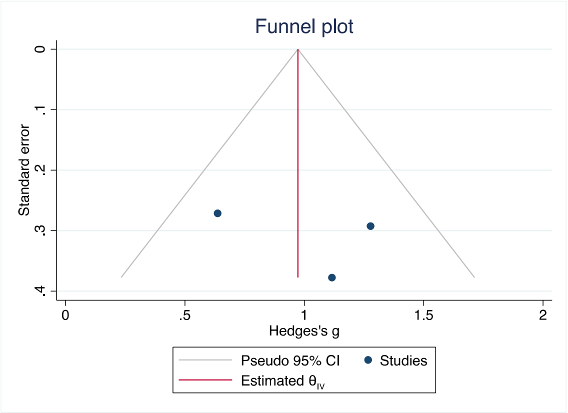

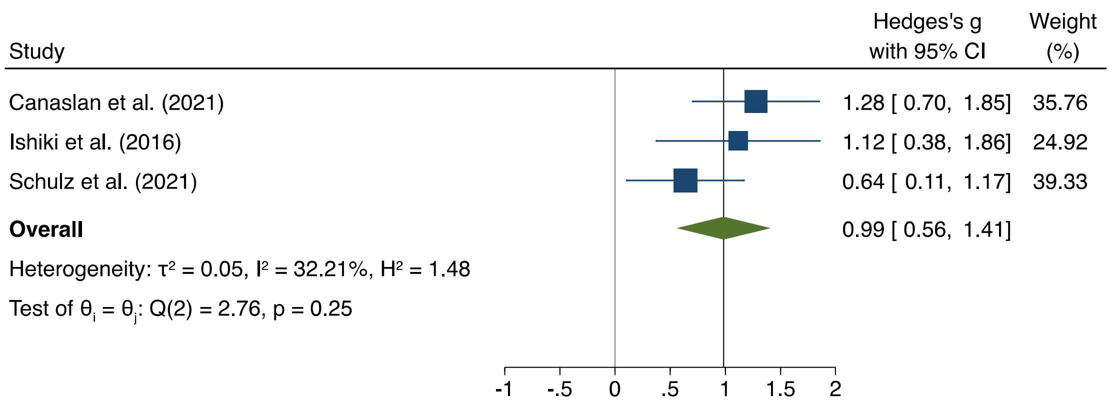

7.1.6 Fatty Acid-binding protein, heart (FABPH, P05413)

7.1.6.1. Meta-analysis of FABPH levels in CSF of people with DLB, when compared to people with AD

Our random effects meta-analysis showed that CSF FABPH levels in people with DLB were not significantly different from those of people with AD (SMD= -0.51; 95%CI -1.32 - 0.31; p=0.22). We provide the funnel and forest plots of the meta-analysis below.

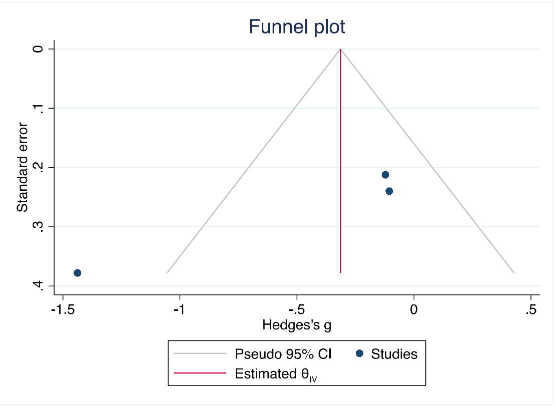

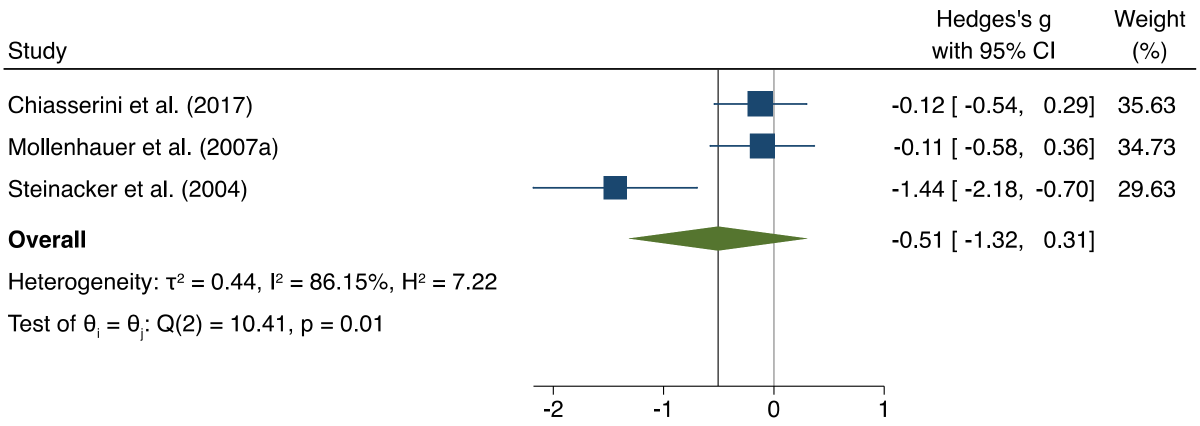

7.1.7 S100B (P04271)

7.1.7.1. Meta-analysis of S100B levels in CSF of people with DLB, when compared to people with healthy controls

Our random effects meta-analysis showed that CSF S100B levels in people with DLB were not significantly different from those of healthy controls (SMD =0.53; 95%CI -0.36 – 1.42; p=0.24). We provide the funnel and forest plots of the meta-analysis below.

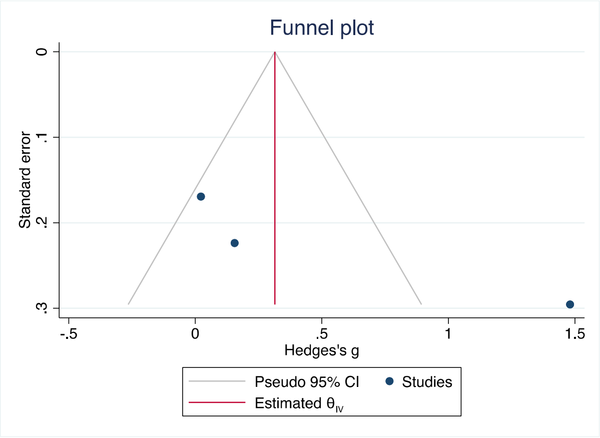

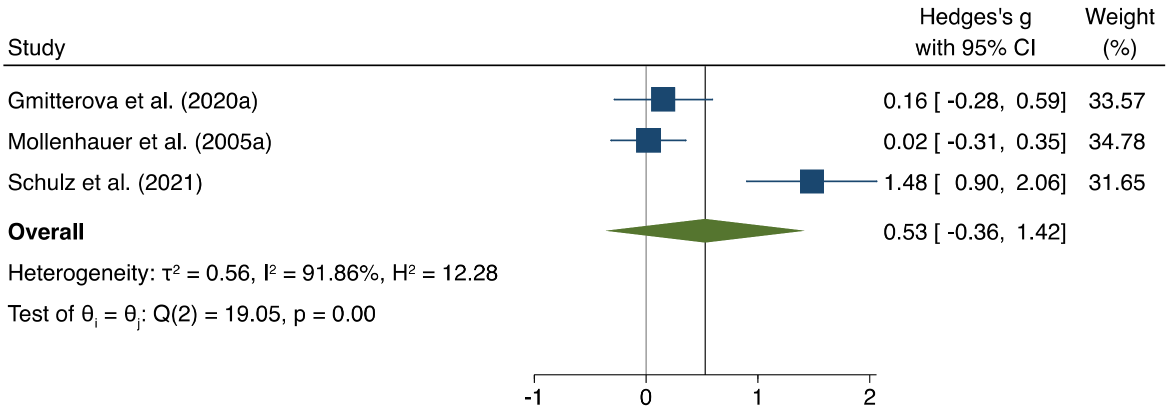

7.2. Post-mortem brain tissue

7.2.1. Alpha Synuclein (SYUA, P37840)

7.2.1.1. Meta-analysis of SYUA levels in brain tissue of people with DLB, when compared to healthy controls

Our random effects meta-analysis showed that SYUA levels in post-mortem brain tissue of people with DLB were not significantly different from those of healthy controls (SMD =1.66; 95%CI -1.58 – 4.90; p=0.32). We provide the funnel and forest plots of the meta-analysis below.

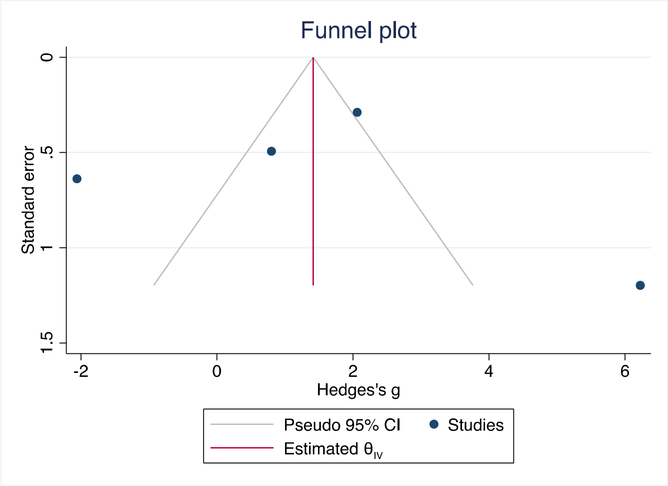

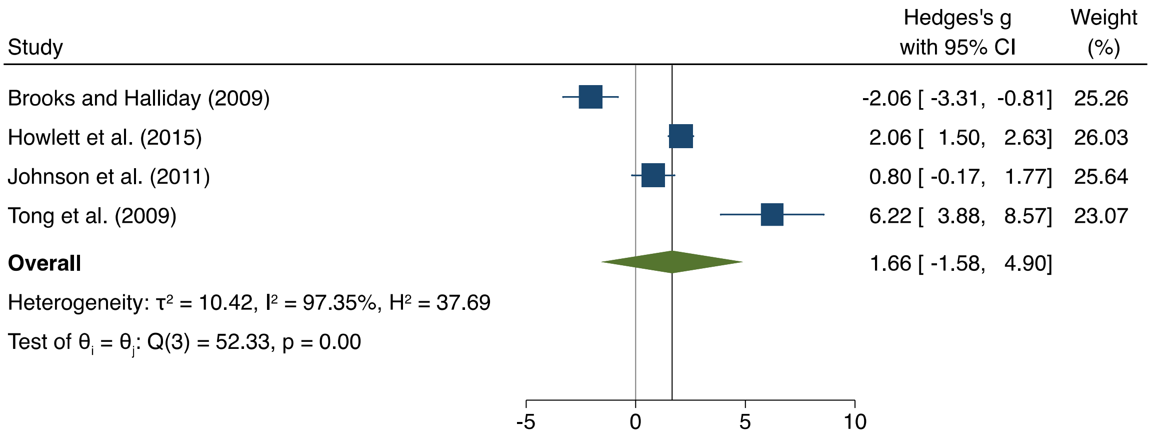

7.2.1.2. Meta-analysis of SYUA levels in brain tissue of people with DLB, when compared to people with other dementia

Our random effects meta-analysis showed that SYUA levels in post-mortem brain tissue of people with DLB were not significantly different from those of people with other dementia

(SMD=2.16; 95%CI -0.43 - 4.74; p=0.10). We provide the funnel and forest plots of the meta-analysis below.

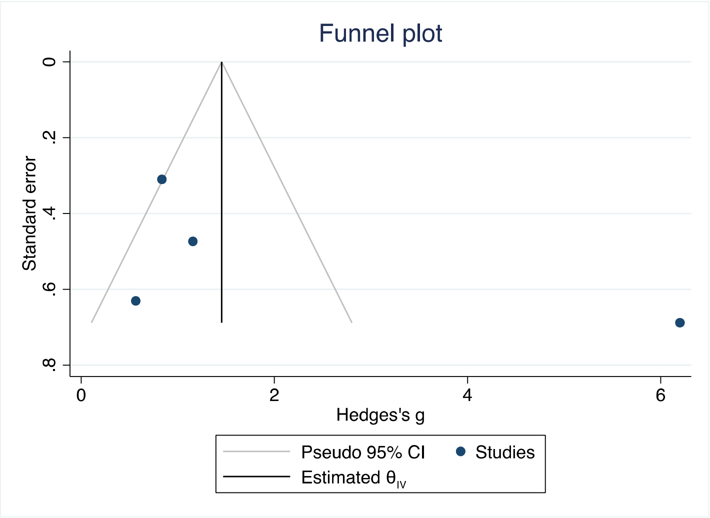

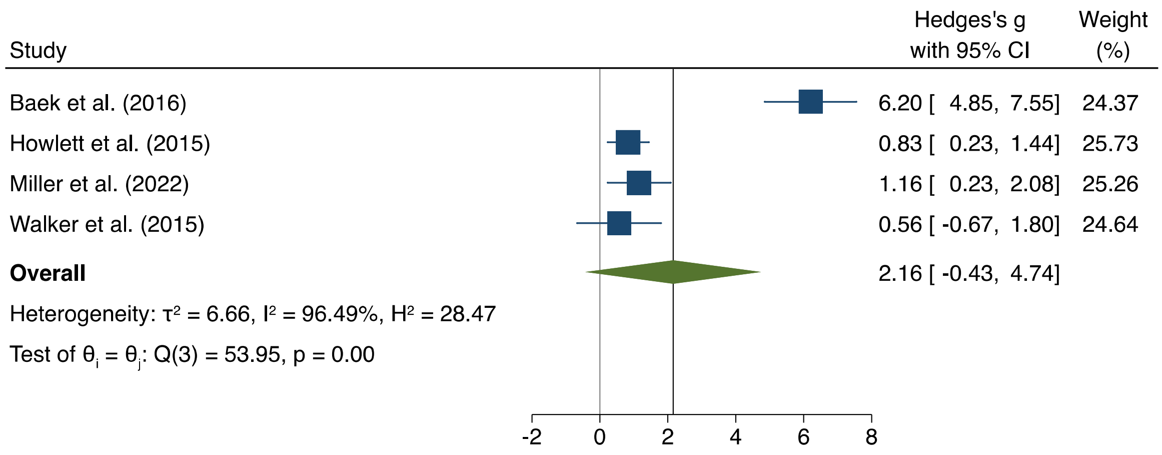

7.2.1.3. Meta-analysis of SYUA levels in brain tissue of people with DLB, when compared to people with AD

Our random effects meta-analysis showed that SYUA levels in post-mortem brain tissue of people with DLB were not significantly different from those of people with AD (SMD = 3.35; 95%CI -2.02 - 8.72; p=0.22). We provide the funnel and forest plots of the meta-analysis below.

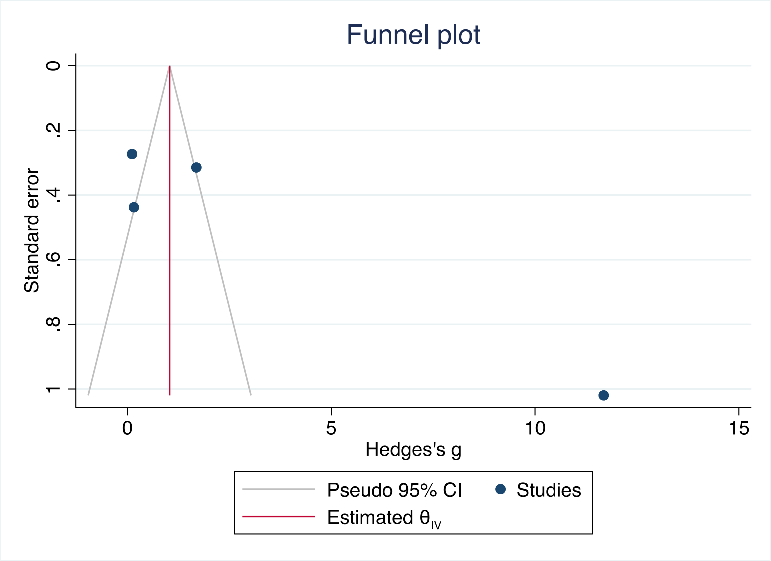

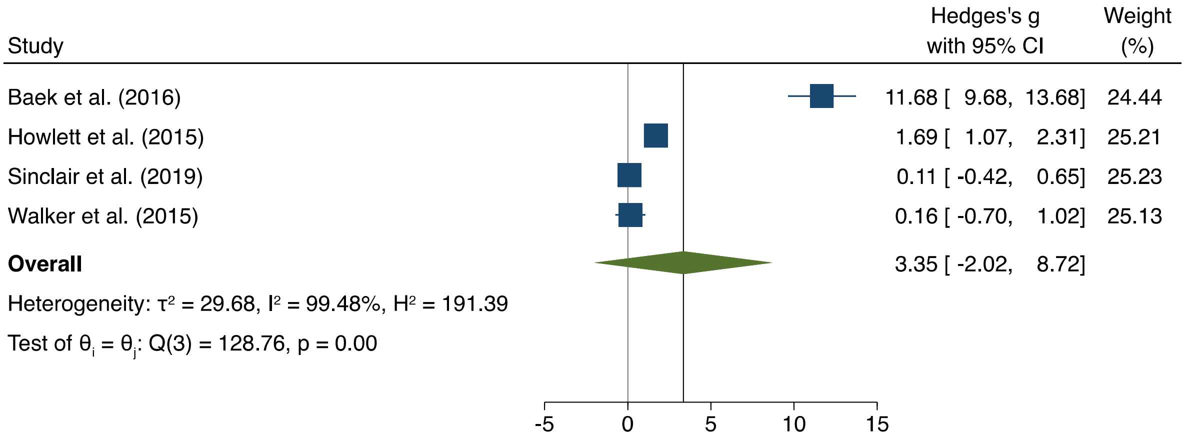

7.2.2 Choline-O-Acetyltransferase (CLAT, P28329)

7.2.2.1. Meta-analysis of CLAT levels in brain tissue of people with DLB, when compared to healthy controls

Our random effects meta-analysis showed that CLAT expression levels in post-mortem brain tissue were significantly lower in people with DLB, when compared to healthy controls (SMD= -3.64; 95%CI -6.75 - -0.54; p=0.02). We provide the funnel plot below and the forest plot of the meta-analysis in the next page.

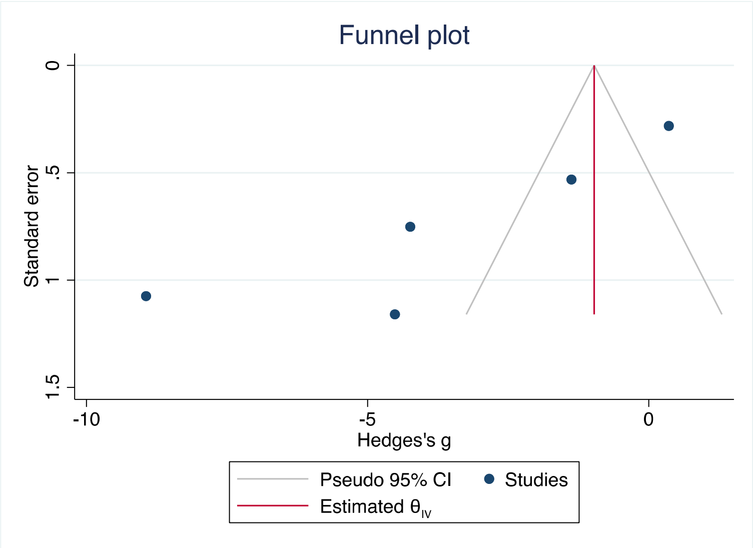

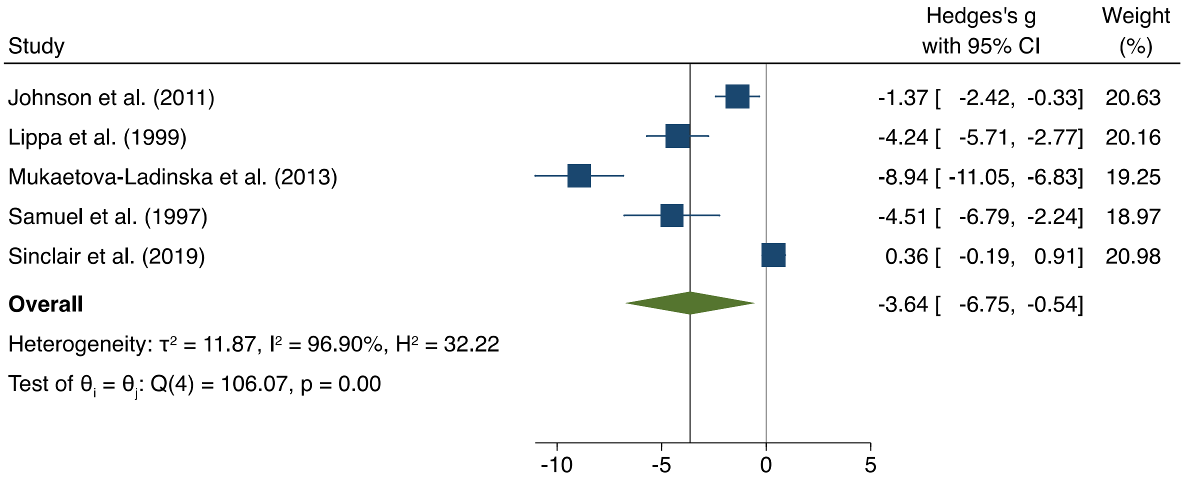

7.2.2.2. Meta-analysis of CLAT levels in brain tissue of people with DLB, when compared to people with AD

Our random effects meta-analysis showed that CLAT levels in post-mortem brain tissue of people with DLB were not significantly different from those of people with AD (SMD= -1.17; 95%CI -2.36 – 0.03; p=0.06). We provide the funnel and forest plots of the meta-analysis below.

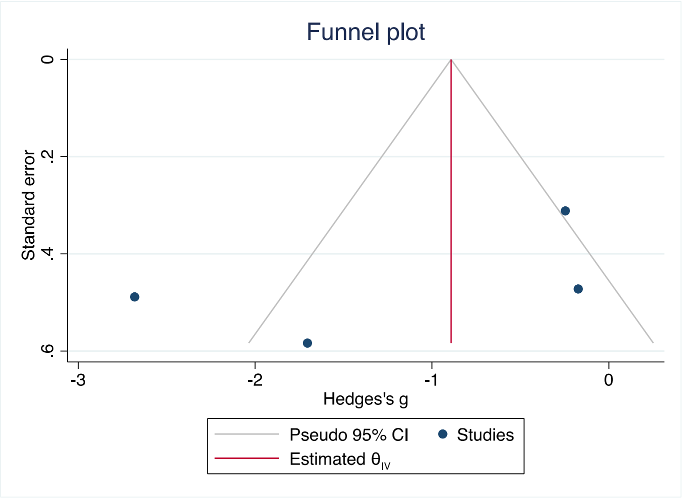
Funnel plot
Standard error
0
.2
.4
.6
-3
-2
-1
0
Hedges's g
Pseudo 95% CI
Studies
Estimated θIV

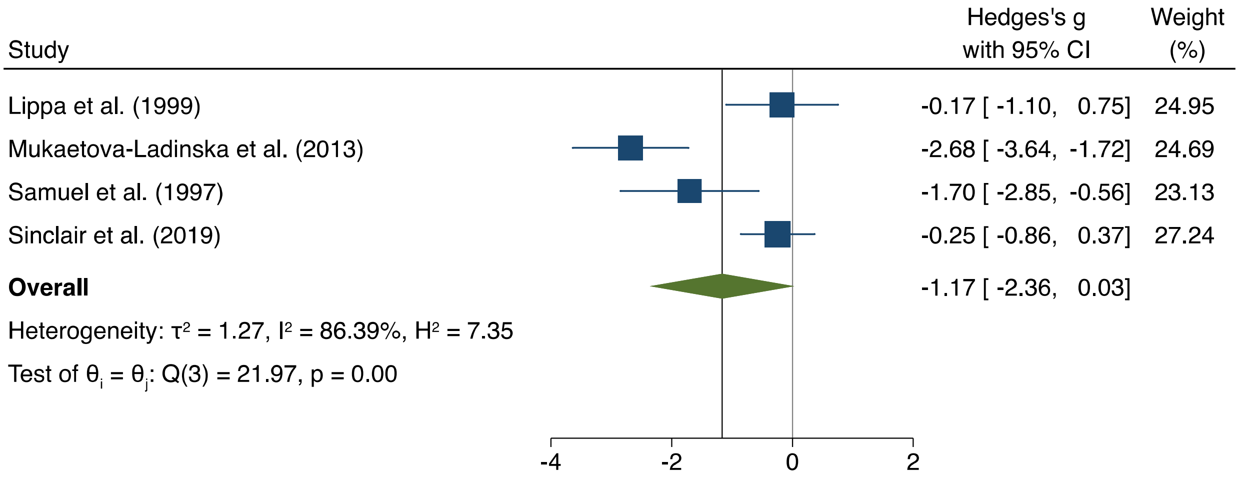
Study
Hedges's g with 95% CI
Weight (%)
Lippa et al. (1999)
-0.17 [-1.10, 0.75] 24.95
Mukaetova-Ladinska et al. (2013)
-2.68 [-3.64, -1.72] 24.69
Samuel et al. (1997)
-1.70 [-2.85, -0.56] 23.13
Sinclair et al. (2019)
-0.25 [-0.86, 0.37] 27.24
Overall
-1.17 [-2.36, 0.03]
Heterogeneity: τ² = 1.27, I² = 86.39%, H² = 7.35
Test of θi = θj: Q(3) = 21.97, p = 0.00
-4
-2
0
2

7.3. Blood-based

7.3.1. Alpha Synuclein (SYUA, P37840)

7.3.1.1. Meta-analysis of SYUA levels in plasma or serum of people with DLB, when compared to healthy controls

Our random effects meta-analysis showed that SYUA levels in plasma or serum of people with DLB were not significantly different from those of healthy controls (SMD =0.12; 95%CI -1.11 – 1.36; p=0.84). We provide the funnel and forest plots of the meta-analysis below.

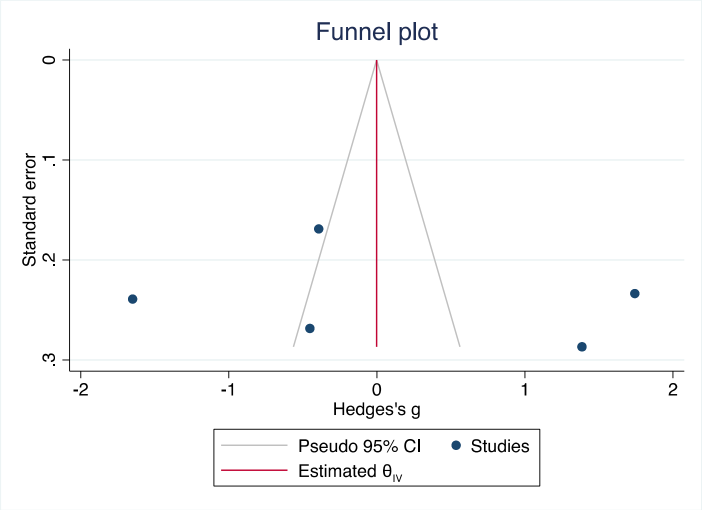

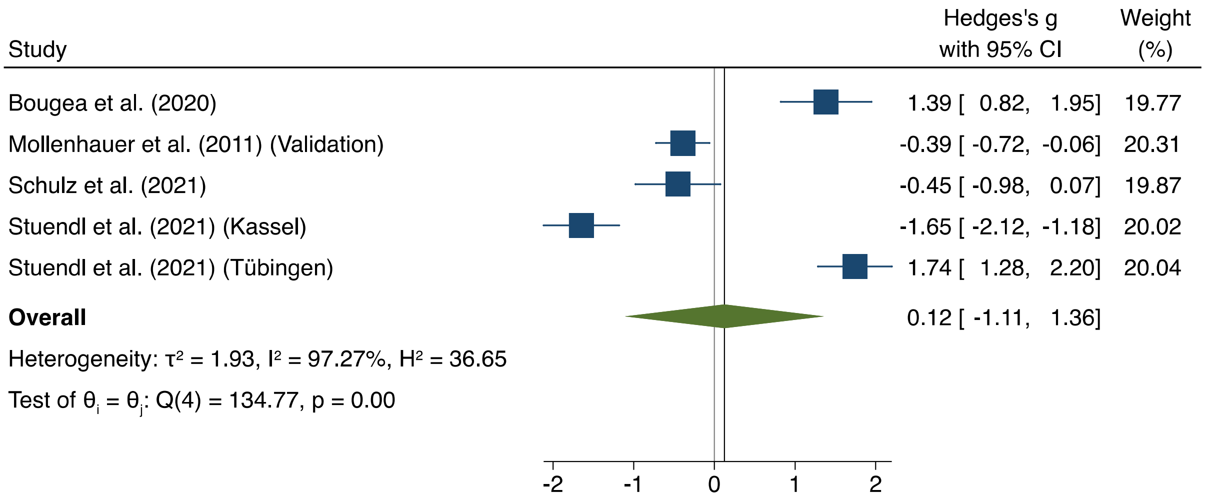

Supplement: Farr et al. supplementary material 8 — Farr et al. supplementary material [file S0924270825000158sup008.pdf]
