# Supplementary material for: Systematic review of differentially abundant proteins in people with Lewy body dementia
Source: Acta Neuropsychiatr. 2025 Mar 27;37:e59. doi: 10.1017/neu.2025.15 (PMC13130301; doi:10.1017/neu.2025.15)

Supplementary material-8: Meta-analyses of studies investigating differentially abundant proteins in people with Parkinson's Disease Dementia (PDD)

8.1 Cerebrospinal fluid (CSF)

8.1.1. Tau Protein (TAU, P10636)

8.1.1.1. Meta-analysis of TAU levels in CSF of people with PDD, when compared to healthy controls (HC)

Our random effects meta-analysis showed that CSF TAU levels were significantly higher in people with PDD, when compared to healthy controls (SMD = 0.27; 95%CI 0.02–0.53; p=0.03). We provide the funnel plot below and the forest plot of the meta-analysis is presented in Figure 3-A.

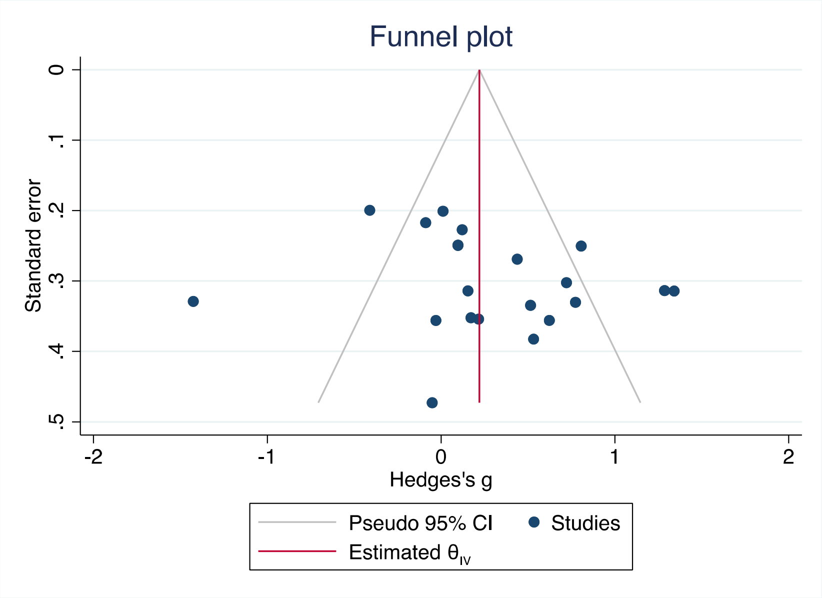

8.1.1.2. Meta-analysis of TAU levels in CSF of people with PDD, when compared to people with other dementia.

Our random effects meta-analysis showed that CSF TAU levels were significantly lower in people with PDD, when compared to people with other dementia (SMD = -0.94; 95%CI -1.17 – -0.72; p<0.01). We provide the funnel plot below and the forest plot of the meta-analysis is presented in Figure 3-B.

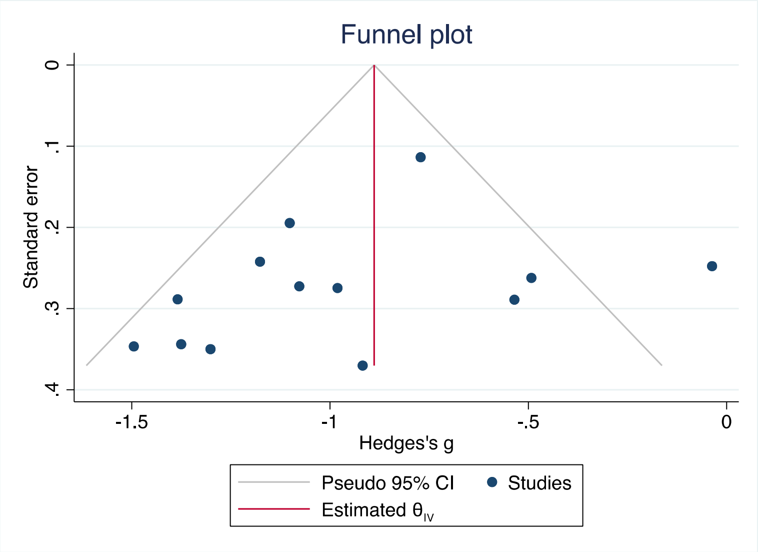

8.1.1.3. Meta-analysis of TAU levels in CSF of people with PDD, when compared to people with Alzheimer's disease (AD).

Our random effects meta-analysis showed that CSF TAU levels were significantly lower in people with PDD, when compared to those of people with AD (SMD = -0.99; 95%CI -1.19 – -0.79; p<0.01). We provide the funnel and forest plots of the meta-analysis below.

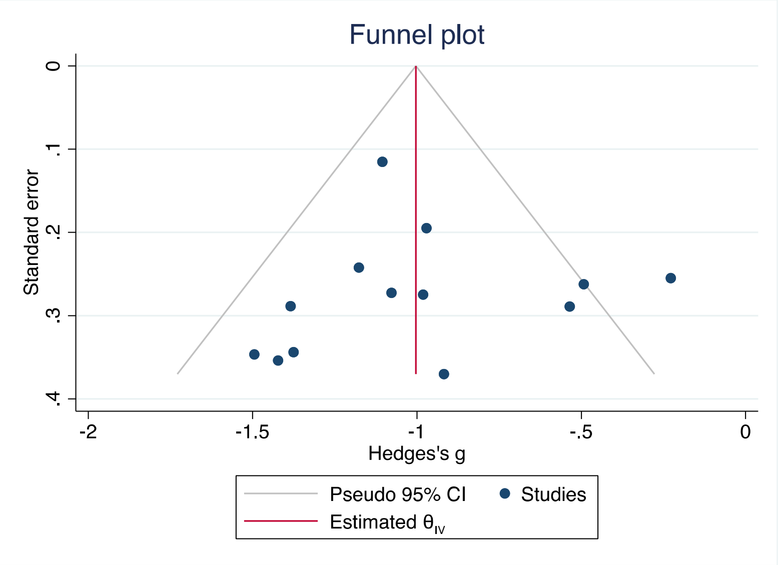

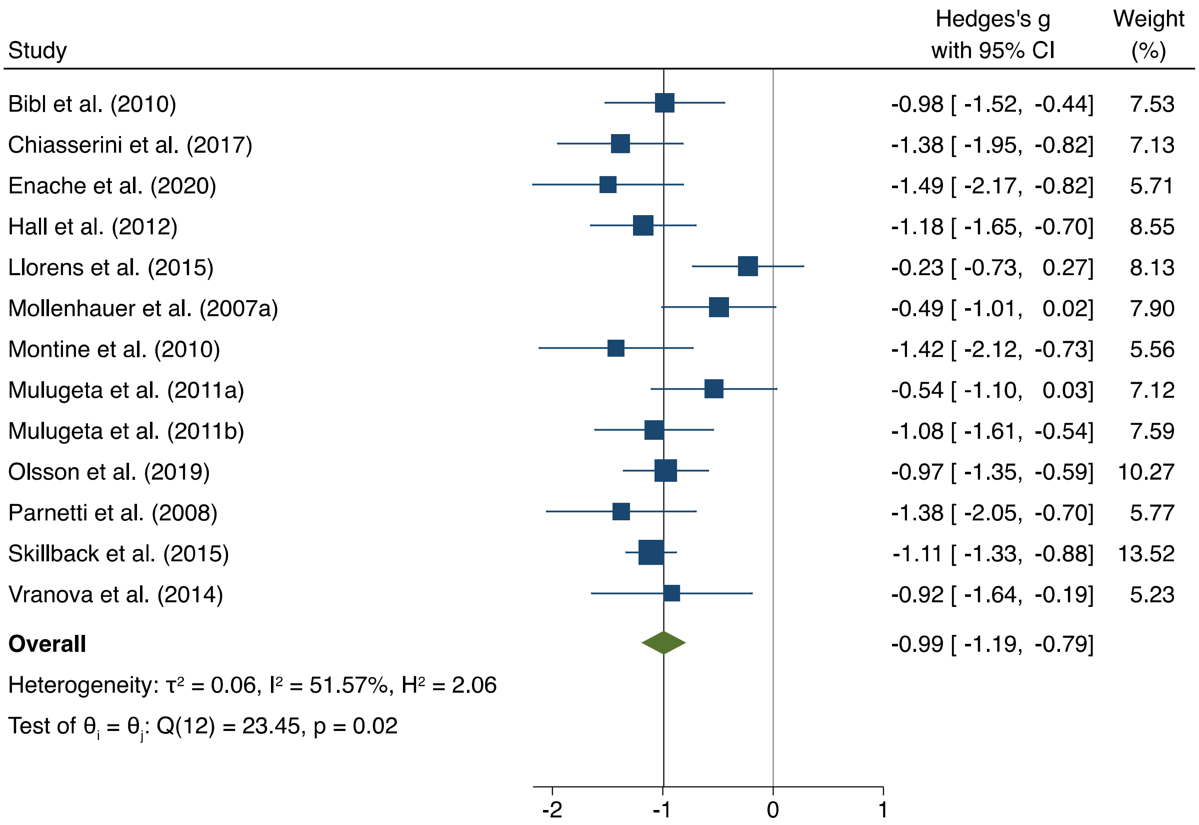

8.1.2. Alpha Synuclein (SYUA, P37840)

8.1.2.1. Meta-analysis of SYUA levels in CSF of people with PDD, when compared to healthy controls

Our random effects meta-analysis showed that CSF SYUA levels in people with PDD were not significantly different from healthy controls (SMD = -0.34; 95%CI -0.67 – 0.00; p=0.05). We provide the funnel and forest plots of the meta-analysis below.

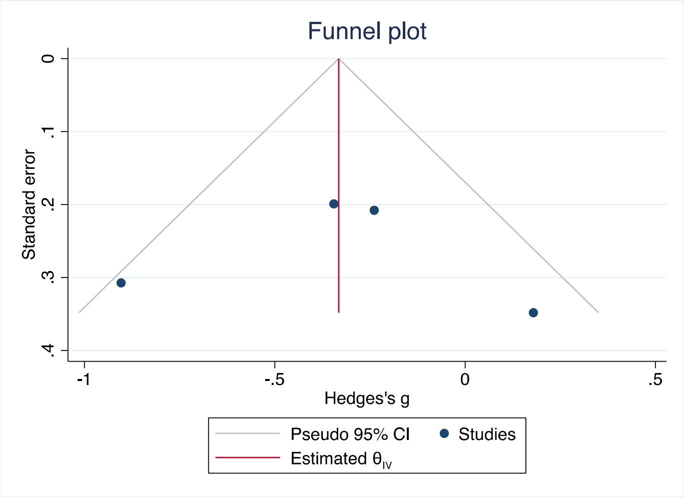

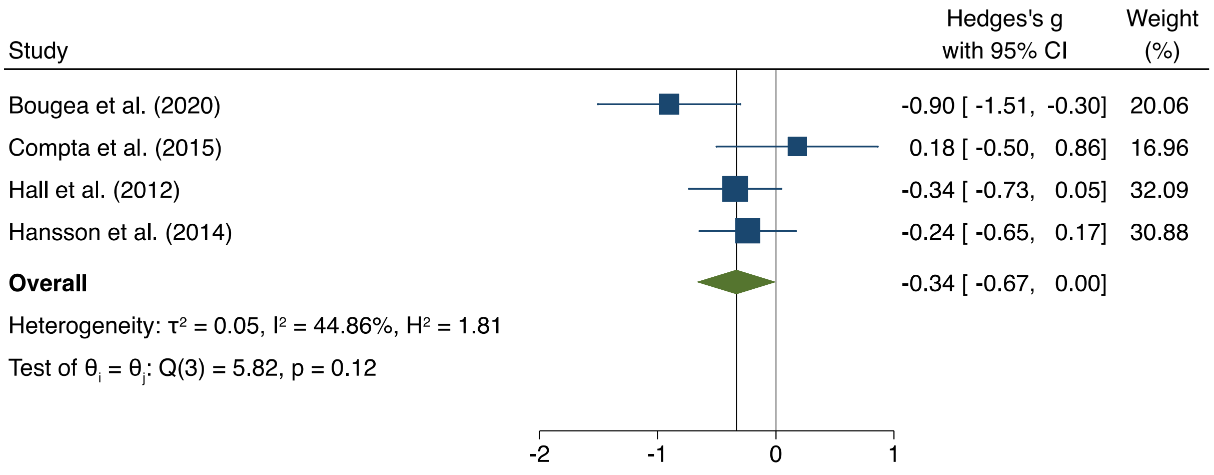

8.1.2.2. Meta-analysis of SYUA levels in CSF of people with PDD, when compared to people with Alzheimer's disease (AD).

Our random effects meta-analysis showed that CSF SYUA levels were significantly lower in people with PDD, when compared to those of people with AD (SMD = -0.83; 95%CI -1.58 – -0.07; p=0.03). We provide the funnel and forest plots of the meta-analysis below.

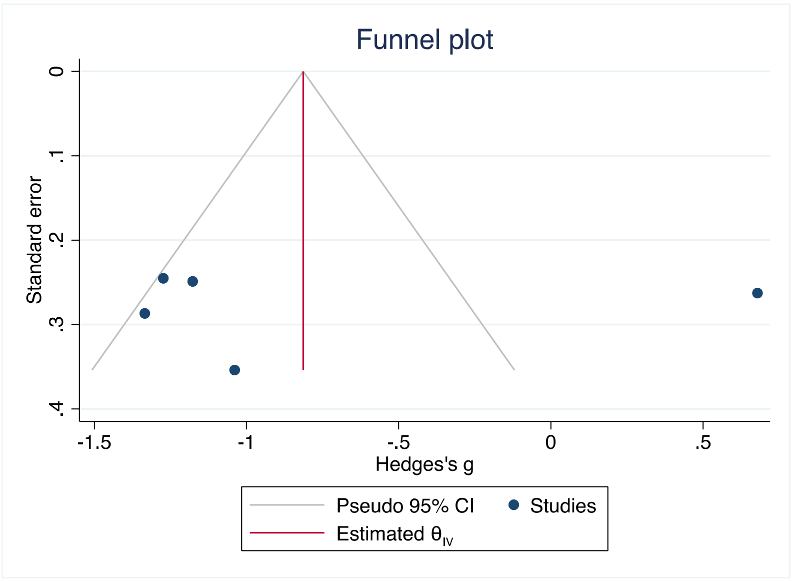

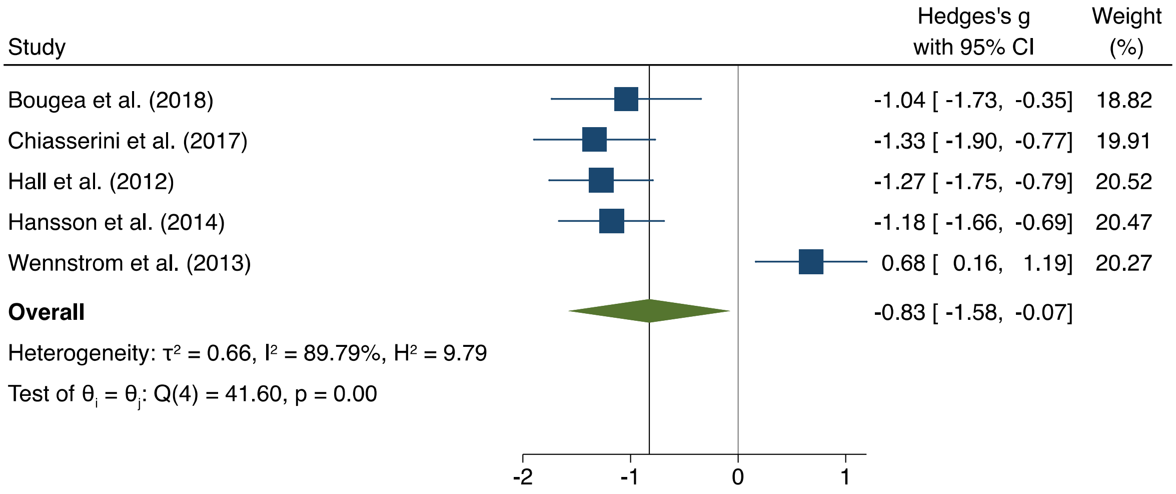

8.1.3. Neurofilament light polypeptide (NFL, P07196)

8.1.3.1. Meta-analysis of NFL levels in CSF of people with PDD, when compared to healthy controls

Our random effects meta-analysis showed that CSF NFL levels were significantly higher in people with PDD, when compared to healthy controls (SMD=1.09; 95%CI 0.86 – 1.32; p<0.01). We provide the funnel and forest plots of the meta-analysis below.

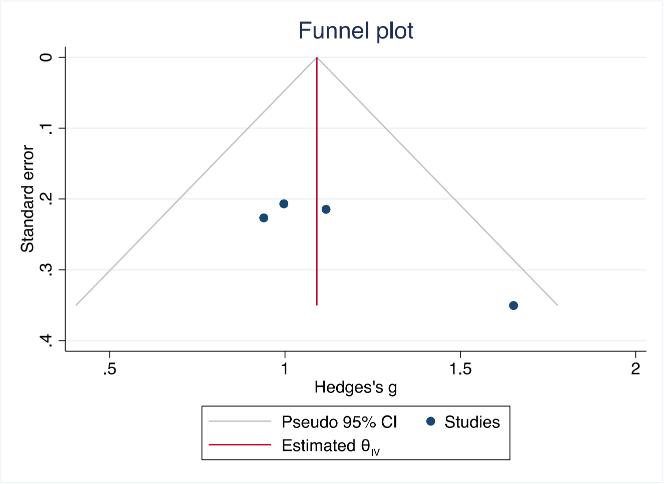

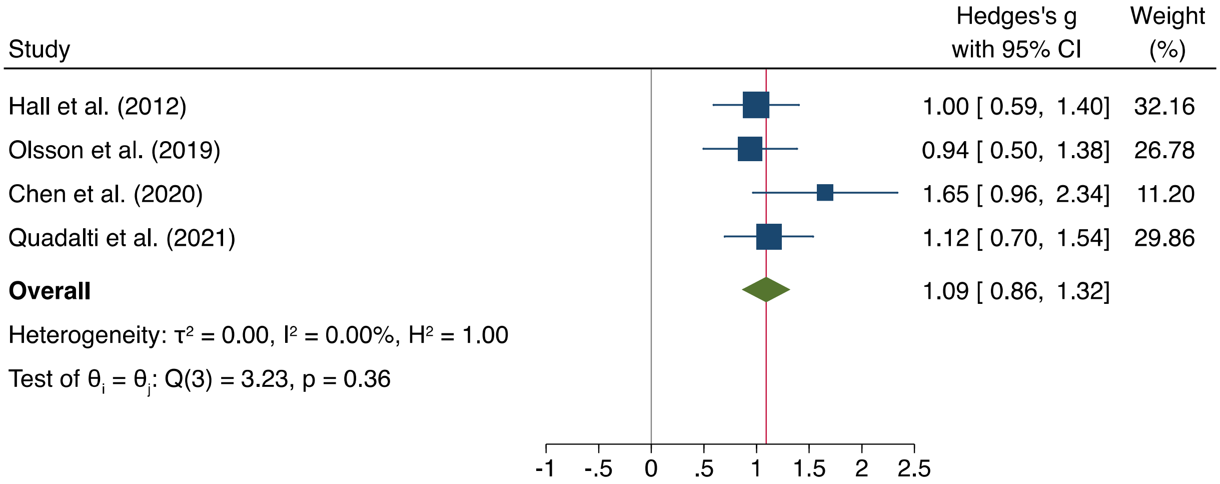
Study
Hedges's g with 95% CI
Weight (%)
Hall et al. (2012) 1.00 [0.59, 1.40] 32.16
Olsson et al. (2019) 0.94 [0.50, 1.38] 26.78
Chen et al. (2020) 1.65 [0.96, 2.34] 11.20
Quadalti et al. (2021) 1.12 [0.70, 1.54] 29.86
Overall 1.09 [0.86, 1.32]
Heterogeneity: τ² = 0.00, I² = 0.00%, H² = 1.00
Test of θi = θj: Q(3) = 3.23, p = 0.36
-1 -.5 0 .5 1 1.5 2 2.5

8.2. Post-mortem brain tissue

8.2.1. Alpha Synuclein (SYUA, P37840)

8.2.1.1. Meta-analysis of SYUA levels in brain tissue of people with PDD, when compared to healthy controls

Our random effects meta-analysis showed that SYUA levels in post-mortem brain tissue of people with PDD were not significantly different from those of healthy controls (SMD =0.18; 95%CI -1.58 – 1.93; p=0.83). We provide the funnel and forest plots of the meta-analysis below.

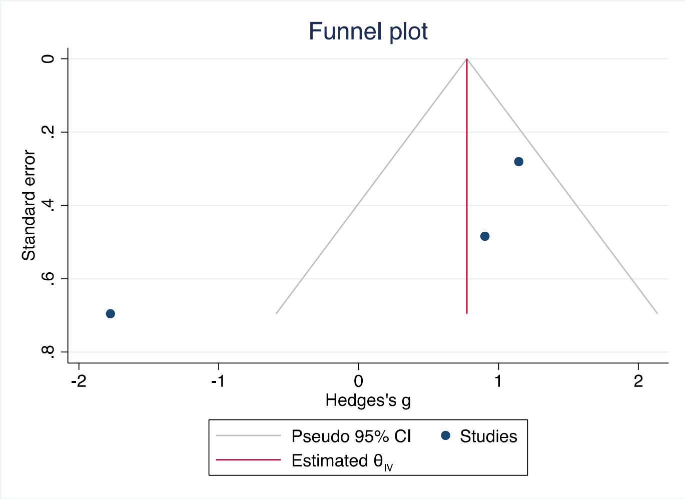

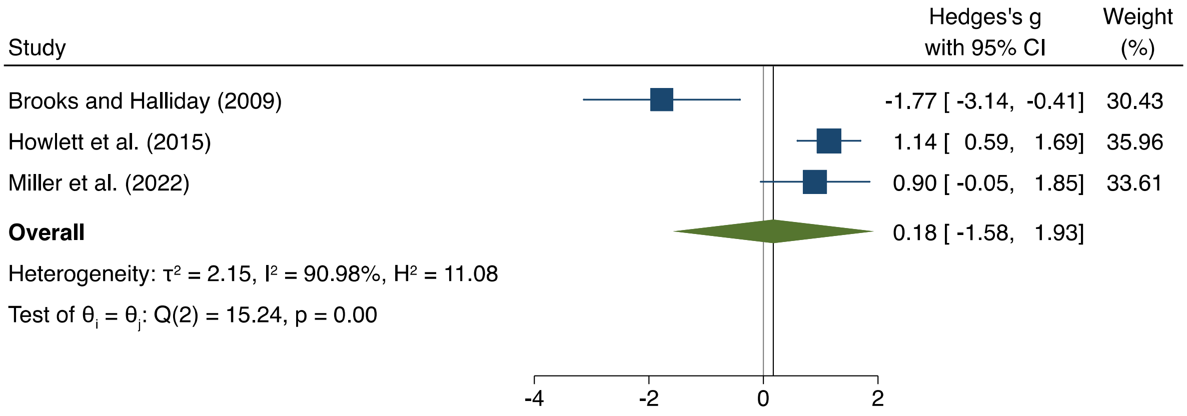

8.2.1.2. Meta-analysis of SYUA levels in brain tissue of people with PDD, when compared to people with other dementia

Our random effects meta-analysis showed that SYUA levels in post-mortem brain tissue of people with PDD were not significantly different from those of people with other dementia (SMD =2.15; 95%CI -0.42 – 4.73; p=0.10). We provide the funnel and forest plots of the meta-analysis below.

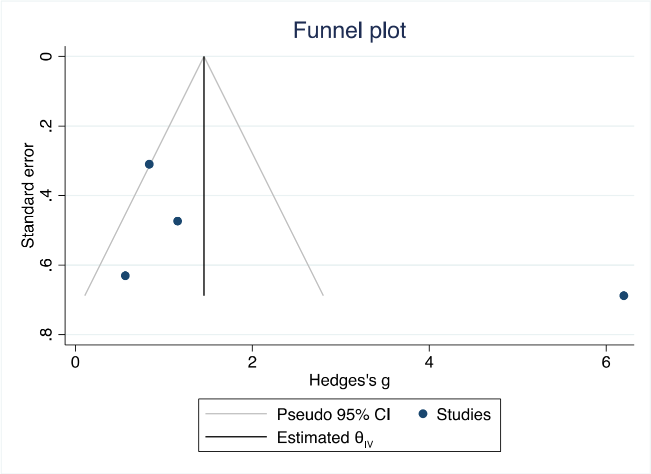

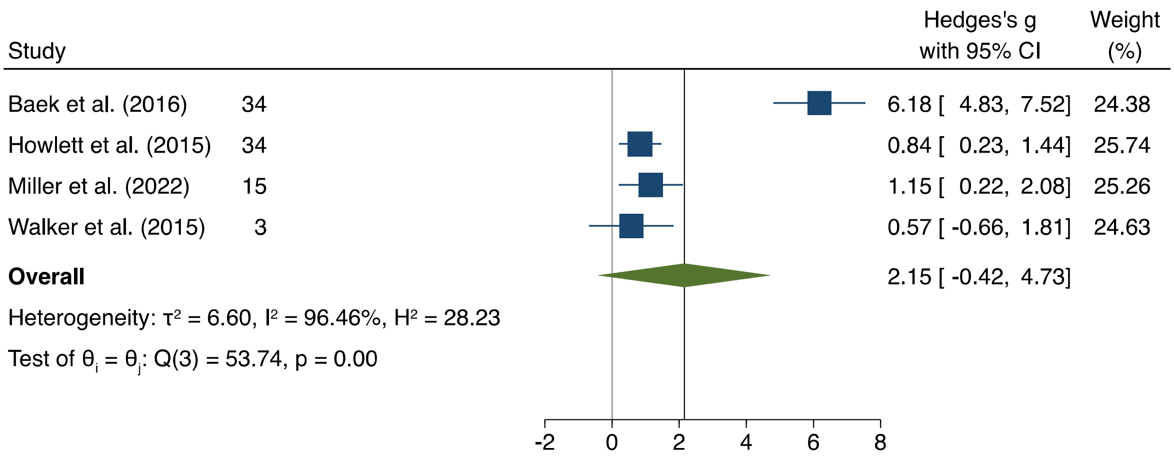

Supplement: Farr et al. supplementary material 9 — Farr et al. supplementary material [file S0924270825000158sup009.pdf]
